# Progress and Status of Hydrometallurgical and Direct Recycling of Li-Ion Batteries and Beyond

**DOI:** 10.3390/ma13030801

**Published:** 2020-02-10

**Authors:** François Larouche, Farouk Tedjar, Kamyab Amouzegar, Georges Houlachi, Patrick Bouchard, George P. Demopoulos, Karim Zaghib

**Affiliations:** 1 Center of Excellence in Transportation Electrification and Energy Storage (CETEES), Hydro-Québec, 1806, Lionel-Boulet Blvd., Varennes, QC J3X 1S1, Canada; larouche.francois3@hydro.qc.ca (F.L.); amouzegar.kamyab@hydro.qc.ca (K.A.); bouchard.patrick@ireq.ca (P.B.); 2 Mining and Materials Engineering, McGill University, 3610 University Street, Montréal, QC H3A 0C5, Canada; 3 Energy Research Institute, NTU, 1 Cleantech loop, Singapore 634672, Singapore; farouk.tedjar@ntu.edu.sg; 4 Centre de Recherche d’Hydro-Québec (CRHQ), 600, avenue de la Montagne, Shawinigan, QC G9N 7N5, Canada; houlachi.georges@ireq.ca

**Keywords:** recycling, Li-ion battery, process review, hydrometallurgy, direct recycling

## Abstract

An exponential market growth of Li-ion batteries (LIBs) has been observed in the past 20 years; approximately 670,000 tons of LIBs have been sold in 2017 alone. This trend will continue owing to the growing interest of consumers for electric vehicles, recent engagement of car manufacturers to produce them, recent developments in energy storage facilities, and commitment of governments for the electrification of transportation. Although some limited recycling processes were developed earlier after the commercialization of LIBs, these are inadequate in the context of sustainable development. Therefore, significant efforts have been made to replace the commonly employed pyrometallurgical recycling method with a less detrimental approach, such as hydrometallurgical, in particular sulfate-based leaching, or direct recycling. Sulfate-based leaching is the only large-scale hydrometallurgical method currently used for recycling LIBs and serves as baseline for several pilot or demonstration projects currently under development. Conversely, most project and processes focus only on the recovery of Ni, Co, Mn, and less Li, and are wasting the iron phosphate originating from lithium iron phosphate (LFP) batteries. Although this battery type does not dominate the LIB market, its presence in the waste stream of LIBs causes some technical concerns that affect the profitability of current recycling processes. This review explores the current processes and alternative solutions to pyrometallurgy, including novel selective leaching processes or direct recycling approaches.

## 1. Introduction

The demand for lithium ion batteries (LIBs) has increased exponentially since their commercialization 30 years ago. Worldwide sales of LIBs increased to the energy equivalent of 120,000 MWh in 2017 [1], which corresponded to the annual energy consumption of approximately 6000 houses considering the average annual energy consumption of 20.3 MWh. (Data source: Statistics Canada; Average household energy consumption for the Province of Quebec in 2015) [2]. Accordingly, the increasing popularity and use of LIBs led to an increase in the amount of spent batteries that have to be managed. This hazardous waste has to be treated appropriately and kept away from the standard household waste collection streams to avoid the possible release of toxic compounds in the environment during landfill or incineration [3,4] and to prevent fire hazards in municipal solid waste (MSW) facilities and transport equipment caused by battery self-ignition [5]. Recycling spent LIBs promotes the recovery of valuable metals while neutralizing hazardous compounds, such as toxic metals, organic solvents, and fluorinated electrolyte [6]; it also diverts a significant amount of waste from the MSW management system. The recovered materials after proper purification/refining could be reused as components for manufacturing new batteries, hence closing the circular economy loop. However, LIB recycling processes are costly and require considerable amounts of energy [7] or reagents. Well-established battery recycling plants such as Umicore, one of the largest recyclers in Europe, revealed that “the profits from selling recovered metals are not major drivers for [their] recycling operation” and that they charge service fees to battery manufacturers or collectors [8]. In addition, recycling generates secondary toxic gaseous emissions and solid waste residue and may also release contaminants to water effluents. All these potential pollution sources have to be properly managed [9]. Moreover, most current pyrometallurgical processes do not recover Li, which is a critical resource that requires sustainable exploitation [4,10,11,12,13]. Therefore, the development of new approaches based on hydrometallurgical or electrometallurgical techniques is essential for reducing the environmental footprint, increasing process efficiency, and improving the profitability of LIB recycling. Several researchers have developed alternative chemical treatment methods and have reported good recovery rates and reduced hazardous gas emissions. However, such methods still require the use of expensive reagents and considerable amounts of water [4]. Conversely, other new selective and non-destructive methods opened the possibility for regenerating the electroactive materials, which could subsequently be directly reused for manufacturing new LIBs. This low-cost approach, referred to as “direct recycling”, could result in value-added products, but could be sensitive to many parameters, such as battery sorting, extensive pretreatment, the accumulation of defects and impurities in the active materials, variations in cell composition, and battery state-of-health. Moreover, because it was designed for specific battery types, direct recycling could be more sensitive to market variations and the evolution of new battery chemistries.

The estimated recycling rates reported in the literature fluctuate significantly; although they are highly variable from one geographical region to the other, they have still been low [14]. For example, Call2Recycle, the main organization responsible for the collection of end-of-life LIBs in Canada, reported collecting less than 200 tons of LIBs in 2017 [15]. While Gies et al. determined that this quantity corresponded to a collection rate of 25% [8], we believe that their assessment was an overestimation considering the sale volumes of electronics and electric vehicles (EV). As bench marking, based on electronic device sales statistics, Wang et al. estimated the North American recycling rate was lower than 10% in 2012 [14]. As another comparison, the collection rate reported by Eucobat in Europe in 2016 was slightly higher than 15% [16]. Considering these data and the inability of the current pyrometallurgical processes to recover Li, Li recycling from spent LIBs is estimated to be lower than 1% [17], which is obviously not in agreement with the principles of sustainable development. Therefore, it is imperative to increase both the collection and recycling rates of LIBs.

This paper reviews the hydrometallurgical and direct recycling processes for Co-, Ni-, and Mn-rich batteries developed to date, but also emphasizes the treatment processes for lithium-iron phosphate (LFP) electrodes. This type of LIB, which was developed by John Goodenough in the late 1990s and has been commercialized since 2006, has not raised the same interest for recycling as the other LIBs. The low value of the elements comprising the active material of LFP electrodes makes its recycling hardly economical. However, the toxicity, environmental risk, and sustainability considerations require the development of suitable treatments for these batteries as well. In the first section of this review, we provide a description of LIBs and key resources to achieve an in depth understanding, and also the typical cycling criteria for the five major LIB chemistries. In the second section, the pertinence of LIB recycling is discussed using life-cycle analysis (LCA) as supporting data. Then, various recycling approaches are defined, and the graphic concept of circular economy specifically applied to LIBs is presented. This is followed by the description of the current industrial applications of LIB recycling worldwide. In addition, some pretreatment processes are described prior to exhaustively reviewing the hydrometallurgical processes developed to date. As such, topics including battery sorting, current collector separation, binder removal, and carbon recovery are presented. The subsequent hydrometallurgical section is divided according to the type of leaching agents, namely, H_2_SO_4_, HCl, and HNO_3_, and also other inorganic and organic acids. Each subsection provides a critical review of the processes developed so far, major chemical reactions involved, and typical flow charts. Considering the significant number of publications that we reviewed, the information for each process is summarized in large tables supplied in the Supplementary Information section. Furthermore, another section is dedicated to direct recycling techniques. Again, a table summarizing these processes is provided in the Supplementary Information. Lastly, the challenges and opportunities of the LIB recycling industry are discussed.

## 2. Description of LIBs 

Li-ion batteries comprise a wide variety of electrochemical systems that feature different anode and cathode materials, and also various electrolytes and other components. Five major types of cathodic compounds are commercially available, namely lithium-cobalt oxide (LCO), lithium-nickel-manganese-cobalt (NMC), lithium-manganese oxide (LMO), lithium-nickel-aluminum oxide (NCA), and lithium-iron phosphate (LFP). According to Avicenne Energy, in 2015, NMC represented the largest percentage of the worldwide LIB market (~29%), followed by LCO (26%) and LFP (23%) [18]. In China, LFP represented the dominant cathode material for LIBs: Approximately two-thirds of the market, or 74,400 tons in 2017 [19]. Anodic materials are also diverse; graphite is the most important anode material, lithium-titanate (LTO) is used for specific applications, and metallic Li is expected to expand its marketability in the next decade. This diversity of technologies and its rapid evolution represent challenges for the design and operation of recycling processes [6,11]. 

All electrochemical cells are governed by redox reactions that are similar to those described below for the LCO (*LiCoO*_2_) cathode and graphite anode:

(1) $$LiCoO_{2}\;⇄\;xLi^{+}+Li_{1-x}\left(Co_{1-x}^{3+};Co_{x}^{4+}\right)O_{2}+xe^{-}$$

and

(2) $$xLi^{+}+xe^{-}+6C\;⇄\;Li_{x}C_{6},$$

where the *Li* inserts into the graphite layers are reduced to *LiC*_6_ compound [20]. 

Table 1 summarizes the general electrochemical properties of the most common cathode active compounds. More detailed information for all technologies could be found in the literature [21,22]. Recycled cathodic materials should meet these properties. 

Both cathodes and anodes consist of pastes of intercalation compounds bound together with organic binders such as polyvinylidene fluoride (PVDF) and are coated on electronic conductors. Typically, Al and Cu foils are the preferred cathode and anode current collectors, respectively. Electric insulation between electrodes is maintained using an ion-permeable membrane and an electrolyte, which is a good ionic conductor. The most common electrolyte used for LIBs consists of a solution of lithium hexafluophosphate in alkyl carbonate solvents, such as ethyl carbonate or dimethyl carbonate. Lastly, alternating cathode, anode, and membrane layers are rolled or folded together, and then are wrapped into solid- or soft-shell casings. 

**Figure 1:**
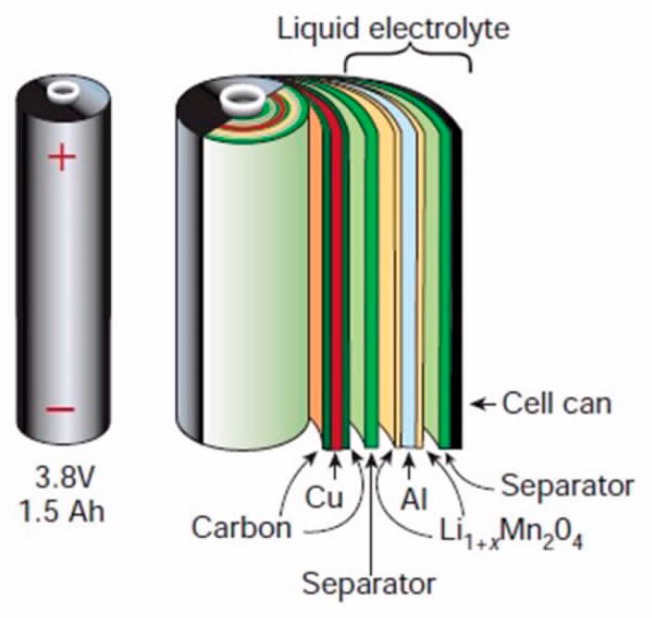
Cylindrical cell details ((reproduced with permission from Springer Nature Ref. [26]).

**Figure 2:**
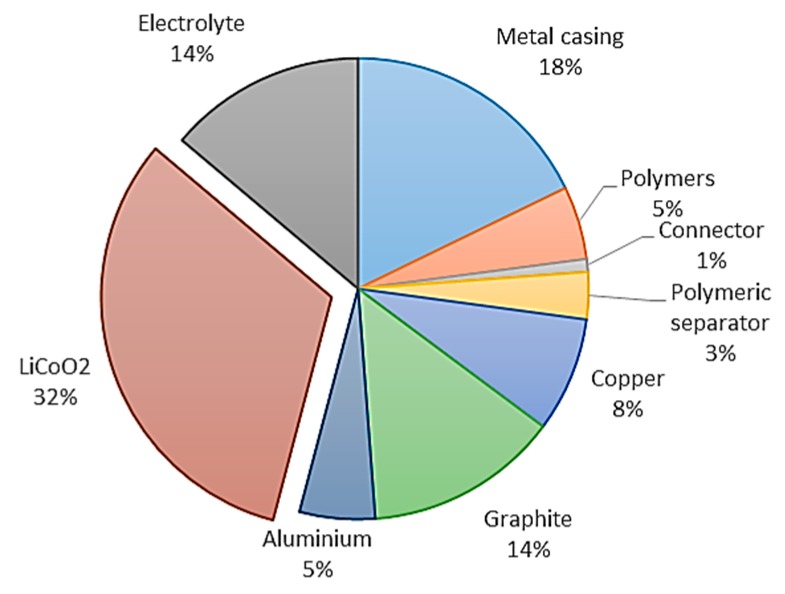
Bill of materials of lithium-cobalt oxide battery (wt.%) (based on data provided by Silveira et al. [27]).

The schematic illustration of a cylindrical cell configuration is presented in Figure 1 [26]. Moreover, a pie chart that presents the typical composition of LCO is presented in Figure 2 based on data provided by Silveira et al. [27]. Gaines et al. provided the detailed cell composition of the major types of LIBs [10]. The composition of spent LIBs is slightly different than that of fresh ones. The spent cathode material is usually not fully lithiated and could have undergone some structural changes during cycling. In addition, the electrolyte partially decomposes and/or evaporates during aging, and thus, it promotes the corrosion of both current collectors. To design appropriate recycling processes, particularly direct recycling processes, it is important to fully understand the aging mechanisms of LIBs, and Birkl et al. provided a great comprehensive paper on aging mechanisms [28]. 

## 3. Recycling as Sustainable Solution for LIB Waste Management 

Considering the average specific energy (Average capacity of the five major types of Li-ion considering only 18,650 cells format) of 180 Wh/kg [29] and the global LIB market of 120 GWh [1], the weight of the LIBs sold in 2017 was estimated to be approximately 670,000 tons. Once reaching the end-of-life, spent batteries will generate large amounts of hazardous waste that should be managed and treated. The disposal of spent LIBs using the standard MSW management system poses great safety risks as wasted LIBs could catch fire or explode owing to internal short-circuiting leading to the evolution of O_2(g)_ from the decomposition of cathodic material, the fast release of heat, and ignition of organic solvents [30]. Consequently, fire incidents caused by LIBs frequently occur in waste management facilities [3], and have caused up to 65% of the fires in waste facilities in California in 2017 [31]. The Environmental Services Association attributed a quarter of the fire incidents in the UK waste facilities to spent LIBs [32]. Furthermore, fire incidents caused by LIBs have been reported occasionally in landfill operations [3]. These incidents represent major safety risks considering the generation of heat, evolution of methane gas, and presence of combustible materials. The transport regulations for LIBs have been summarized by Gaines et al. in their review paper on LIBs recycling [10]. 

Spent LIBs represent a large environmental risk owing to the fluorinated components, organic solvents, nanoparticles, and leachable metals in their composition [3,6]. First, to minimize these risks, proper gas cleaning systems should exist in all recycling facilities to minimize the emission of fluoride and organic volatile compounds during battery crushing, pretreatment, and smelting [4]. However, the costs of off-gas cleaning systems for pyrometallurgical process plants are high [10]. Second, caution should be exercised regarding the propagation of nanoparticles in air and water sources. The crushing and heat treatment of LIBs promote the release of nanoparticles, which could present significant ecotoxicological effects [33]; thus, pyrometallurgical process plants should be equipped with off-gas cleaning systems. The waste effluent of hydrometallurgical processes should be properly treated (using coagulation and filtration) to avoid the dispersion of nanoparticles and toxic soluble compounds. Lastly, the disposal of spent LIBs in landfills raises concerns as the toxic and heavy metals in these LIBs, such as Cr, Co, Cu, Li, Mn, Ni, Pb, and Tl [3], could contaminate ground water sources. 

The rapid increase in the production of LIBs puts pressure on the environment and natural resources, particularly Li and Co ones. In fact, 35% and 25% of the global Li and Co production, respectively, are used for manufacturing LIBs [9]. Recycling LIBs lessens the demand for raw material, as reported by Gaines et al., and thus could render LIB manufacturing more sustainable [10]. However, Gaines et al. also indicated that the recycling industry could not currently meet the demands for natural resources owing to the long lifetime of LIBs and their exponential market growth [10]. We also believe that the impact of recycling on the demand for raw materials will be limited as long as recovery and collection rates remain very low. 

**Figure 3:**
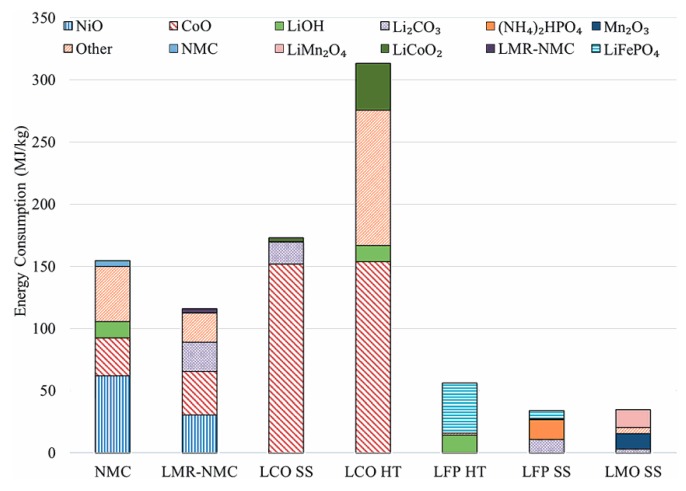
Energy consumption for different synthesis methods and various active materials (reproduced with permission from The Royal Society of Chemistry Ref. [38]); here, NMC, LMR-NMC, LCO, and LFP are lithium-nickel-manganese-cobalt oxide, Li and Mn-rich lithium-nickel-manganese-cobalt oxide, lithium-cobalt oxide, and lithium-iron phosphate, respectively; and SS and HT are solid state and hydrothermal synthesis methods.

**Figure 4:**
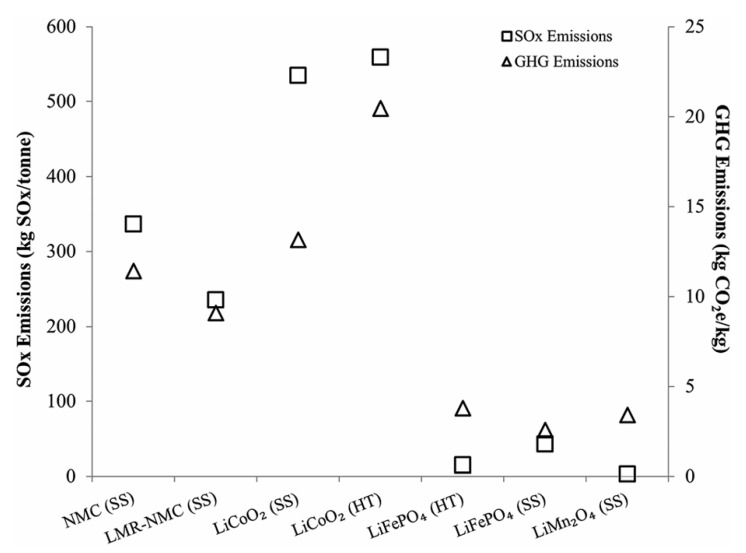
Gas emissions for synthesis methods for various active materials (reproduced with permission from The Royal Society of Chemistry Ref. [38]); here, NMC and LMR-NMC are lithium-nickel-manganese-cobalt oxide and Li and Mn-rich lithium-nickel-manganese-cobalt oxide; SS and HT are solid state and hydrothermal synthesis methods; and GHG is greenhouse gas.

Many studies have used LCA techniques and confirmed that the impact of EV manufacturing on the environment and depletion of mineral resources is higher than that of fossil fuel-powered vehicle manufacturing [13,34]. This difference is mainly attributed to the battery manufacturing processes, which could account for approximately 20% of the total energy consumed for manufacturing EVs and approximately 40% of the CO_2_ released by EVs during their entire life [35]. More specifically, the production of cathodic materials, particularly for Ni- and Co-based cathodes, is the most impactful step during the manufacturing of LIBs [36,37,38,39]. Table 2 summarizes the global warming potential associated with the production of 1 kg LFP active material calculated using LCA data provided in the listed references. The energy impact of cathodic material manufacturing has been reported to range from 19 to 56 MJ per kg of LFP [38,39]. The charts in Figure 3 and Figure 4, which were reproduced from Dunn et al. (2015), compare the process costs and greenhouse emissions of several cathode materials [38]. Therefore, post-consumption LIB recycling could reduce the life cycle impact of EVs by up to 51% [14,38] and deviate a significant amount of waste from MSW facilities [40]. This could also represent a great opportunity for boosting local economies as long as circular economy principles are applied.

## 4. Recycling Approaches

Recycling of LIBs has been studied as early as after the commercialization of LCO batteries by Sony in 1991 [21]. The first work on LIBs recycling at pilot level started in 1995 by Recupyl with support from French Environmental Agency. The installation obtained an agreement on behalf of French Authorities in 1998. Recupyl’s work continued with European Union support under the Valibat project in collaboration with Taridan, the French Alternative Energies and Atomic Energy Commission, Rhodia, and Sedena. In the meantime, the first academic paper on recycling had been published by Zhang et al. in 1998 [42], and the number of such publications continuously increased since. More than 20 years later, LIB recycling still attracts the attention of scholars [6]. However, the interest for spent LIBs is mainly limited to the extraction of Co, Ni, and Mn [4]. Other materials, such as Li and FePO_4_, cannot be recovered using the current standard pyrometallurgical methods [10]. Nevertheless, processing of LFP has been studied by some authors as either a distinct process or a general one that encompasses all LIB chemistries. For example, the selective leaching process developed by Zou and Gratz could be used to treat mixtures of NMC, LCO, LMO, and LFP [43]. The patent published by Recupyl in 2005 focused on the treatment of Mn-, Ni-, and Co-rich batteries; however, it could be adapted to manage spent LFP as well [44,45,46]. 

**Figure 5:**
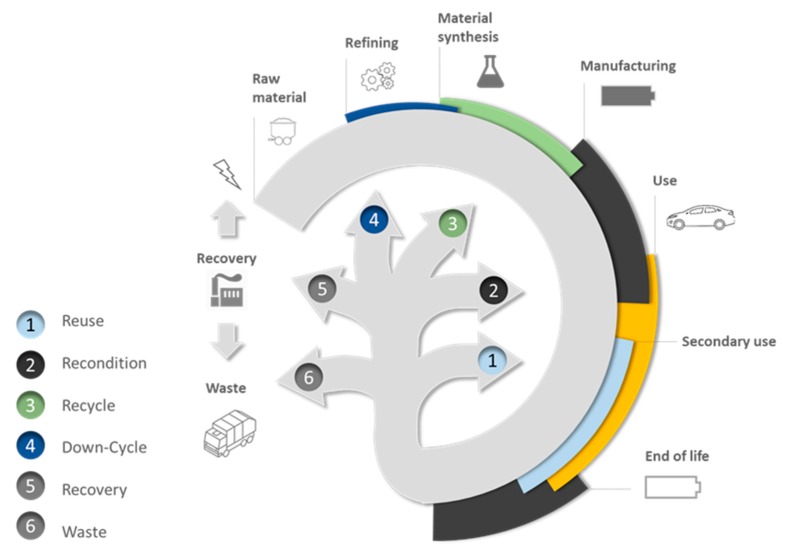
Cyclic flow chart of manufacturing, usage, and end-of-life of Li-ion batteries.

Conversely, recycling is one of multiple branches of the waste management cycle, which is promoted by environmental agencies worldwide under the “5R’s” acronym. Although the definition of this acronym may vary, the five “R’s” typically stand for reduce, reuse, recycle, recover, and residual management. The flow diagram in Figure 5 illustrates the 5R’s concept for the life cycle of LIBs starting the manufacturing loop from raw material extraction to battery manufacturing then following with use stage until battery reaches its end-of-life. Zhang et al. proposed a simplified “3R’s” version of the cycle, where the three “R’s” stand for redesign, reuse, and recycle [4] while Harper et al. [47] detailed the origin of this waste management hierarchy and defined each level in perspective to LIBs. 

Particularly, the “recycling” branch covers an entire range of methods including “recondition” 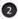, “recycle” 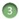, and “down-cycle” 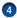. Although the general objectives of recycling are to maximize the material recovery and economic value while minimizing the environmental impact, it also includes processes that focus on recovering only one target component from the whole battery system or degrade LIB components to different degrees of lower value products [48]. The term “downcycling” can be used to refer to this degradation process, whether deliberate or unintended, and is also designated as “open-loop” recycling in the literature [49]. The quality and inherent properties of a product that enters an open-loop recycling chain are undoubtedly altered. This arises from the cross-contamination with other type of materials, mixing various additive elements contained in similar materials, or the degradation of the physical and chemical properties of the recycled material. More specifically, downcycling is unintended when the qualities of a material have been degraded owing to its manipulation and processing. Conversely, downcycling is deliberate when the purpose of the process is to return the material to its more simple or elementary form. Thus, to reverse such decrease in quality, recycling should include additional costly purification steps. For example, Ni and Co alloys are recovered when Co- and Ni-based LIBs are processed together with ore concentrate in a smelter like Glencore’s Sudbury (Canada) facilities. The Co and Ni alloy requires additional hydrometallurgical treatment before it can be used for synthesis of fresh cathode compounds suitable for manufacturing new LIBs.

**Figure 6:**
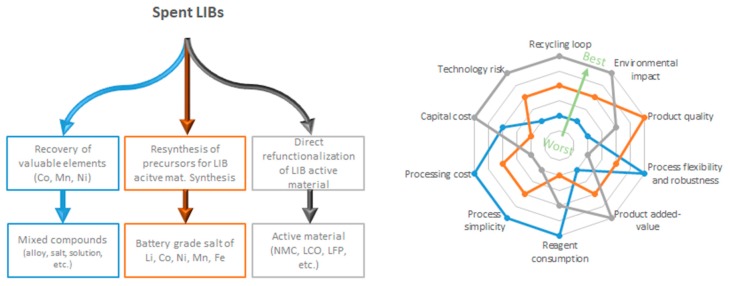
Schematic flow and comparison of three approaches for recycling spent Li-ion batteries (LIBs).

We defined three approaches to LIB recycling as described on the left side of Figure 6. The first process could be used to extract valuable elements or compounds and valorize them into lower-value products or reintroduce them in the raw material production line. These processes are of low environmental benefit but are simple and less sensitive to economic fluctuation. This is the business model adopted by Retrieve Technologies, where spent LIBs are crushed in an inert environment. Then, Li is recovered in liquid phase and is subsequently sent off-site for further treatment. The transition metal-rich solid paste obtained is sold to external refineries, such as Glencore (Sudbury, ON, Canada), where Co and Ni are recovered. Thus, the end-products no longer serve as battery material, and consequently, the process presents a high level of downcycling. Pyrometallurgical treatment frequently follows this low added-value approach. For example, SNAM (Viviez, France) produces NiFe alloys for stainless-steel manufacturing or a Co concentrate for pigment production from Ni and Li batteries [50]. 

The second approach aims to regenerate high-quality precursors for the synthesis of fresh active material for LIBs using hydrometallurgy. The products thus obtained meet the battery manufacturing specifications, and this approach follows the principles of circular economy. Most hydrometallurgical processes described in the literature follow this approach. Although no quality loss should occur during such a process, the amounts of reagents and associated energy consumption remain high. The implementation of complex purification lines to meet battery-grade specifications increase the capital and operating cost of such recycling processes. Umicore combines pyrometallurgical and hydrometallurgical processes to obtain high quality Co, Mn, and Ni salts intended for manufacturing batteries [51]. Unfortunately, during the process implemented by Umicore, Li is downgraded to a low-value cement additive. 

Lastly, the recycling loop can be shortened by directly regenerating the properties of the active materials. This is the most environmentally friendly method, but it is also more sensitive to the state-of-health of LIBs. Defects and impurities accumulated during cycling or caused by the over-discharging and improper storage of LIBs could affect the quality of the refurbished active material. Ultimately, after many cycles, the active material might have to be discarded and resynthesized because direct recycling cannot yet fully restore the initial properties of pristine active materials. Although more complex pretreatment is required to avoid cross contamination from components of other types of LIBs, this approach reduces the required amounts of energy and reagents and aims to simplify the core process, which could result in smaller and more affordable recycling plants. 

Selective hydrometallurgy is an intermediate approach between direct reconditioning and recycling. The cleaning effect of selective leaching reduces the process sensitivity to contamination and battery defects.

All three approaches are compared in the spider chart in Figure 6 using our own criteria, which were established after assessing already published information [4,6,10,11,52] and our own experience. 

### 4.1. Currently Used Recycling Processes

Several recycling plants currently operate worldwide (in China, Europe, Japan, and USA). Table 3 presents a non-exhaustive list of currently operating LIB recyclers. For many companies, such as SNAM and Glencore-Xstrata, the treatment of LIBs is an expansion of their initial Ni-based battery (Ni-Cd and nickel–metal hydride (NiMH)) recycling operations. 

Pyrometallurgy is the most common method currently used in the recycling industry by major companies, such as Umicore (Belgium), Dowa and Sumitomo (Japan), Accurec (Germany), Batrec (Switzerland), and Nickelhütte Aue Gmbh (Germany). The pyrometallurgical process is usually followed by hydrometallurgical steps to extract valuable metals from the matte, as pyrometallurgical processes cannot achieve the efficient separation of various metals. In contrast, recycling plants that use only hydrometallurgical processes are still rare, and the most important are Retriev (Canada and US) and Recupyl (France). A review of the development status of hydrometallurgical recycling processes is provided in the subsequent sections. 

Based on global LIB recycling capacity in 2016 (94,000 tons) reported by Mayyas et al. [53] and global LIB sales for 2016 (500,000 tons) (Calculated based on energy sales statistics from Pilot [54] and considering an average energy density of 180 Wh/kg), the theoretical installed recycling capacity only covered 19% of the 2016 manufacturing one, assuming that all recycling plants were exclusively dedicated to LIB recycling. For 2024, this ratio falls below 9% according to the expected LIB sales (3,000,000 tons) and the recycling capacity forecast (264,000 tons) provided by Propulsion Quebec [55].

In this section, we only address in detail the direct hydrometallurgical processes (from scrap batteries to final materials). These processes are listed in chronological order in Table 4.

The only process that has been reported to recover both Li from the electrodes and fluorine-based anions as well as Li^+^ ions from conductive salts is that implemented by Recupyl [claim 5, 11] [56]. The recovery of spent electrolytes is one of the most important challenge in the LIB recycling industry [6,11,52] and should be implemented for existing or developing processes. This important step has been increasingly studied or integrated, including in the recent pilot plant project proposed by Recyclage Lithion, where the organic carbonate solvents are captured during the automated dismantling step and are subsequently distilled into several fractions [62].

Another important aspect of recycling concerns graphite. According to the classification of the European Union, graphite is considered to be a strategic material. When pyrometallurgical processes are used, carbon and graphitic fraction are lost. However, leaching is used to obtain pure graphite/carbon fractions during the process used by Recupyl [56,57]. Once recovered, these materials are purified and can serve as graphite source in manufacturing of new anode, thus replacing primary carbon sources. Such recycling method would be a great way of implementing circular economy principles.

### 4.2. Pretreatment of Spent LIBs

Recycling plants receive spent LIBs in two types of loads:

▪ Bulk shipment of small-size LIBs, including portable ones, small electronics, and E-bike modules;
▪ Battery packs from used or crashed electric and hybrid vehicles or stationary devices.

These feeds should be handled differently owing to the difference in their size, format, and electric power. More specifically, the LIBs in the first type of loads present a wide variety of types, sizes, and chemistries. Such bulk mixtures should be sorted by format, size, and chemistry prior to any treatment. The second type of loads include large assemblies of battery modules featuring steel or Al frames, and include plastic components, thermal insulation, electric cables, electronic printed circuits, and individual cells. Usually, these packs are carefully dismantled manually into modules or even individual cells prior to recycling [4]. Such operation could expose workers to significant electrical risks because these LIBs could provide more than 450 V and 150 kW [63].

Many authors indicated that the pretreatment of spent LIBs is a key step prior to the recovery of cathode components, particularly for hydrometallurgical and direct recycling processes [40,64,65,66,67]. The objectives of the pretreatment are to maximize recovery of valuable materials, reduce the flow rate of material undergoing the downstream process, ensure the safe disposal of hazardous components, and ensure the safe handling of spent LIBs. This latter aspect is particularly important because it could help reducing the safety risks (high fire risks) and consequently decrease transportation costs of spent batteries, which could represent 40%–50% of the overall recycling cost [68]. Two strategies could be used to reduce transportation costs significantly. First, companies might consider designing several smaller local process plants, which would shorten travelling distances. In addition, they could use several remote (or mobile) small crushing facilities to feed the central processing plant for black mass treatment. Thus, only black mass needs to be transported, which neutralizes all safety concerns. 

Pretreatment processes could be grouped into three categories: Physical, chemical, and thermal [69,70]. More specifically, the typical pretreatment steps could be identified according to the list below; these steps could be used separately or could be combined:

▪ Battery pack dismantling,
▪ Sorting by chemistry,
▪ Discharging,
▪ Crushing and shredding,
▪ Material separation,
▪ Electrolyte recovery,
▪ Binder separation,
▪ Thermal treatment,
▪ Washing.

After pretreatment, the active material could take different forms according to the degree of liberation achieved. This is indicated in the “Leach Feed” columns in Tables S1, S3, S5, S7, and S9 (Supplementary Materials). Therefore, we propose the following scenarios depending on the state of the leach feed material:

1. Combination of anode and cathode; the feed material comprises Cu and Al current collectors, graphite from the anode, carbon from the cathode, PVDF (or other plastic binder), and cathodic active material;
2. Cathode only; the feed material includes Al, C, PVDF, and active material;
3. Black mass; Al and Cu are removed mechanically or manually up-stream or dissolved in strong alkaline media, but PVDF and conductive carbon remain in the black mass. Generally, the anode is first separated from the cathode;
4. Active material; either the binder material (mostly PVDF) is dissolved in solvent or is thermally degraded prior to leaching. In the latter case, the temperature of the thermal treatment determines the degree of degradation of PVDF and conductive carbon;
5. Calcined black mass; active material has been subjected to high temperature in oxidizing environment, and consequently the binder and carbon burned, and the inorganic compounds were oxidized.

Typical composition of some spent cathodic materials is presented in Table 5. The presence of Cu and Al in the black mass could be attributed to either pieces of current collectors that were entrained in the sample during the preparation of the black mass or the metal plating on the cathode surface owing to the redox processes that could have occurred at both electrodes during over-discharging [71].

### 4.3. Battery Sorting

Even though most studies on the hydrometallurgical processing of LIBs were performed on feed material that contained only LIBs, several research groups developed processes for treating streams of mixed spent batteries. Kulchaya et al. considered a blend of alkaline, Li-ion, Li primary, and Ni-based secondary batteries as feed material, but achieved low recovery [78]. Xi et al. investigated the H_2_SO_4_ leaching process and treated a mixture of NMC and NiMH batteries to produce Ni-Co ferrite [79]. Unfortunately, although a high-value product was obtained, such process did not provide a closed-loop solution that would allow recycling spent active material into new batteries. 

Nevertheless, individual cells could be sorted manually or using mechanical sorters according to the composition of the batteries. This classification is very important to avoid any cross-contamination of the downstream metallurgical processes, particularly for the bulk delivery of spent batteries where alkaline batteries, Li primary cells, LIBs, and NiMH are mixed together in various proportions. Sorting is easier for large battery packs because they only contain single chemistry batteries. Because the state-of-charge and state-of-health of these accumulators are unknown, safety measures should be implemented to protect operators performing this task.

Although perfect separation cannot be achieved, current practices and recent technologies indicated that it is feasible to efficiently sort a stream of mixed spent batteries, as stated in a report published in 2000 by the European Portable Battery Association [80]. Sortbat (Tienen, Belgium) implements manual pre-sorting and residue scrubbing operations followed by mechanical and automatic sorting of batteries by size and chemistry [81]. The automatic sorting is based on the magnetic resonance response of the batteries, and seven types of batteries can be separated, including LIBs. The purity achieved could reach up to 99.7% [82]. Other types of sorting machines include X-ray and optical sorters or automatic label readers [81]. The nominal capacity on automatic sorters is approximately 5–24 cells/s (approximately 500 kg/h to 3 t/h) [83]. 

To our knowledge, no automatic sorting system that could sort various types of LIBs according to their chemistry is commercially available. In addition, the next-generation materials, such as LFP or Li_2_MSiO_4_, could further hinder the design of such sorters. Moreover, sorting spent LIBs could even be more difficult because LIBs of the same chemistry could have various intrinsic compositions owing to doping, binders, electrolytes, anode materials, etc. [84]. To manage these challenges, a research group from the Worcester Polytechnic Institute has developed a process that included all major types of LIBs, which generated a final refurbished NMC cathodic material and iron phosphate residue [43,85]. Similarly, Huang et al. developed a process suitable for treating a mixture of low-value cathodic materials, namely LMO and LFP [86]. However, such processes could be very sensitive to variations in feed composition and would most probably require a proper feed management system, including storage and blending to homogenize the proportions of both types of batteries. In addition, the development of new battery chemistries (different anode or cathode materials) could significantly affect the efficiency of the process, and thus, it would require major modifications or the implementation of pre-sorting. Lastly, the hydrometallurgical treatment of mixed battery streams requires a complex flowsheet to produce high grade products. Considering the sorting technology gap, wide variety of LIB compositions, and cost of automatic sorters, the recycling of blended portable LIBs will most probably continue to be performed in facilities able to treat a stream of mixed cathode materials that use pyrometallurgical or complete leaching hydrometallurgical processes (such as Recupyl).

Conversely, recycling large battery packs is suitable for processes adapted only for one type of LIB. This allows the design of much simpler processes or the implementation of direct recycling method. Such individualized processes are the most commonly recycling methods described in the literature. 

### 4.4. Size Reduction and Component Separation

Two main paths can be distinguished for the preparation of spent batteries prior to recycling: The entire cells, modules, or even battery packs are crushed; or the casing of the cells is cut opened, which provides access to the jelly roll. The former method is easier to implement industrially than the second one and is currently used by Recupyl and Retrieve Technology. Al-Thyabat et al. compiled a good review of crushing and screening studies including size-by-size analysis [87]. Zhang et al. used X-ray diffraction (XRD) measurements to perform size-by-size analysis of wet and dry crushing products and concluded that dry crushing better segregated the active material [65]. This could be easily explained using the form factor of the crushed battery components. While the more brittle cathodic and anodic active materials brake into smaller fractions upon crushing, the more ductile metallic or plastic compounds (namely Cu and Al foil and the separator) maintain their flat shape and tend to remain on the mesh of the screen. Typically, a screen size of 0.6 mm appears to be advantageous for size segregation and can be easily scaled-up for industrial applications.

### 4.5. Removal of Current Collector and Binder

Many methods have been developed to separate the active material from the current collector and binder. Some researchers prefer decomposing the binder to allow the detachment of the current collector and liberation of active material. This can be achieved thermally or chemically using solvents. Other scholars would rather only remove the Al sheet via either chemical dissolution or mechanical delamination, which would cause the active material to remain entrapped in the binder. Lastly, some scientists perform selective leaching of the entire cathode and remove Al and the binder at the end of the process [77].

**Figure 7:**
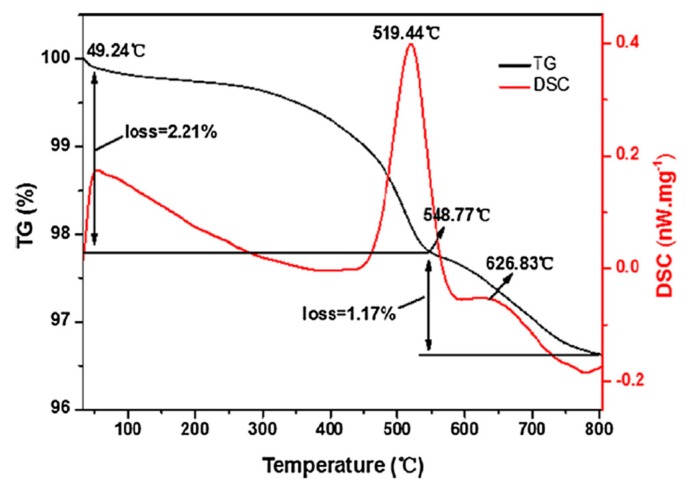
Thermogravimetry-differential scanning calorimetry (TG-DSC) curves of spent laptop Li-ion batteries in air (reproduced with permission from Springer Nature Ref. [88]).

The objective of the thermal treatment is to improve the leaching efficiency by either removing or decomposing the carbon and binder and by oxidizing or reducing the active material to a more soluble form. Hence, Chen at al. used thermogravimetry-differential scanning calorimetry (TG-DSC) to determine the optimal temperature (550 °C) that would lead to the decomposition of the binder during the thermal treatment of LCO in air [88]. As illustrated in Figure 7, the PVDF binder started to decompose at approximately 300 °C and its decomposition continued until 549 °C, when the carbon also started to decompose [88,89,90]. The high exothermic peak at 519 °C corresponded to PVDF and the decomposition of carbon. A reducing atmosphere or vacuum pyrolysis would lower the valence of Co, Ni, and Mn, which would, thus, increase their solubility [4]. For example, Yang et al. concluded that thermal treatment in the range of 550–650 °C in inert atmosphere would increase the efficiency of H_2_SO_4_ leaching [91]. The XRD results indicated the reduction of Ni ions from NMC to metallic Ni, while the Co^3+^ and Mn^3+^ ions appeared to maintain their trivalent form. Similarly, Li et al. observed the presence of NiO and Ni^0^ in NMC samples that underwent vacuum pyrolysis [92]. However, comparison of reductive or inert thermal treatment process with other type of pretreatment did not lead to a significant enhancement in leaching performance, with most processes reaching more than 95% extraction of Li, Co, Ni, and Mn independent of the pretreatment conditions applied. 

For the LFP cathode, presence of air would easily oxidize ferrous to ferric, decompose polymer binder, and burn the carbon conductive layer, making Li more accessible for leaching without any oxidizing agent [93]. Jie et al. analyzed the thermal decomposition of spent LFP cathodes in the presence of oxygen using TG-DSC and X-ray diffraction spectrometry (XRD), scanning electron microscopy (SEM) coupled with energy dispersive spectrometer (EDS). They suggested that the following oxidation reaction of iron phosphate occurred in the temperature range of 476–487 °C [94].

(3) $$12LiFePO_{4\left(s\right)}+3O_{2\left(g\right)}\to 4Li_{3}Fe_{2}{\left(PO_{4}\right)}_{3\left(s\right)}+2Fe_{2}O_{3\left(s\right)}$$

This reaction was also proposed by Zheng et al., who determined the optimal roasting temperature to be 600 °C resulting in complete decomposition of polymer binder and oxidation of *Fe* and C coating while avoiding aluminum foil degradation. The sintering product could be easily detached and sorted from Al current collector prior to leaching [93].

For the direct recycling method, the thermal treatment is performed under inert atmosphere to avoid the oxidation of the active material. Gaabour studied the decomposition of PVDF and poly (ethylene oxide) mixed with carbon nanotubes under N_2_ atmosphere and demonstrated that PVDF underwent carbonization from 300 to 500 °C [95]. These results were in agreement with those reported by Zucolotto et al. who suggested a two steps mechanism starting with the evaporation of HF followed by carbon chain scission [96]. Beyond the decomposition of the binder, thermal treatment allows the destruction of other organic contaminants, recrystallization of the active material, and regeneration of the carbon coating [69,97,98]. According to Kim et al., the carbonization of the binder during the thermal treatment of LFP black mass in N_2_ atmosphere increased the electrical conductivity of the active material and enhanced its electrochemical performance [69]. This process will be further discussed in the section on direct recycling. 

The removal of the PVDF binder could be performed via dissolution in N-methyl-2-pyrrolidone (NMP) at 80–100 °C [4]. Once PVDF was dissolved, the active material easily separated from the Al current collector. While highly toxic, NMP is the most efficient solvent for PVDF, as reported by Yang et al. [99] after they compared the efficiency of several solvents. Particularly, coupling NMP with sonication helps to detach the coating from the Al substrate [99,100]. Ionic liquids have been used by Zeng at al. as alternatives to NMP for binder dissolution [101], and Zhou et al. reported good separation using N-N-dimethylformamide at 60 °C [102].

Alternatively to binder dissolution, the black mass coating from spent LCO batteries was successfully detached from the current collector by immersing the current collector in water at 55 °C in an ultrasonically agitated bath [103]. Yang et al. used the entire LFP electrode for the selective leaching process and reported that the coating detached from the Al sheet under agitation in the leaching reactor [77]. In both cases, the recovered active material was still trapped in the PVDF binder, which could weaken the diffusion of the ions and leaching agent during the dissolution process.

Aluminum could also be dissolved in NaOH, as demonstrated by several authors [72,75,89,104]. Advantageously, this technique could completely remove Al including the small micro fragments generated during the cathode shredding and cutting from the black mass sample. Typically, a 10% (*w*/*v*) NaOH solution allows to reach 98% dissolution after 5 h [105]. Ren at al. studied the effect of Al impurities on the capacity of regenerated NMC cathodes [106], and concluded that no significant effect was observed when the molar ratio of Al/(Ni + Co + Mn) was below 3 to 100, but capacity could decrease by 40% if this threshold was exceeded. In addition to Al current collector dissolution, a pre-leaching step may be allowed to remove plated Cu contaminating Li-ion cathode. Hence, the addition of 5 M NH_4_OH to 1 M NaOH created strong complexing conditions, which facilitate dissolution of both Cu contaminants as soluble copper ammonium complex and Al current collector [107].

### 4.6. Graphite Separation

The extraction of graphite from the mixture of shredded anode and cathode can be performed via selective flotation. This common technique is part of the processes patented by Retriev Technologies [60] and Warner Babcock Institute for Green Chemistry [108]. The effectiveness of flotation was studied by Yu et al. who used n-dodecane as collector [109] and generated a 97.2% LCO concentrate. Alternatively, He et al. proposed the decomposition of the PVDF binder via a Fenton process prior to carbon flotation [110]. The amount of carbon black and PVDF effectively decreased by 50% in the LCO flotation concentrate [110]. Lastly, Kepler et al. proposed the recovery of carbon from the floating fraction of the dense media separation step by allowing the active material to deposit at the bottom of the vessel [111]. Although many organic and inorganic liquids or solutions could be used for this process, it could be challenging to find those that are non-toxic and environmentally friendly.

## 5. Hydrometallurgical Approach

Hydrometallurgical processes are used to extract and recover valuable metals from minerals or inorganic compounds using water as solvent [112]. This method is of great interest for battery recycling research because it is known to be low cost, involves little energy consumption, and exhibits good environmental footprint [10]. However, such advantages may be argued depending on flowsheet complexity, reagent schemes, effluent toxicity, and water consumption. Several review papers have described hydrometallurgical recycling processes [4,6,9,11,17,40,42,52,67,113,114,115]. Most of these review papers covered only the recycling of transition metal oxide LIBs and rarely mentioned the recycling of LFP batteries except for the review published by Wang et al., which only described process related to LFP recycling [116]. In addition, other review papers described the industrial operations or general situation of the recycling industry [8,10,83,117,118]. Our paper offers an update on the hydrometallurgical recycling processes at different stages: Academic development stage, patented, or industrially in operation. We also propose an industrial application critical view of the processes in development. Lastly, we also include an in-depth review of LFP battery recycling.

This section is divided in sub-sections depending on the leaching process and leaching agent used. It includes leaching with H_2_SO_4_, HCl, HNO_3_, organic acids, and other mineral acids or alkaline leaching agents. For each section, the current literature for leaching processes and for leach solution purification or extraction techniques is summarized in Tables S1–S10 in the Supplementary Materials. Tables S1, S3, S5, S7, and S9 summarize the types of pretreatments performed prior to leaching, accepted battery chemistries, states of feed material, reducing agents, leaching conditions, including acid and reducer concentrations, temperature, leaching time and solid concentration, and also the recovery rates. Tables S2, S4, S6, S8, and S10 describe the solution purification processes, including the reagent schemes, final products obtained, recovery rates, and process specific additional information.

Hydrometallurgy is the main method for LIB recycling that could separate different valuable elements (namely Co, Ni, Mn, and Li) as single element compounds [114]. Most processes are selective for each battery chemistry; however, there is a significant number of processes that could be used for mixtures of Co-, Ni-, and Mn-based batteries and only few processes that could be used for all types of batteries, including Co-, Ni-, Mn-, and PO_4_^3−^-based batteries. 

Many variables influence the design of the hydrometallurgical flowsheet and the reagent scheme used. Of them, the extent and nature of the pretreatment are of great importance, as discussed previously in pretreatment section. 

The complexity of the process also depends on the production objective (degree of downcycling). While some processes aim to recover elements as saleable compounds, others generate high-purity precursors for battery manufacturing or even resynthesize active materials for manufacturing new LIBs.

### 5.1. Sulfate System

Leaching with H_2_SO_4_ is the most studied hydrometallurgical process and is covered by many patents. A summary of the leaching conditions reported in the literature is presented in Table S1. The general conclusions from the literature could be summarized as follows:

▪ The presence of a reducing agent is important for accelerating leaching kinetics;
▪ Of all reducing agents, H_2_O_2_ appears to be the most efficient, followed by glucose and sulfites;
▪ Metallic Cu (anode current collector) exhibited lower leaching efficiency followed by Li, Co, Ni, and Mn;
▪ Roasting or chemical dissolution of PVDF is not a prerequisite for efficient leaching.

H_2_SO_4_ leaching involves the following reaction with Co-, Ni-, and Mn-based active materials [114]:

(4) $$2LiMO_{2\left(s\right)}+3H_{2}SO_{4}\to 2MSO_{4\left(aq\right)}+Li_{2}SO_{4\left(aq\right)}+3H_{2}O+\frac{1}{2}O_{2\left(g\right)}$$

where *M* is Co, Ni, or Mn.

Although the patent developed by Gupta et al. [119] reported good leaching rates without the addition of reducing agents, reductive leaching achieves better extraction yields and reduces leaching time [120]. The most popular reducing agent is H_2_O_2_, which reacts with the active material as follows [114]:

(5) $$2LiMO_{2\left(s\right)}+3H_{2}SO_{4}+H_{2}O_{2\left(aq\right)}\to 2MSO_{4\left(aq\right)}+Li_{2}SO_{4\left(aq\right)}+4H_{2}O+O_{2\left(g\right)}$$

Conversely, the dissolution reaction between the LFP active material and H_2_SO_4_ is promoted by the addition of an oxidizing agent such as H_2_O_2_. The following reaction then occurs:

(6) $$2LiFePO_{4\left(s\right)}+H_{2}SO_{4}+H_{2}O_{2\left(aq\right)}\to 2FePO_{4\left(aq\right)}+Li_{2}SO_{4\left(aq\right)}+2H_{2}O$$

**Figure 8:**
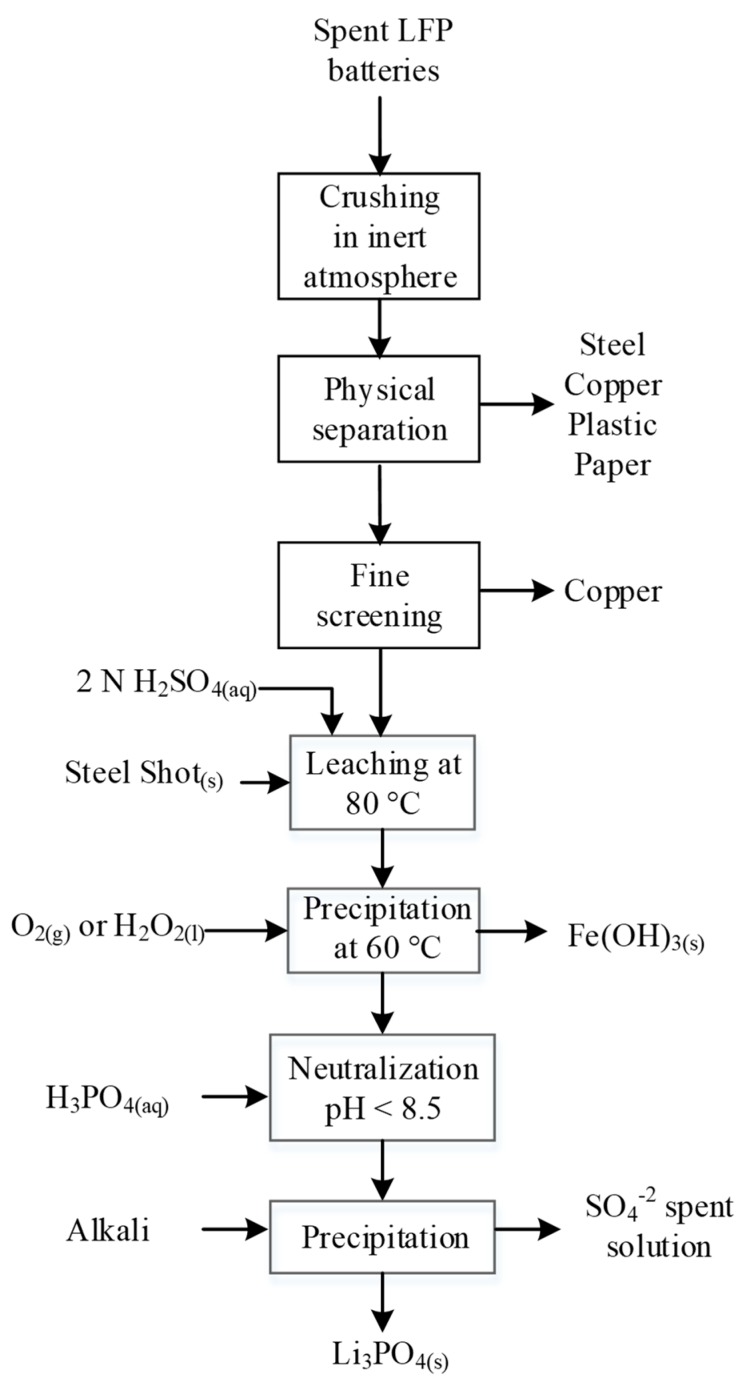
H_2_SO_4_ recycling process of lithium-iron phosphate (LFP) batteries patented by Recupyl (according to Ref. [46]).

H_2_SO_4_ is the most commonly used agent for the leaching or selective leaching of LFP, and the reaction described by Equation (6) is part of the process patented by Recupyl for LFP treatment shown in Figure 8. The patented process involves the primary crushing-screening step under inert gas. The undersize fraction is then suspended in water to liberate the active material. The resulting black mass is leached in H_2_SO_4_ in the presence of steel shots, which allow the recovery of residual Cu via cementation. The following steps involve the oxidation of Fe as hydroxides using H_2_O_2_ or O_2_ and the precipitation of Li_3_PO_4_ with H_3_PO_4_ and pH adjusting agents [46]. This simple process is robust against contamination from the anode and the Cu deposited on the cathode; however, it allows only the recovery of Li, while Fe and PO_4_^3−^ become waste products, not to mention the problems associated with the precipitation-solid/liquid separation of iron hydroxide [121].

Wu et al. [122] proposed a selective pre-leaching of LFP electrode in NaOH solution to dissolve Al current collector. The solubilized Al was recovered as Al(OH)_3_ via the addition of H_2_SO_4_. The black mass pre-leach residue was submitted to H_2_SO_4_ leaching at 60 °C with 30% H_2_O_2_. After 2 h, 82% of Li and 97% of Fe were solubilized, leaving a solid residue that contained PVDF and carbon. Then, Fe was precipitated out of the sulfate mother solution as iron oxide-hydroxide using NaOH—the same problem as in previous process flowsheet. Lastly, the well-known Li carbonate precipitation was performed using soda ash; sodium sulfate waste solution would also form [122]. 

When NH_4_OH was used to precipitate iron impurities, as proposed by Zheng et al., amorphous hydrated iron phosphate, FePO_4_·*x*H_2_O, was obtained [93], which could be restored as olivine FePO_4_ after carbothermal treatment. After Li_2_CO_3_ was recovered from the purified liquor, Zheng et al. resynthesized LFP-C from both precipitates via reducing sintering. The resulting active material provided the capacities of 152 and 138 mAh/g at 0.2 and 1 C, respectively [93].

**Figure 9:**
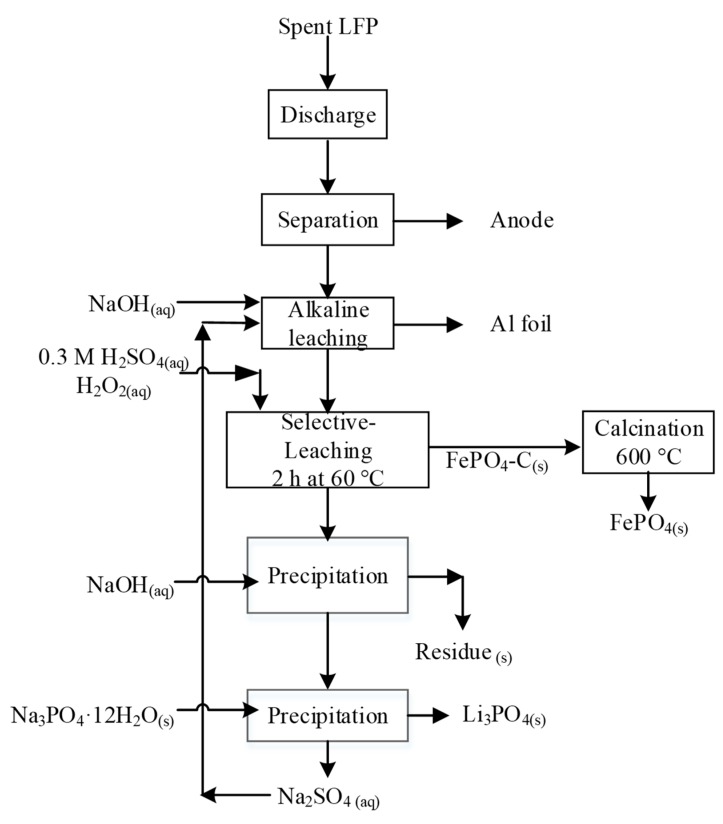
Selective leaching process proposed by Li et al. (according to [123]); here, LFP is LiFePO_4._

Although H_2_SO_4_ in conjunction with H_2_O_2_ easily dissolves LFP-C, Zou et al. claimed that the combination appeared much more selective in the presence of large fractions of acid and peroxide consumers, such as NCM, LCO, and LMO. While they suggested that high ionic Fe-O bonds energy impedes dissolution of LFP [43], we believe this is in contradiction with other publications demonstrating effective dissolution of LFP in H_2_SO_4_ [93,122]. Instead, low molar ratio of H_2_SO_4_ over LFP coupled with H_2_O_2_ proved high Li selectivity over FePO_4_ dissolution. This was demonstrated by Li et al. who performed selective leaching of LFP using H_2_SO_4_ and H_2_O_2_ under gentle conditions (0.3 M H_2_SO_4_, H_2_O_2_/Li molar ratio of 2:1, 60 *°*C, and leaching time of 100 min) [123]. This resulted in the complete delithiation of LFP-C, and hence, lithium brine and the precursor for the synthesis of LFP were recovered. The process is illustrated in Figure 9. Similar processes were patented by Umicore [124] and Rockwood Lithium [125]. For the process patented by Umicore, the addition of oxidant is controlled by controlling the redox potential of the leach solution to at least 200 mV vs. Ag/AgCl reference electrode [124]. This process is also suitable for simultaneously treating the anode, which is made of graphite or LTO, and cathode. The patent does not mention whether the leaching residue remains as orthorhombic FePO_4_ or other Fe-P rich phases. However, the harsh conditions prevailing in the reactor alter most probably the heterosite structure disabling any possibility to directly reuse it as cathodic material. In the process patented by Rockwood Lithium, Li is recovered from the mother liquor via electrodialysis or membrane separation [125].

**Figure 10:**
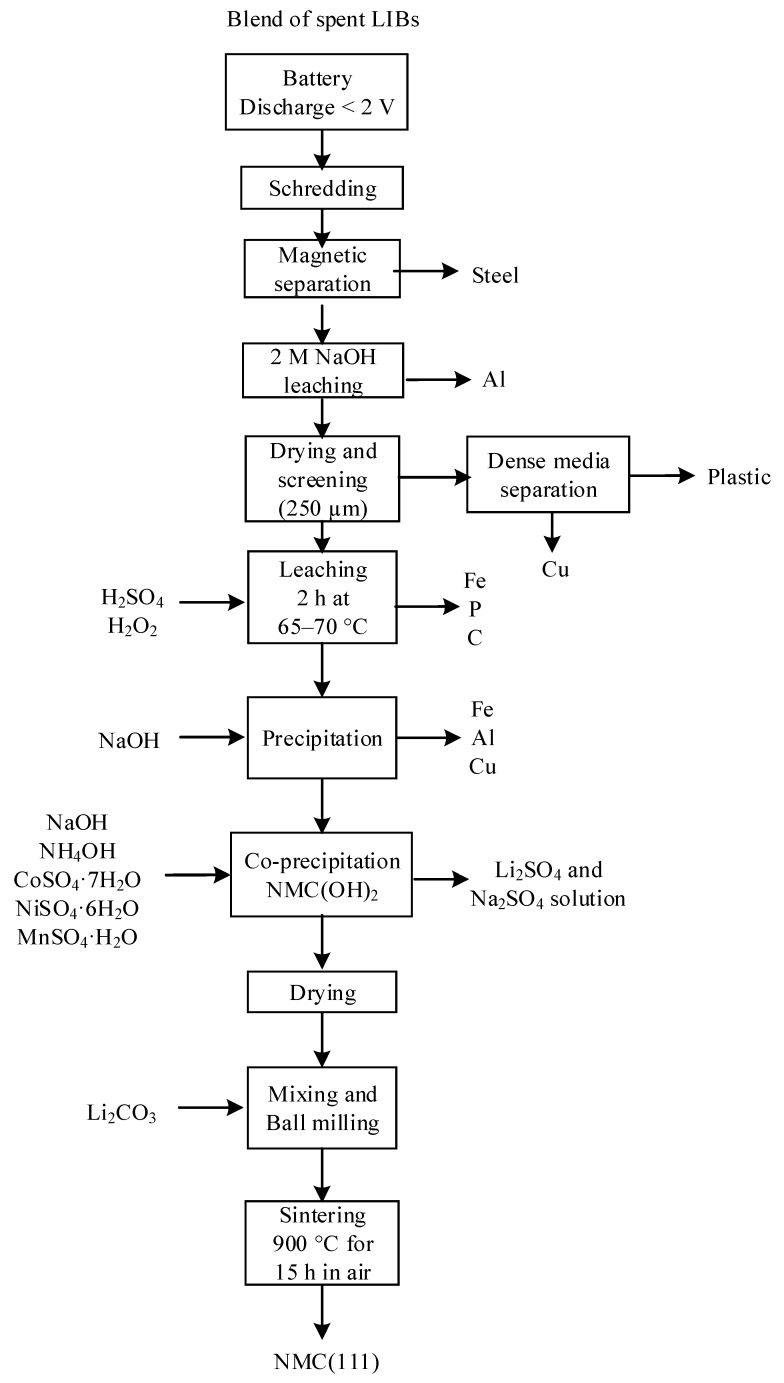
Selective leaching process proposed by Zheng et al. (according to Ref. [85,126]); here, NMC(OH)_2_ and NMC(111) are Ni_1/3_Mn_1/3_Co_1/3(_OH)_2_ and LiNi_1/3_Mn_1/3_Co_1/3_O_2_, respectively.

Starting from sulfuric acid leaching liquor obtained similar to Zou et al. [43], Gratz et al. [85] and Zheng et al. [126] applied purification process and element doping to obtain enhanced performance NMC (111). Zheng et al.’s process flowchart is shown in Figure 10. First, Fe, Al, and Cu were removed from the leaching brine that contained transition metals via precipitation with NaOH. After adjusting the Co:Ni:Mn molar ratio to be 1:1:1, Ni_1/3_Mn_1/3_Co_1/3_(OH)_2_ was co-precipitated using NaOH and NH_4_OH as alkaline/complexing agent for 48 h at pH 11 and a controlled temperature of 60 °C. Zheng et al. reported the results of four 7-day campaigns, where four 30 kg samples of blended spent LIBs with different proportions of LIBs types (LCO, NMC, LMO, LCO, LFP) were processed in a pilot-scale laboratory set-up [126]. The resulted solid Ni_1/3_Mn_1/3_Co_1/3_(OH)_2_ products served as precursor for resynthesizing NMC after mixing with 5% excess Li_2_CO_3_ and sintering at 450 °C and 900 °C. The obtained active material presented a d_50_ from 10 to 14 µm and an electrochemical capacity ranging from slightly over 140 mAh/g to 152 mAh/g at C/5 [126]. These publications are part of process development and commercialization of a LIB recycling pilot plant for Battery Resourcers Inc (Worcester, MA, USA). Although the idea of precipitating the NMC (111) precursors directly from the leach liquor is attractive, this method is commercially more sensitive to market changes, and therefore less flexible than the production of individual Co, Mn, and Ni salt concentrates intended for new active materials production.

Among other reducing agents for Co-, Ni-, and Mn-based batteries, Granata et al. and Chen et al. successfully used glucose, which reduced the active material following this reaction [127,128]:

(7) $$24\mathrm{LiCo}\left(\mathrm{III}\right)O_{2\left(s\right)}+36H_{2}{\mathrm{SO}}_{4\left(\mathrm{aq}\right)}+C_{6}H_{12}O_{6\left(\mathrm{aq}\right)}\to 24\mathrm{Co}\left(\mathrm{II}\right){\mathrm{SO}}_{4\left(\mathrm{aq}\right)}+12{\mathrm{Li}}_{2}{\mathrm{SO}}_{4\left(\mathrm{aq}\right)}+6{\mathrm{CO}}_{2\left(g\right)}+42H_{2}O$$

Takacova et al. compared the reaction kinetics of several LCO battery leaching conditions and concluded to a two-step leaching reaction that it is limited in the first 15 min by the cobalt reaction rate (*Ea* = 43–48 kJ/mol) [129]. In the second step, it is limited by a slow diffusion mechanism (*Ea* = 3–6 kJ/mol) dependent of acid concentration and temperature. Conversely, Meshram et al. came to the conclusion of a single-step surface layer diffusion controlled leaching mode, which does not fit with the shrinking core model [73]. However, the log rate empirical model used by Meshram et al. does not fit to linearity in the first 30 min, which confirms a change in the control mechanism. Takacova et al. suggested that lithium, primarily controlled by a mixed mechanism, diffuses first through the internal LCO structure, which opens the structure lattice, enhancing the leaching reaction between leaching agent and Co [129]. 

With the help of electrochemistry, Prabaharan et al. achieved 99% leaching rates for Co, Mn, and Ni from mixed spent batteries [130]. During sulfuric acid leaching, a direct current of 200 A/m^2^ was applied between the lead anode and the stainless -steel cathode. Although electrolysis improved leaching efficiencies for transition metals (above 99% after 3 h) when compared with sulfuric acid leaching without reducing agent (between 50% to 80% after 3 h) [130], it did not show any improvement when compared to chemical reducing agent such as H_2_O_2_ (close to 99% after 3 h) [131]. 

Apart from the references cited above, other research groups worked on various optimization of the sulfuric acid process by changing process parameters such as adding sonication to the leaching step [132,133], or replacing the reducing agent [134,135,136]. Other publications are summarized in Table S1 in Supplementary Information [137,138,139,140,141,142,143,144]. 

#### Solution Purification and Metal Extraction

Once cathodic material dissolution is completed, a series of purification steps may be used to purify the mother solution from its contaminants. Subsequently, the valuable metals can be extracted and made into saleable products. Table S2 in Supplementary Materials gives a summary of purification and extraction processes in the sulfate medium reported in literature. 

Impurities such as soluble Fe, Al, and Cu are generally precipitated first as hydroxides [72,127,145,146,147,148,149,150]. Recupyl recovered copper through cementation on steel shots [46], whereas Weng et al. suggested precipitating copper as CuS_(s)_ with Na_2_S [149]. Nan et al. extracted copper by solvent extraction with aldoxime-based extractant, which is very selective over cobalt and lithium [151]. 

Cobalt can also be precipitated as cobalt oxalate prior to purification using ammonium oxalate, as demonstrated by Nan et al. [151], or using oxalic acid as in Sohn et al. process [152]. However, to minimize coprecipitation of other elements, the deposition rate should not exceed 90%. Frequently, Co(II) is precipitated as carbonate, hydroxide, or oxalate from a solution purified from Fe(III), Al(III), and Cu(II). For manganese and nickel, precipitation as carbonate or hydroxide is usually performed after recovering cobalt. 

Solvent extraction can also be used to separate Co(II), Ni(II), Mn(II), and Li(I) from one another using PC-88A, Cyanex 272, or P507 [72,127,148,153,154,155]. Cyanex 272 is frequently used in mineral industry for separation of Co(II) from Ni(II) from sulfate solutions. However, Nguyen et al. reported some phase disengagement issues when used at high concentration [156]. Moreover, some co-extraction of lithium occurred (less than 20%), requiring a loaded organic scrubbing stage with Na_2_CO_3_ solution [156]. Alternatively, D2EHPA can be employed to extract Co(II), Ni(II), and Mn(II) together at pH 3.5 with 6 min contact time, leaving lithium sulfate in the aqueous solution [79,150]. In their review paper, Nguyen et al. proposed two flowcharts using solvent extraction for the separation of various metals and impurities found in the leach solution [156]. As a first example, a mixture of 5-nonylsalicylaldoxime (Acorga M5640) and 2-ethylhexyl phosphoric acid mono-2-thylhexyl ester (Ionquest 801) allowed the extraction of Cu(II), Al(III), and Fe(III) from Co(II), Ni(II), and Li(I) [156]. Then, Co(II) could be separated from the remaining ions in solution with Cyanex 272, as described earlier. In another example involving a solution of Mn(II) and Co(II), Nguyen et al. suggested using a mixture of Cyanex 272 and PC88A in conjunction with ethylenediamine tetraacetic acid (EDTA) [156]. The latter chelating agent reduced significantly the extraction efficiency for Co(II), thus making possible the separation of Mn(II) from Co(II) [156]. In terms of technology for solvent extraction, supported liquid membrane (SLM) attracts some attention and was successfully employed by Swain et al. for the separation of Co(II) and Li(I) using a mixture of Cyanex 272 and DP-8R [157].

Coprecipitation methods of Co(II), Ni(II), and Mn(II) along with hydrothermal crystallization coupled with sintering and appropriate adjustments of concentrations of precursors can regenerate cathodic material (NMC) up to 95% of the initial capacity as proposed by Chung et al. and other authors [149,150,158,159].

Electrowinning is a frequently used technique for plating metallic form of cobalt and nickel or electrolytic manganese dioxide (EMD) [130,153,160,161]. For cobalt electroplating, current efficiencies of up to 96% were obtained in mild acidic media (pH = 2–6) at high temperatures (50–90 °C) and current densities of 200–250 A/m^2^. Because the standard reduction potential of cobalt (II) (−0.28 V vs. Normal Hydrogen Electrode (NHE)) is lower than the potential for evolution of hydrogen (0 V vs. NHE), a low pH will promote the evolution of H_2(g)_. To avoid this side reaction, the cathode material should also exhibit a high overpotential over hydrogen. Prabaharan et al. and Lupi et al. used stainless steel and aluminum, respectively, as cathode material [130,153], which provide a relatively high hydrogen overpotential, are made of low-cost material, and allow easy recovery of metallic Co deposit.

Finally, lithium is mostly recovered as Li_2_CO_3(s)_, except by Recupyl, who precipitated Li_3_PO_4(s)_ by adding H_3_PO_4_ [160], and Rockwood Lithium, who produced LiOH_(aq)_ from electrodialysis [145].

### 5.2. Chloride System

Hydrochloric acid is the first system studied by Zhang et al. for hydrometallurgical treatment of spent LIB recycling [42]. Frequently compared to H_2_SO_4_ [120,129], HCl leaching also dissolves spent cathodic material in an efficient manner (>95%). Chloride-based processes are summarized in Tables S3 and S4 in Supplementary Materials. The dissolution reaction proposed by Meshram et al. involves reduction of transition metals (designated as M(III) or M(II) in the reactions) as the cathodic reaction and evolution of O_2(g)_ as the anodic reaction [114].

(8) $$2\mathrm{LiM}\left(\mathrm{III}\right)O_{2\left(s\right)}+6{\mathrm{HCl}}_{\left(\mathrm{aq}\right)}\to 2M\left(\mathrm{II}\right){\mathrm{Cl}}_{2\left(\mathrm{aq}\right)}+2{\mathrm{LiCl}}_{\left(\mathrm{aq}\right)}+3H_{2}O+\frac{1}{2}O_{2\left(g\right)}$$

In contrast, other authors have proposed a modified reaction that includes the evolution of chlorine gas as anodic reaction [4,6].

(9) $$2\mathrm{LiM}\left(\mathrm{III}\right)O_{2\left(s\right)}+8{\mathrm{HCl}}_{\left(\mathrm{aq}\right)}\to 2M\left(\mathrm{II}\right){\mathrm{Cl}}_{2\left(\mathrm{aq}\right)}+2{\mathrm{LiCl}}_{\left(\mathrm{aq}\right)}+4H_{2}O+{\mathrm{Cl}}_{2\left(g\right)}$$

The first reaction is thermodynamically favored when comparing the standard potential of O_2(g)_ (1.23 V) versus Cl_2(g)_ 1.36 V). However, the slower kinetics of oxygen evolution can promote oxidation of chloride anions to chlorine. On the other hand, cobalt is frequently used as an oxygen reduction catalyst in fuel cells. Therefore, considering the proximity of electromotive forces (EMF) for both reactions, kinetic aspect, and effect of local concentration gradient, both reactions are most probably happening. Previous literature does not provide a clear answer to this. 

For Co-, Ni-, and Mn-based batteries, the leaching power of HCl is high enough; therefore, a reducing agent is not necessary. From the literature reviewed, only two authors had used H_2_O_2_ as a reducing agent [162,163]. The resulted Li and Co recoveries (83% for both) obtained by Shuva et al. [163] were even lower than those in other studies without a reducing agent (for example, almost 100% for Li and Co) [129]. Takacova et al. demonstrated that HCl leaching in the absence of H_2_O_2_ presents a two-step kinetic, controlled first by the chemical reaction rate of cobalt (*Ea* = 40–48 kJ/mol) followed by a mixed control process (*Ea* = 20–26 kJ/mol) [129]. The latter activation energy value is similar to that obtained for cobalt by Shuva et al. [163]. 

**Figure 11:**
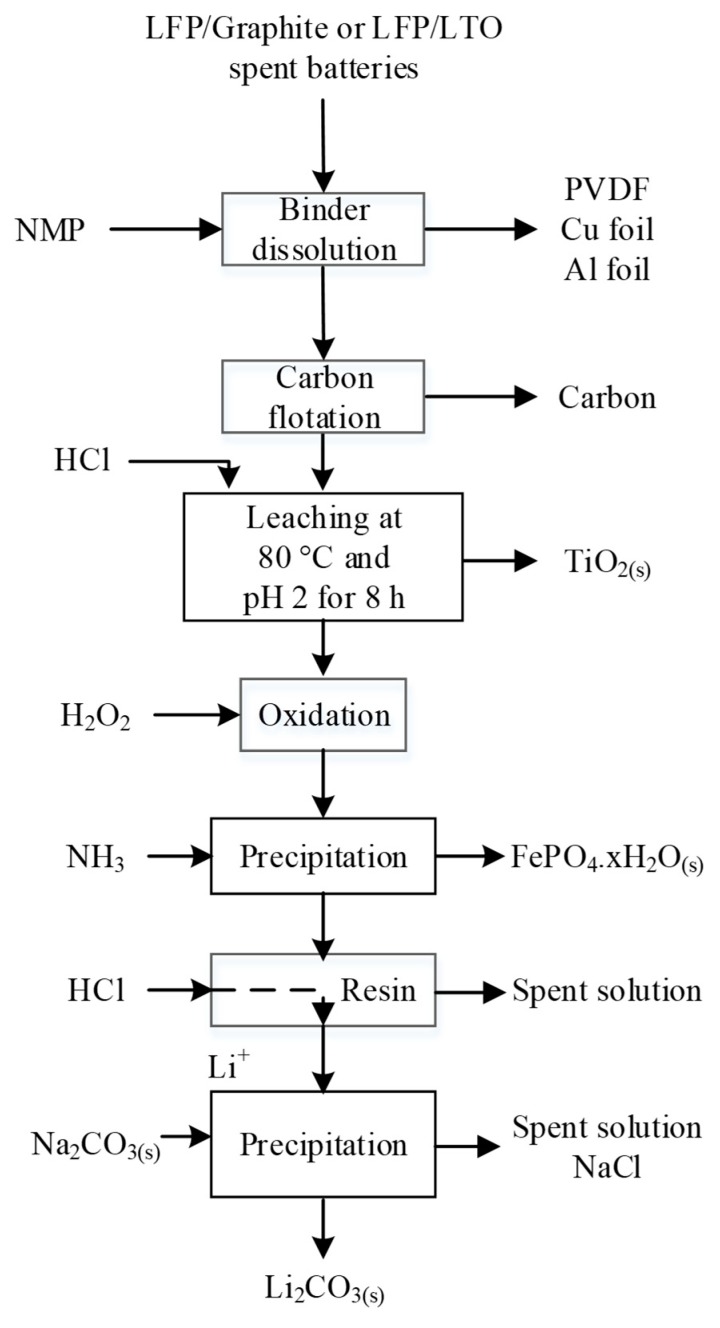
HCl leaching process proposed by Laucournet et al. (according to [164]); here LFP, LTO, and PVDF are for LiFePO_4_, Li_4_Ti_5_O_12_, and polyvinylidene fluoride, respectively.

Dissolution of LFP with HCl was demonstrated by Laucournet et al. [164] and Huang et al. [86]. The process was also patented by Umicore [165] and has the advantage of its ability to treat LTO anode simultaneously with LFP. During the leaching step, either the LFP is completely dissolved while titanium is recovered as TiO_2_(s) or lithium is selectively extracted from both spent active materials. In the first option, illustrated in Figure 11, Fe is precipitated as phosphate with or without a pre-oxidation step with H_2_O_2_. In the second option, the leaching residue containing Ti and Fe is leached a second time at pH = 2–3 to extract Fe. Solubilized Fe is recovered in the same way as in the first option. Furthermore, among various lithium recovery methods, Laucournet et al. proposed the use of cationic ion exchange resin [164]. As by Laucournet et al., both Kim et al. and Shin et al. recovered Fe as amorphous phosphate after hydrochloric acid leaching [166,167,168]. Then, the phosphate can be crystallized as strengite and used as a precursor for the LFP synthesis.

As in the sulfate system, recovery of valuable elements in the chloride system can be precipitated as hydroxides or carbonates [74,169]. Freitas et al. used electrodeposition and electrowinning as refining and extraction steps, respectively, at the end of which metallic cobalt was recovered [162]. Furthermore, organic extracting agent PC-88A offers very good selectivity for cobalt over lithium with a separation factor ($\beta_{\mathrm{Co}/\mathrm{Li}}$) of up to 1.3 × 10^5^ [42]. 

After lithium precipitation as Li_2_CO_3(s)_, the spent waste solution is generally composed of sodium chloride. At pilot or larger scale, this salt has to be crystallized to control sodium buildup [74]. Hence, one advantage of chloride leaching over sulfate leaching is the better market opportunity for the salt by-product resulting from the effluent water treatment. 

Other processes are summarized in Table S3 and Table S4 in Supplementary Information [170,171,172].

### 5.3. Nitrate System

Lithium and manganese were recovered from spent LIBs with nitric acid by Castillo et al. without the addition of a reducing agent [173]. In this process, cobalt, nickel, and iron remain in the solid residue, whereas manganese hydroxide is precipitated from the leaching solution with NaOH. The addition of a reducing agent helped to increase leaching of cobalt and nickel as demonstrated by Guan et al. and by Lee et al. [174,175]. As for other inorganic acids, H_2_O_2_ is the most popular reducing agent. Additionally, Guan et al. demonstrated the efficiency of galvanic reduction of transition metals using iron powder during a mechano-chemical leaching. However, this process suffers from the very low solid concentration (3 g solid/L) and the relatively slow kinetics compared with other processes. Furthermore, nitric acid seems to be inefficient for LFP dissolution without the addition of H_2_O_2_, as shown by Wu et al. and Yang et al. [77,122]. A summary of the nitric acid system is presented in Tables S5 and S6 in Supplementary Materials. The nitric acid leaching reaction proposed by Zeng et al. is as follows [17], but it may be questioned for its accuracy considering the oxidizing character of nitric acid.

(10) $$\mathrm{LiM}\left(\mathrm{III}\right)O_{2\left(s\right)}+3{\mathrm{HNO}}_{3\left(\mathrm{aq}\right)}\to M\left(\mathrm{II}\right){\left({\mathrm{NO}}_{3}\right)}_{2\left(\mathrm{aq}\right)}+{\mathrm{LiNO}}_{3\left(\mathrm{aq}\right)}+\frac{3}{2}H_{2}O+\frac{1}{4}O_{2\left(g\right)}$$

The recovery of valuable element(s) from nitrate solution is generally done by precipitation and/or coprecipitation processes [75,173,176]. More specifically, Moura et al. proposed the coprecipitation of precursors to synthesize CoFe_2_O_4(s)_ with ferric chloride, ammonium hydroxide, and ammonium acetate. The solid product required calcination at 450 °C to obtain Co doped ferrite. Finally, this product served as a catalyst for destroying methylene blue, which is a major contaminant in the textile industry effluent. Yang et al. obtained by precipitation a high performance NMC active material (239 mAh/g), but showing a low stability under cycling (81.2% capacity retention after 100 cycles) [177].

**Figure 12:**
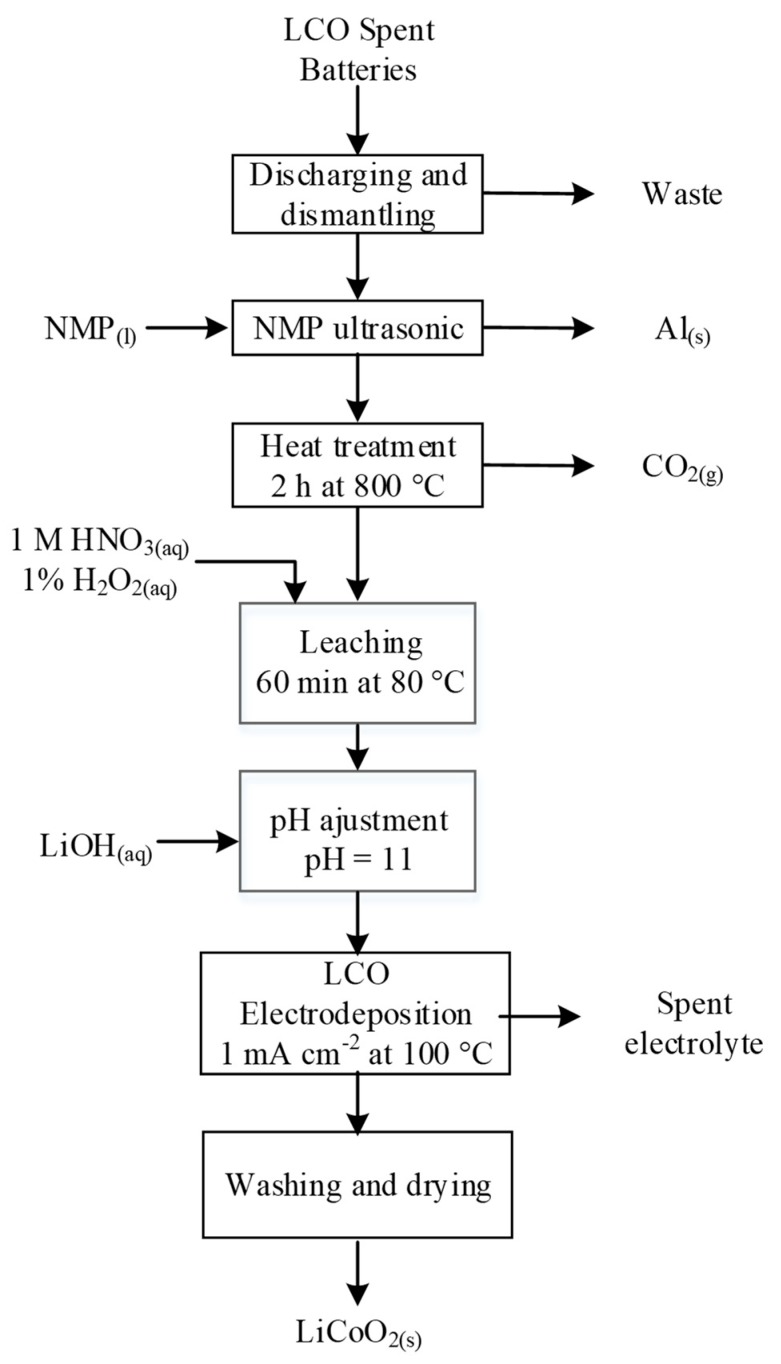
HNO_3_ leaching and electrodeposition process proposed by Li et al. (adapted from [180]); here, LCO and NMP are LiCoO_2_ and N-methyl-2-pyrrolidone, respectively.

Cobalt electrodeposition was successfully applied by Li et al. and Myoung et al. [178,179]. The former synthesized fresh LCO material from the nitrate leach solution alkalized to pH = 11 with 4 M LiOH by applying a current of 1 mA/cm^2^ between a Ni anode and a Pt cathode [180]. The process is summarized in Figure 12. The mechanism of the film formation proposed by Li et al. followed an electro-hydrothermal pathway. At high pH, the suspended Co(OH)_2(s)_ was solubilized as HCoO_2_^−^_(aq)_, which migrated to the positive electrode (nickel anode). In the vicinity of the anode, oxyhydroxide ions accumulated upon reaching the saturation concentration, thereby promoting precipitation of Co(OH)_2(s)_ on the Ni electrode. Application of a small current oxidized the divalent Co to trivalent form as a thin film of CoOOH_(aq)_, which is lithiated by following spontaneous reaction:

(11) $${\mathrm{Li}}_{\left(\mathrm{aq}\right)}^{+}+{\mathrm{CoOOH}}_{\left(s\right)}\to {\mathrm{LiCoO}}_{2\left(s\right)}+H_{\left(\mathrm{aq}\right)}^{+}$$

However, the resulting active material showed low energetic capacity, with a first discharge at 127 mAh/g at 0.1 C while the theoretical capacity of fresh LCO can reach 140 mAh/g [21]. 

### 5.4. Other Inorganic Acids or Alkaline Leaching Agents

Among alternative leaching agents, Meng et al. and Pinna et al. used phosphoric acid for calcined LCO with glucose and H_2_O_2_ as reducing agents [181,182]. The efficiencies achieved are close to 100% for both Li and Co. The cobalt in the solution was recovered as CoC_2_O_4(s)_ with oxalic acid. 

**Figure 13:**
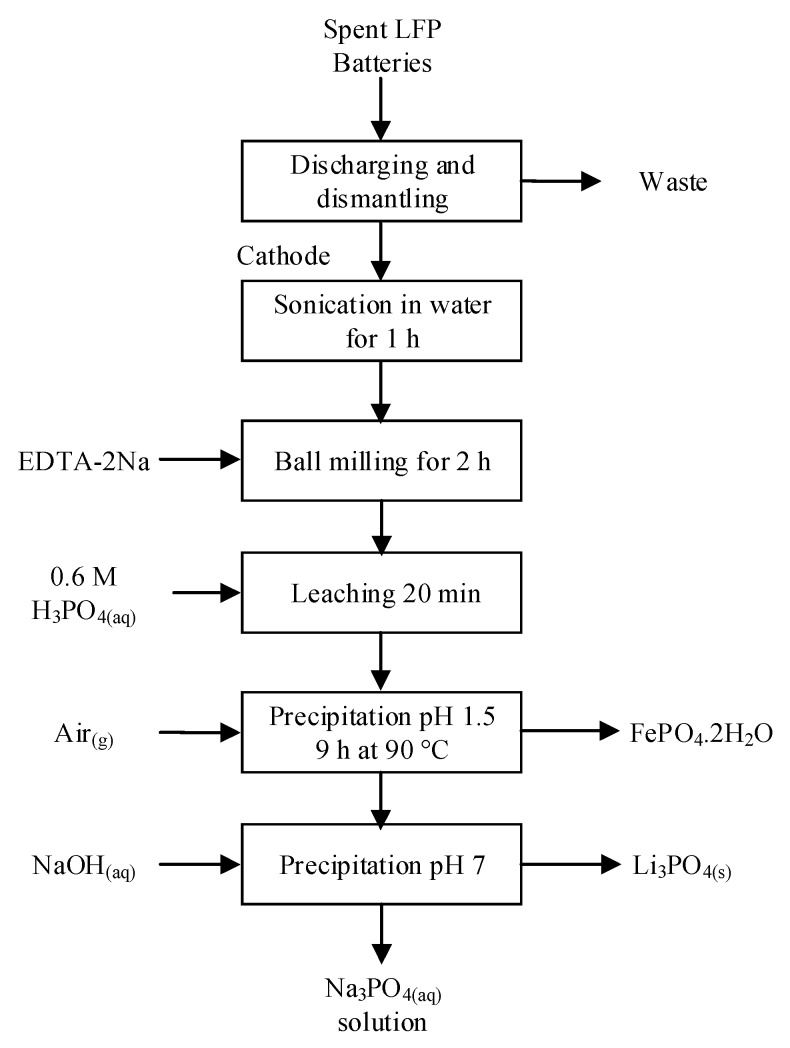
H_3_PO_4_ leaching process suggested by Yang et al. (according to [76]); here, LFP and EDTA-2Na are for LiFePO_4_ and ethylenediamine tetraacetic acid disodium salt, respectively.

However, phosphoric acid is also efficient to dissolve spent LFP even at low acid concentrations (0.5–0.6 M) and room temperature, as demonstrated by Bian et al. and Yang et al. [76,183]. Both authors recovered Fe(III)PO_4_·2H_2_O through reflux evaporation after leaching. In Bian et al.’s paper, there is no indication on the nature of the oxidizing agent; however, we can presume ferrous ions are oxidized to ferric with air during both leaching and reflux evaporation. The recovered precipitate was then subjected to the thermal relithiation process to resynthesize carbon-coated LFP. The discharge capacity of the active material thereof synthesized reached 159 mAh/g at 0.1 C and approximately 138 mAh/g at 1 C [183]. Yang et al. developed a mechano-chemical activation in the presence of ethylenediamine tetraacetic acid disodium salt (EDTA-2Na) prior to leaching to enhance the efficiency and kinetics. The process flowsheet is shown in Figure 13. In comparison with Bian et al.’s process, mechano-chemical activation reduced the leaching time by half and doubled the solid concentration (50 g/L) while maintaining similar extraction efficiencies. After leaching, hydrated FePO_4_ is precipitated through reflux evaporation while lithium is extracted as Li_3_PO_4_ with NaOH. [76] In both processes, although leaching conditions appear promising for larger scales, the evaporation step represents a major challenge for their commercial application because it consumes significant amounts of energy. 

Another inorganic acid tested in academic research is hydrofluoric acid (HF). According to Suarez et al., cobalt leaching efficiencies reached 98% with HF but did not achieve full leaching of lithium (80%) [184]. Considering the safety and environmental hazards related to HF and the poor performances obtained, this leaching agent is of low interest.

**Figure 14:**
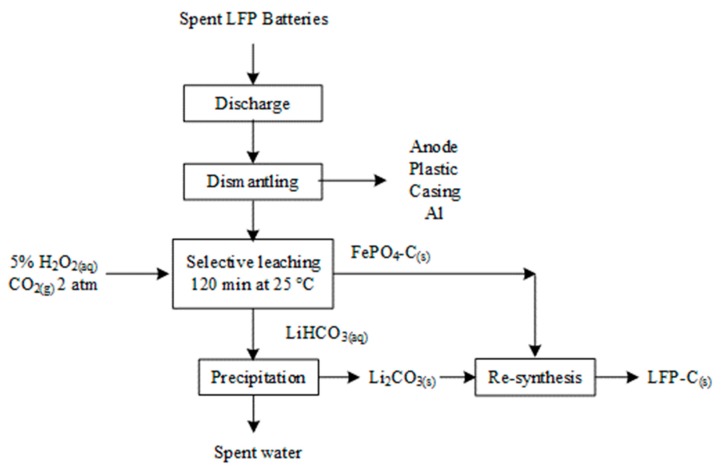
Selective leaching of LFP proposed by Amouzegar et al. (according to [185]); here, LFP is for LiFePO_4_.

Finally, selective leaching of lithium from LFP can be performed by contacting the cathodic material in a pressurized vessel with an oxidizing agent such as H_2_O_2_, O_3_, or O_2_ and CO_2_ to form a solution of LiHCO_3_ and a residue of FePO_4_. At a CO_2_ pressure of 2 atmosphere and room temperature, the Li extraction reached 100% in 30 min [185]. The low extraction rates of Fe and P at 0.5% and 3.5%, respectively, confirmed the high selectivity of the process. X-ray diffraction analysis performed on the solid leach product confirmed the FePO_4_ kept its orthorhombic structure, which could allow for reuse in new battery manufacturing. This process, illustrated in Figure 14, is patented by Hydro-Québec [185]. In another aspect of the invention, the LFP was contacted with Li_2_S_2_O_8_ in water to selectively extract Li ions as Li_2_SO_4_. In this case, the lithium persulfate acted as oxidizing agent and chelating agent.

A summary of leaching conditions and purification processes is provided in Tables S7 and S8 in Supplementary Materials in which other processes are summarized [186,187,188].

### 5.5. Organic Acid Leaching

Many organic compounds can serve as leaching agents. Organic acids generally offer good selectivity but are usually more expensive than inorganic acids. A summary of organic acid processes found in the literature is provided in Tables S9 and S10 in Supplementary Materials. Golmohammadzadeh et al. provided an exhaustive list of organic leaching agents and a detailed analysis of mechanisms controlling leaching reactions [9]. Based on the comparison of organic and inorganic leaching agents provided in this publication, we perceived the selectivity of several organic acids, their biodegradability, and weaker material corrosion as the most important advantages of organic acid leaching. 

Citric acid remains the most studied among the wide range of organic acids tested by various research groups. According to Li et al. and Chen et al., along with a reducing agent such as H_2_O_2_, citric acid solubilized efficiently (95% to 100%) all valuable metals from spent LIBs [104,189]. According to Li et al. and Golmohammadzadeh et al., at high temperature, the reaction with citric acid was chemically controlled for Li, Co, Ni, and Mn [104,190]. However, kinetics curves from the shrinking core model shown by Li et al. deviated from linearity during the first 4 to 8 min for Co, Ni, and Mn between 50 °C and 80 °C. The reaction could instead follow a two-step mechanism for transition metals. Interestingly, Chen et al. replaced H_2_O_2_ by green tea waste, reaching similar leaching efficiencies for both reducing agents (from 96% to 99%). Although the residue of green tea provided attractive leaching results and allowed reclamation of an industrial waste, this process may be difficult to apply at a larger scale due to difficulties related to reducing agent dosage. The authors did not provide a method to quantify the reductive power of the green tea waste, although they suggested a mechanism for the oxidation reaction.

High leaching efficiencies of Co and Li were obtained by Zheng et al. with formic acid and H_2_O_2_ [191]. Actually, close to 100% of both Li and Co were solubilized in less than 20 min at 60 °C. The following reactions for formic acid leaching were proposed by Zheng et al. [191].

(12) $$2\mathrm{LiM}\left(\mathrm{III}\right)O_{2\left(s\right)}+6{\mathrm{HCOOH}}_{\left(\mathrm{aq}\right)}+H_{2}O_{2\left(\mathrm{aq}\right)}\to 2M\left(\mathrm{II}\right){\left(\mathrm{COOH}\right)}_{2\left(\mathrm{aq}\right)}+2{\mathrm{LiCOOH}}_{\left(\mathrm{aq}\right)}+4H_{2}O+2O_{2\left(g\right)}$$

(13) $$4{\mathrm{HCOOH}}_{\left(\mathrm{aq}\right)}+2M\left(\mathrm{II}\right)O_{\left(\mathrm{aq}\right)}\to 2M\left(\mathrm{II}\right){\left(\mathrm{COOH}\right)}_{2\left(\mathrm{aq}\right)}+2H_{2}O_{\left(l\right)}$$

**Figure 15:**
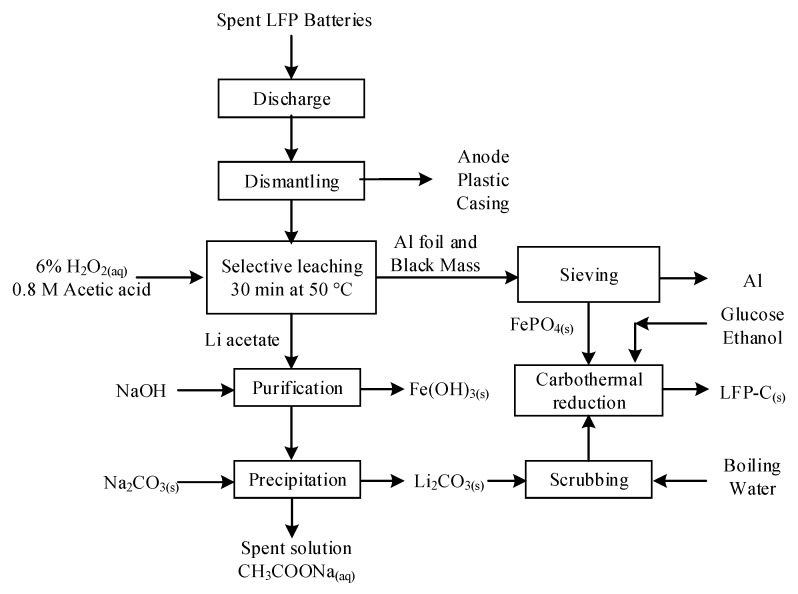
Selective leaching of LFP proposed by Yang et al. (according to [77]).

Cobalt, nickel, and manganese formates are soluble in water [192]. However, during leaching, these compounds form an amorphous precipitate over time as reported by Gao et al. [193]; a phenomenon that we associate with common ion-effect because of the presence of highly soluble lithium formate (346.6 g/100 g water at 100 °C) [194]. This precipitation behavior is confirmed by the rapid decrease in dissolution efficiencies for Co, Ni, and Mn salt after 10 min of leaching. Lithium formate did not seem to follow this precipitation process keeping a positive reaction rate all along the process. Offering a high solubility regarding Li ions (186 g/100 g water at 100 °C) [192], acetic acid is another potential leaching agent for NMC dissolution. Its low leaching efficiency for aluminum is an advantage over inorganic acids, allowing to selectively leach active material from cathode scrap [195]. Similarly, Yang et al. added acetic acid and H_2_O_2_ to an open reactor to selectively recover lithium from LFP batteries [77]. The lithium recovery reached more than 90% while phosphate and Fe dissolutions remained below 1%. The process flowchart is shown in Figure 15. Apart from processes discussed herein, other ones are summarized in Table S9 and Table S10 in supplementary Information [196,197,198,199,200,201,202,203,204,205].

Precipitation of cobalt salt took place during leaching with oxalic acid, as reported by Zeng et al. [206]. They reported recovery efficiencies of Li as soluble lithium oxalate and of Co as solid cobalt oxalate of 98% and 97%, respectively. In this process, oxalic acid acted as a leaching and reducing agent through evolution of CO_2(g)_. The main reaction proposed by Zeng et al. is as follows:

(14) $$2\mathrm{LiM}\left(\mathrm{III}\right)O_{2\left(s\right)}+4H_{2}C_{2}O_{4\left(\mathrm{aq}\right)}\to 2M\left(\mathrm{II}\right){\left(C_{2}O_{4}\right)}_{\left(s\right)}+2{\mathrm{LiHC}}_{2}O_{4}{}_{\left(\mathrm{aq}\right)}+4H_{2}O+2{\mathrm{CO}}_{2\left(g\right)}$$

**Figure 16:**
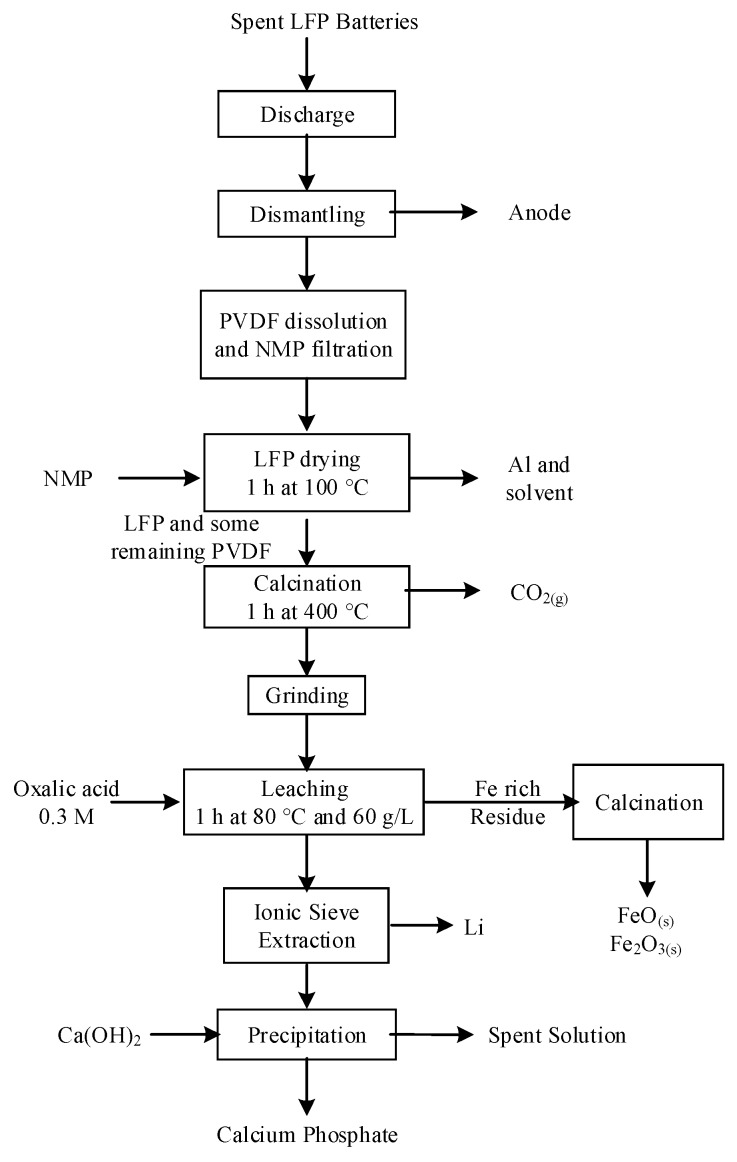
LFP dissolution process proposed by Li et al. (according to [207]); here, LFP, PVDF, and NMP are LiFePO_4_, polyvinylidene fluoride, and N-methyl-2-pyrrolidone, respectively.

Oxalic acid was also efficient for lixiviation of calcined LFP, as demonstrated by Li et al. in 2018 [207]. During this process, illustrated in Figure 16, the calcined LFP was exposed to 0.3 M oxalic acid at 80 °C. While lithium and phosphate are solubilized, the iron precipitated as iron oxalate and iron oxide. The iron-rich residue was recovered through filtration and Li was separated from the phosphate solution using an ionic sieve.

As alternative to inorganic acids, tartaric acid along with H_2_O_2_ gave the most promising results. Impressively, approximately 99% of Li, Co, Ni, and Mn were solubilized after 30 min at 70 °C [208]. The main reaction proposed by He et al. is:

(15) $$2\mathrm{LiM}\left(\mathrm{III}\right)O_{2\left(s\right)}+3C_{4}H_{6}O_{6\left(\mathrm{aq}\right)}+H_{2}O_{2\left(\mathrm{aq}\right)}\to 2M\left(\mathrm{II}\right){\left(C_{4}H_{4}O_{6}\right)}_{\left(\mathrm{aq}\right)}+{\mathrm{Li}}_{2}C_{4}H_{4}{O_{6}}_{\left(\mathrm{aq}\right)}+4H_{2}O+O_{2\left(g\right)}$$

Activation energies determined by kinetics studies indicated that the process, like most other organic leaching agents, is likely controlled by surface chemical reaction [208]. A summary of purification techniques is provided in Table S10.

## 6. Direct Recycling Approach

Instead of dissolving the active material completely, attempts can be made to refresh or reactivate it to recover any capacity or properties lost during cycling. This is referred as direct recycling. It is claimed to be less costly than leaching because intervention on the active material is minimized. One may argue that full refunctionalization needs at least the same number of steps as the leaching/resynthesis process and, thus, may not represent an economic advantage.

Different methods have been developed with varying degrees of success. The methods may involve physical separation of active material from other components, washing of PVDF binder, thermal treatment, lithium replenishment of the active material, and final thermal treatment step. Table S11 in Supplementary Information summarizes the process and reagent schemes, specifies the types of batteries treated, and gives values of key process parameters. Where available, the capacity of refreshed material is also provided. Note that parameters of cycling and, more particularly, details of the protocol followed for button cell fabrication are rarely specified in publications despite their high influence on the capacity of cells. Therefore, comparison with each process becomes difficult.

A simple process patented by Toyota [209] claims to clean out adsorbed phosphorous compounds from the surface of the cathode active material. These compounds are coming from the dissociation of electrolyte (LiPF_6_) during cycling. According to the authors, applying very high frequency ultrasound (>900 kHz) for up to 30 min on the whole undamaged battery, the thin film covering the positive electrode was stripped off. The battery treated thereby was tested for internal resistance and then classified according to a threshold for reuse in electric vehicles, in stationary purpose, or for definitive discard. It is well known that internal resistance is not the only indication of cells degradation [210] and such simple process would not be efficient to completely refurbish cathodic active material, clean the oxidized current collectors, and to regenerate un-cyclable lithium fixed in the anodic material.

Farasis Energy, Inc. developed a selective leaching process during which the shredded cathode is cleaned off residual Cu and Al in the alkaline solution after separation from other components [107]. The solution is made of a complexing agent (5 M NH_4_OH) and 1 M LiOH with O_2(g)_ bubbling to promote oxidation of metallic aluminum and copper. After 12 h, these contaminants are completely solubilized. The patent claims to treat any type of LIB. However, for LFP batteries, pH shall be maintained in a closed region to minimize delithiation of the active material by oxygen while avoiding phase transformation of LFP to iron oxide at pH above 14 [211].

Other authors developed thermal refunctionalization processes, during which the active material was calcined with or without a lithium precursor. Wei et al. proposed a process of thermally reactivating the transition metal oxides after selective leaching of the aluminum current collector with NaOH [88]. The recovered oxides are suitable for metal air battery. Similarly, Nihon Kagaku Sangyo Co. Ltd. (Tokyo, Japan) patented a process that selectively leaches contaminants, namely, lithium compounds, adsorbed to the surface of active material using ammonia water [212]. The refreshed active material was then thermally treated at 600–700 °C. 

**Figure 17:**
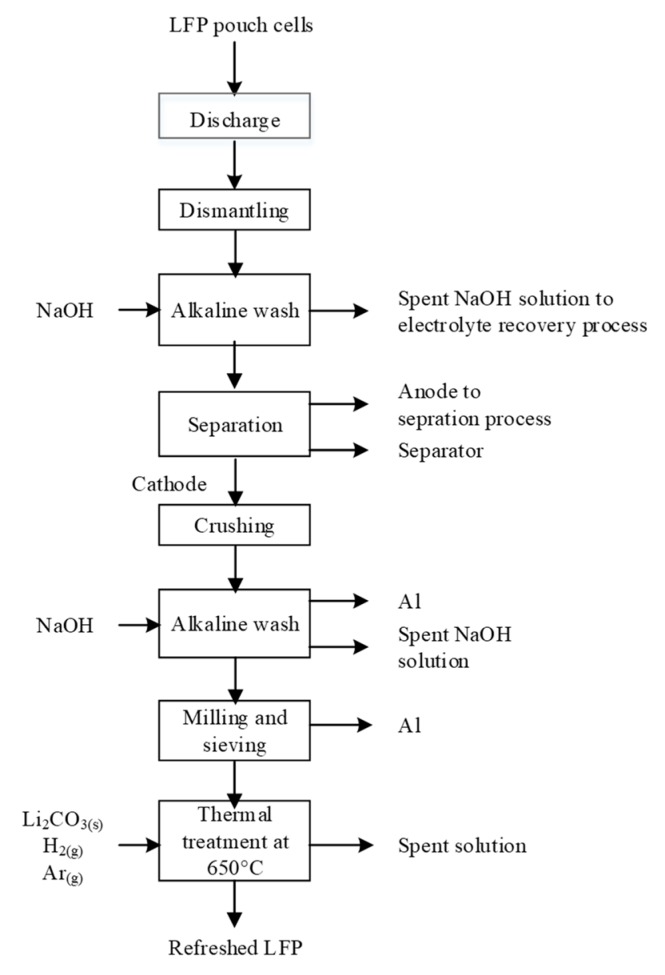
Pilot-scale thermal regeneration process of LFP (according to [97]).

Similar thermal reactivation processes were explored for LFP batteries, notably by Kim et al. [69]. In the process of Kim et al., after opening of batteries and sorting their components, the cathode was submitted to thermal treatment at 500 °C under nitrogen flow. This process decomposed the PVDF binder and liberated the active LFP from other components. Nevertheless, the recovered cathodic material exhibits low electrochemical capacity, reaching 136 mAh/g at 0.1 C (Refers to charging or discharging current rate. 1 C corresponds to full charge or discharge in 1 h.) versus Li^0^ [69]. This process was improved by Chen et al. [213] and Li et al. [97] at pilot scale by introducing a lithium precursor and a reducing gas during the thermal treatment. In this way, the depleted cathodic material was replenished with lithium. This fully lithiated LFP exhibited electrochemical capacity of 145 mAh/g at 0.1 C versus Li^0^. The complete process flow chart is shown in Figure 17. Similarly, Wang et al. developed a process integrating a cathode washing step with DMC for the removal of organic compounds before component separation, milling, and thermal treatment. This last step was performed at two different temperatures, namely, 200 °C and 500 °C under nitrogen atmosphere. It allowed carbonization of PVDF, refreshing the carbon layer around the LFP particles. Regenerated LFP exhibited good capacity (150 mAh/g at 0.1 C) even at a high discharge rate (100 mAh/g at 20 C) but showed a stability slightly lower than expected from LFP (95% capacity retention after 100 cycles). In addition, the chemical assay of regenerated LFP showed a contamination of aluminum at 0.5%. Although it did not seem to affect the capacity of refreshed material, the presence of this contamination has to be minimized to avoid their accumulation in the recycled active material. 

The process patented by Retriev [214] included crushing and screening steps followed by thermal treatment at 500 °C to modify the carbon surface and destroy PVDF. Then, selective flotation was applied to remove carbon. The sink fraction was mixed with appropriate amounts of lithium hydroxide solution before calcination at 500–800 °C. The capacity recovery was around 95%. This process is essentially similar to the one developed by Poe et al. [108], except that PVDF was removed through NMP washing instead of being thermally calcined. The final calcination-relithiation step was also performed at higher temperatures (850 °C) with Li_2_CO_3(s)_. 

**Figure 18:**
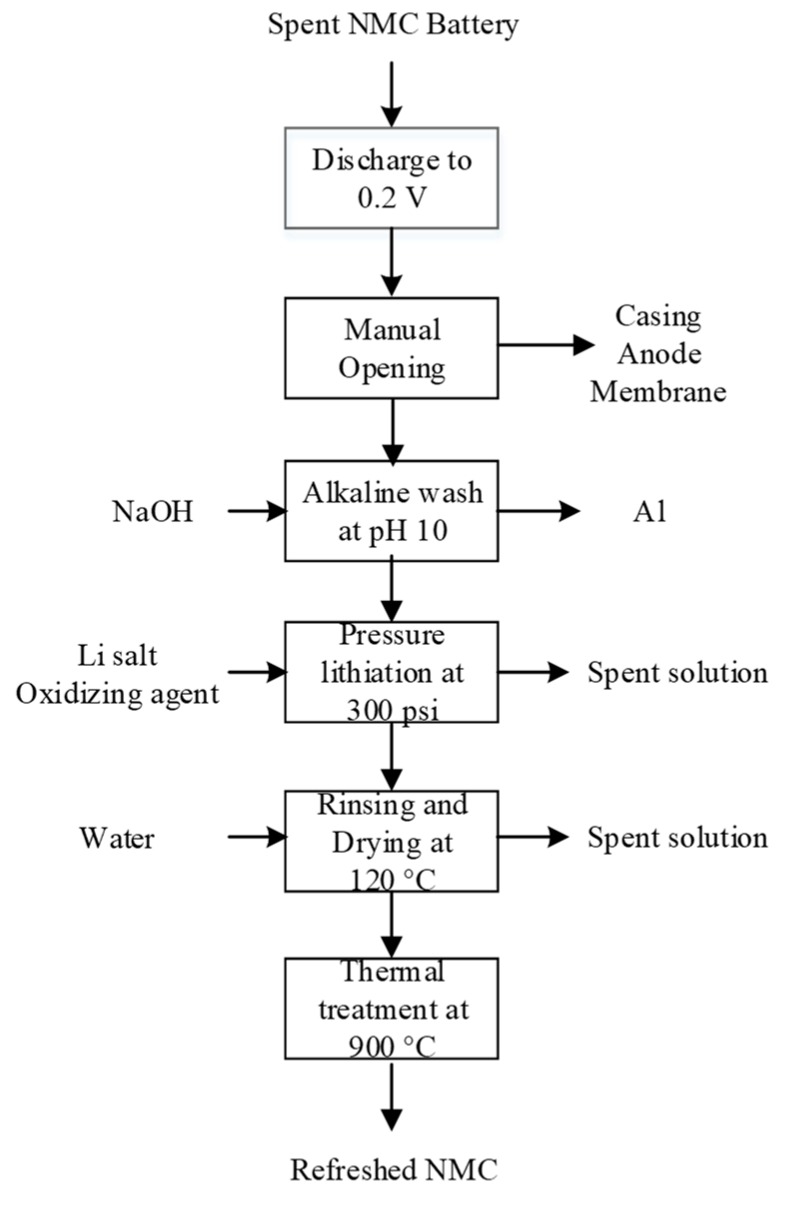
Hydrothermal process proposed by Sloop (according to [218]).

Hydrothermal relithiation was proposed by Sloop. [215] and by Shi et al. [216] after the spent batteries were dismantled, screened, and PVDF was removed with solvent. The former used 24% LiOH solution and the latter preferred a blend of 1 M LiOH and 1.5 M Li_2_SO_4_ as the lithium precursor. Although the optimal process temperatures were similar in both studies, the 12 h duration claimed by Sloop was significantly longer than the 4 h one proposed by Shi et al. Both processes ended with final annealing, at a temperature slightly higher in Shi et al.’s study (800 °C). Sloop’s process flowchart is shown in Figure 18.

**Figure 19:**
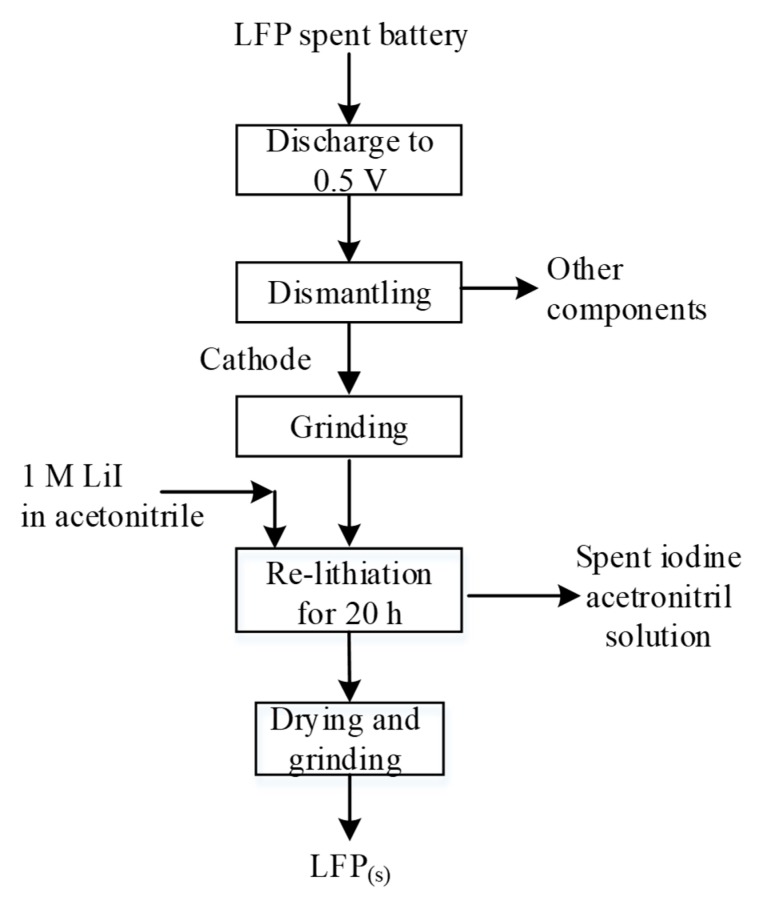
Iodide relithiation process proposed by Ganter et al. (according to [217]).

Regenerated LFP with good electrochemical capacity (150 mAh/g) was obtained by Ganter et al. after chemical relithiation in solvent solution of lithium iodide (LiI) [217]. First, as illustrated in Figure 19, the spent LFP batteries were prepared by discharging them to 0.5 V, followed by dismantling and isolating the cathode. The black mass was then manually scraped off the cathodic current collector and ground. The following relithiation step involved suspending spent LFP in acetonitrile and 1 M LiI for approximately 20 h. A similar process with slight variation on preparation steps and iodide solvent was patented by Sloop in 2016 [58]. However, large-scale utilization of iodide and organic solvents can cause severe environmental and safety issues, which make both processes not really commercially applicable.

**Figure 20:**
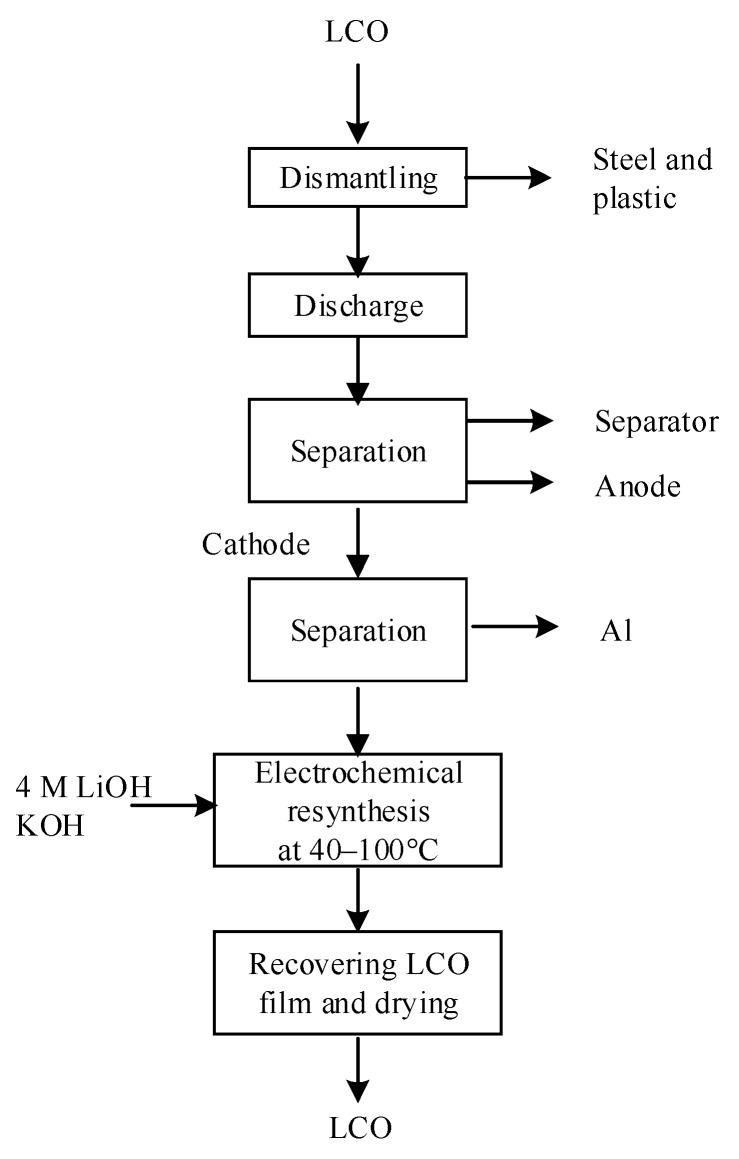
Electrochemical resynthesis process for LCO (according to [219]).

Electrochemical relithiation has been studied by several authors [219,220,221]. Ra et al. proposed an electrochemical dissolution/deposition process for LCO batteries. This process, illustrated in Figure 20, involved the application of a small galvanostatic current (1 mA/cm^2^) in an aqueous slurry of spent LCO powder with 4 M LiOH and KOH at a temperature of 40–100 °C. During the process, LiCo(III)O_2(s)_ dissolved as cobaltous oxyhydroxide (HCo(II)O_2_^−^) and oxocobaltic (Co(III)O_2_^−^) ions in the presence of OH^-^, which migrated towards the platinum working electrode (anode). Both Co complexes are oxidized in the presence of OH^−^ and Li^+^. Then, they created a thin film of LiCoO_2_(s) on the electrode. This principle was evidenced by Li et al. following slightly different reactions [180]. According to Li et al., the cobalt oxyhydroxide ion (HCo(II)O_2_^−^) oxidized to solid oxyhydroxide (Co(III)OOH_(s)_). Then, a chemical transformation reaction formed LiCo(III)O_2(s)_. In both cases, the capacity of the refunctionalized material was close to the practical capacity of LCO (140 mAh/g [21]), at approximately 130 mAh/g and 135 mAh/g, respectively, for Li and Ra [180,219]. Direct current electrochemical relithiation could also serve to regenerate spent LFP, as proven by Yang et al. [222], Ganter et al. [217], and Wang et al. [223]. Yang et al. and Ganter et al. followed similar relithiation methods by discharging an LFP cell versus lithium metal negative electrode. However, the cathode preparation procedure diverged. While Yang et al. liberated the active material from the current collector and the binder, Ganter et al. coupled directly spent cathode with the lithium metal negative electrode. The initial capacities obtained from the first two studies reached 150 mAh/g and 140 mAh/g, respectively. Unfortunately, this simple relithiation process is difficult to scale up, as pointed out by Ganter et al. [217]. It did not repair the structural defect, nor did it remove contamination such as copper, which may plate on the positive electrode during cell over-discharge [71]. 

A pulsating voltage method for relithiation was developed by Zhu et al. [220]. The spent cobalt oxide was exposed to a very short (500 ns) high-voltage pulse at a frequency of 150 Hz in a 0.1 M LiOH aqueous solution. Upon pulsed electrical discharge, the suspended LCO underwent a structural change, which fixed cation disordering accumulated during cycling and supposedly improved Li^+^ intercalation properties. The resulting LCO did not reach a satisfactory electrochemical discharge capacity, at 127 mAh/g at 0.2 C [220]. 

Many other processes not discussed herein are summarized in Table S11 in Supplementary Information [224,225,226,227,228]

## 7. Challenges and Trends for Recycling

Although LIBs are promoted by governments all around the world, their image of a green solution against global warming has been questioned in both technical and non-technical media. Undeniably, many publications, some of questionable quality, depicted LIBs as a global environmental issue and being an important source of pollution, with a resulting negative environmental impact. While it is true that environmental footprint of LIB manufacturing is significant, many scientific studies demonstrated the benefit of LIBs [38,229,230,231]. Moreover, recycling of spent LIBs is an inherent and imperative solution to reduce this environmental footprint. Over the past decade, the lithium battery industry and governments have made significant efforts to implement and develop recycling operations. Circular economy is now the subject of numerous works and events. Recently, two major international events were dedicated to this aspect applied to battery segment [232,233].

However, LIB recycling faces some challenges, which limit its scalability and fast development.

1. First, the collection of spent LIBs is costly and requires involvement of all members of the consumption chain, including producers, sellers, governments, waste managers, and consumers. For instance, in European Union, less than 20% of the spent LIBs were collected in 2016 according to Eucobat (European Compliance Organizations for Batteries) [16] despite well-established regulations and recycling industry. In North America, recovery estimation is around 5% [234]. Other than collection, shipment of spent LIBs, particularly large lithium-ion battery packs, is problematic. This critical aspect is one of the most discussed topics in conferences and workshops, as seen during NaatBat 2019 Battery Conference held in Buffalo. It is also studied by the United Nations Committee of Experts on the Transport of Dangerous Goods, and several sessions are dedicated to this subject.
2. Secondly, the diversity in LIB materials and rapid evolution of technologies make it difficult to plan the variation of material inflow. Over the past decade, cathode composition has faced a deep evolution from early stage of LCO to new composition such as NMC, LFP, NCA, and LMO. In addition, some products having cathode additives such as yttrium are now available on the market, which will eventually end up in recycling process. Moreover, modification of the anode material is undergoing, shifting from graphite to LTO, and, in the near future, to silicon-based materials. Further, the segment of electrolyte additives in lithium-ion batteries is a dynamic sector; we noted 312 patents on various additives in 24 months. Lastly, the current research push towards all-solid-state Li batteries is imposing new challenges necessitating early consideration of the end-of-life fate of these new devices to simplify recycling. Considering these upcoming changes, management of the mass balance, mitigation of health and safety issues (One of the example is propane sultone, which is adopted in some composition and has a safety issue to manage with accuracy (see CAS number 1120-71-4)), and waste management will continue to be a challenge for battery recycling plants.
3. Finally, strong competition between battery recyclers and the volatility of commodities market prices restrict profit margins for recyclers, which weaken the viability of the industry. This aspect must be addressed by reinforcement of producer responsibility to ensure the involvement of the producer of the waste in the recycling cost when recovered materials cannot compensate the processing cost, e.g., manufacturers of LFP and LMO systems. This concept is already largely accepted in Europe, where the recycling fee for alkaline batteries is shared by the manufacturer and importer.

Globally, all battery manufacturers as well as mobile phone/laptop/EV manufacturers and end users must work in concert for promoting a circular economy approach by enhancing closed-loop processes leading to regeneration of battery grade precursors or compounds with minimum environmental impact. The processes coupling mechanical and chemical approaches are the most suitable for closing the loop in regards to several materials [232,233]. 

## 8. Conclusions

The exponential growth of the LIB market has put pressure on environment and natural resources. Introducing secondary resources from recycling will reduce the environmental footprint of LIB manufacturing. The quantity of spent batteries available for recycling is increasing, especially with the arrival of the first generation of electric cars. However, significantly low collection rates have been achieved owing to safety issues, bad chain public information, deficient regulation/enforcement, low recycling process efficiencies, high transportation costs, and high recycling cost. Accordingly, actors in LIB supply chain have not yet adopted the circular economy principles. 

Most pyrometallurgical processes, which currently constitute the dominant recycling route, cannot recover lithium as a valuable element and are not effective for phosphate-based batteries. Moreover, the current business model for recycling of large battery packs is based on service fees to the battery owner. Are these fees discouraging the adoption of lithium-ion batteries? There is a need to find alternative, low-cost recycling processes for large battery packs from EVs and stationary systems. In this context, hydrometallurgy is an alternative process to the standard pyrometallurgical process; however, its large-scale implementation is still significantly limited: Recupyl being the only large-scale operator in this domain due to high operating cost. Still, several hydrometallurgical-based pilot projects are in development, particularly in North America. In this respect, sulfuric acid leaching is the dominant process and offers the advantage of low reagent cost and well-known chemical process characteristics. 

To increase the value chain of recycling products, significant efforts are devoted to developing a direct recycling approach, with Argonne Laboratory being one of the most active supporters of this route. In addition to shortening the chemical process path, direct recycling allows to process economically low-value battery chemistry, such as LFP, by generating added-value products. However, this technique is yet to prove full restoration of the initial cathode capacity. Nevertheless, recent progress in relithiation methods, particularly in electrochemistry, and mechanical separation of cathode materials (selective flotation, magnetic separation, and so on) show promise for improved efficiency. 

Apart from direct recycling and hydrometallurgical leaching, a new approach being developed is selective leaching, in which lithium is selectively extracted from the hosting material while retaining the internal structure of the cathode material. Combined with most recent developments in mechanical separation, this approach may have a considerable success for the treatment of mixed cathodes or LFP batteries. 

Issues related to process development, transportation logistics, safety issues, and integration of the supply chain are the most important constraints to the large-scale deployment of recycling. While some of the problems encountered by the industry can be solved through technology, deeper involvement of OEM and governments through more severe regulation is mandatory to progress rapidly and reach satisfactory recycling rates to tackle the anticipated surge in volumes of spent batteries from increasing numbers of EVs.

## Figures and Tables

**Table 1:** Electrochemical properties of six of the most important types of Li-ion batteries; here, LCO, NCO, NCA, NMC, LMO, and LFP-C are lithium-cobalt oxide, lithium-nickel-cobalt oxide, lithium-nickel-aluminum oxide, lithium-nickel-manganese-cobalt oxide, lithium-manganese-oxide, and carbon-coated lithium-iron-phosphate, respectively.

Cathode Material	LCO	NCO	NCA	NMC	LMO	LFP-C
References	[21]	[21]	[22,23]	[21,24]	[21,25]	[21]
Average potential (V vs. Li^0^)	3.7–3.9	3.8	3.8	3.3	3.8	3.3
First cycle discharge capacity (mAh/g at 0.1 C)	140	180	180–200	170	120	155–160
Specific energy (Wh/kg)	520	675	680–760	560	455	560
Capacity retention after 100 cycles (% of initial capacity)	97–98	N/A	93	95	89–93	>99

**Table 2:** Variation of estimated global warming potential (GWP) of lithium-iron-phosphate synthesis and cradle-to-gate energy consumption calculated for 1 kg active material production from data provided in literature; here, SS, HT, and N.S. are solid state, hydrothermal, and not specified, respectively.

Synthesis Method	SS	SS	HT	HT	SS
References	[41]	[39]	[37]	[38]	[38]
GWP (kg CO_2_ equivalent)	4.2	2.0	5.3	4.5	3.0
Energy consumption (MJ)	N.S.	19	N.S.	56	34

**Table 3:** List of current recycling facilities and recycling processes they use.

Company (Location)	Process Type	Recovered Elements
Retriev (Canada)	Cryo-hydrometallurgy	Li
Umicore (Belgium)	Pyro-hydrometallurgy	Co, Ni, and Mn
Sumimoto (Japan)	Pyro-hydrometallurgy	Cu, and Ni
Recupyl (France)	Hydrometallurgy	Li, Co, Ni, Mn, Cu, and Al
Snam (France)	Pyrometallurgy	Cd, Ni, Co, and Fe
Accurec (Germany)	Pyrometallurgy	Al, Cu, Co, Ni, and Fe
Glencore (Canada)	Pyro-hydrometallurgy	Ni, and Co
Batrec (Switzerland)	Pyro-hydrometallurgy	Zn, Co, Ni, Mn, and Hg
AkkuSer Oy (Finland)	Mechanical	LIB active materials
OnTo Technology (USA)	Direct Recycling	LIB active materials
Dowa (Japan)	Pyrometallurgy	Co, Ni, and Mn
Nickelhütte Aue Gmbh (Germany)	Pyrometallurgy	Co, Ni, and Mn
Brunp (China)	Pyro- and hydrometallurgy	Li, Co, Ni, Mn, Cu, and Al

**Table 4:** List of current recycling operations: hydrometallurgical and direct recycling.

Company	Start Date	Method Used to Access the Active Materials	Method Used to Recover the Valuable Components	References
Recupyl (France)	February 1996	Dry mechanical treatment under inert atmosphere	H_2_SO_4_ leaching	[56,57]
			H_2_O_2_ reduction	
			Selective precipitation	
OnTo Technology (USA)	November 2005	Supercritical CO_2_	Heating at 400–900 °C, LiOH alkaline solution	[58,59]
	June 2010	Disassembly/cutting		
	November 2016	-		
Retriev (Canada)	December 2013	Wet mechanical treatment	Flotation and alkaline treatment	[60]
Brunp (China)	August 2018	Pyrolysis/hydrometallurgy	H_2_SO_4_ leaching	[61]
			H_2_O_2_, Na_2_S, or NaHS reduction	
			No indication of metal recovery in reports	

**Table 5:** Elemental composition of spent Li-ion battery (LIB) electrodes (wt.%) reported in the literature; here, LCO, NMC, LNO, and LFP are lithium-cobalt oxide, lithium-nickel-manganese-cobalt oxide, lithium-nickel oxide, and lithium-iron phosphate, respectively; and N.S. is not specified.

Description	Co	Li	Al	Cu	Fe	Mn	Ni	P	References
LCO—anode and cathode: Black mass with current collector contamination	26.77	3.34	5.95	1.24	3.76	1.1	0.34	N.S.	[72]
LCO, NMC, and LNO—cathode black mass: manually detached from current collectors	35.8	6.5	0.58	0.005	0.06	11.6	10.06	N.S.	[73]
Mixed LIBs—roasted black mass: Mechanically shredded and separated from current collectors	11.73	1.95	0.58	0.004	N.S.	8.48	0.26	N.S.	[74]
Mixed LIBs—cathode black mass: Manually detached from current collectors	34.66	5.92	0.68	N.S.	N.S.	11.26	0.68	N.S.	[75]
LFP—cathode black mass: Manually separated	N.S.	4.08	0.16	N.S.	31.25	N.S.	N.S.	18.94	[76]
LFP—entire cathode: Manually separated	N.S.	3.46	16.85	N.S.	26.45	N.S.	N.S.	15.88	[77]

## Supplementary Materials

The following are available online at https://www.mdpi.com/1996-1944/13/3/801/s1, Table S1: Summary of leaching processes with sulfuric acid, Table S2: Summary of purification and extraction processes in sulfate medium, Table S3: Summary of leaching processes with hydrochloric acid, Table S4: Summary of purification and extraction processes in chloride medium, Table S5: Summary of leaching processes with nitric acid, Table S6: Summary of purification and extraction processes in nitrate medium, Table S7: Summary of leaching processes with other mineral acids/alkaline; Table S8: Summary of purification and extraction processes in other mineral acid/alkaline medium, Table S9: Summary of leaching processes with organic acids, Table S10: Summary of purification and extraction processes in organic acid solutions, and Table S11: Summary of direct recycling processes.

## References

[1] Tsiropoulos I., Lebedeva N. (2018). Li-ion Batteries for Mobility and Stationary Storage Applications. https://publications.europa.eu/en/publication-detail/-/publication/e65c072a-f389-11e8-9982-01aa75ed71a1/language-en/format-PDF/source-86566345.

[2] (Statistics_Canada) Households Energy Consumption in Canada. https://www150.statcan.gc.ca/t1/tbl1/en/tv.action?pid=2510006001.

[3] Winslow K.M., Laux S.J., Townsend T.G. (2018). A review on the growing concern and potential management strategies of waste lithium-ion batteries. Resour. Conserv. Recycl..

[4] Zheng X., Zhu Z., Lin X., Zhang Y., He Y., Cao H., Sun Z. (2018). A Mini-Review on Metal Recycling from Spent Lithium Ion Batteries. Engineering.

[5] Nigl T., Schwarz T., Arnberger A. Waste Li-Batteries – A Struggle to Safety in Waste Management Systems?. Proceedings of the 22nd ICBR - International Congress on Battery Recycling.

[6] Huang B., Pan Z., Su X., An L. (2018). Recycling of lithium-ion batteries: Recent advances and perspectives. J. Power Sources.

[7] Sonoc A., Jeswiet J., Soo V.K. (2015). Opportunities to Improve Recycling of Automotive Lithium Ion Batteries. Procedia CIRP.

[8] Gies E. (2015). Recycling: Lazarus batteries. Nature.

[9] Golmohammadzadeh R., Faraji F., Rashchi F. (2018). Recovery of lithium and cobalt from spent lithium ion batteries (LIBs) using organic acids as leaching reagents: A review. Resour. Conserv. Recycl..

[10] Gaines L., Richa K., Spangenberger J. (2018). Key issues for Li-ion battery recycling. MRS Energy Sustain..

[11] Yun L., Linh D., Shui L., Peng X., Garg A., LE M.L.P., Asghari S., Sandoval J. (2018). Metallurgical and mechanical methods for recycling of lithium-ion battery pack for electric vehicles. Resour. Conserv. Recycl..

[12] Bauer D., Diamond D., Li J., Sandalow D., Telleen P., Wanner B. (2010). Critical Materials Strategy.

[13] Emmanuel H., Marine S., Sokhna S.G. (2018). Electrification du parc Automobile Mondial et Criticite du Lithium a L’horizon 2050.

[14] Wang X., Gaustad G., Babbitt C.W., Richa K. (2014). Economies of scale for future lithium-ion battery recycling infrastructure. Resour. Conserv. Recycl..

[15] Brinkman B., Gotterbarn D., Miller K., Wolf M.J. (2016). 2016 Annual Report: Making a Positive Impact.

[16] (Eucobat) Position Paper - Collection Target for Waste Batteries. http://www.eucobat.eu/downloads.

[17] Zeng X., Li J., Singh N. (2014). Recycling of spent lithium-ion battery: A critical review. Crit. Rev. Environ. Sci. Technol..

[18] Lebedeva N., Persio F.D., Boon-brett L. (2016). Lithium Ion Battery Value Chain and Related Opportunities for EUROPE.

[19] Wang X., Wang X., Zhang R., Wang Y., Shu H. (2018). Hydrothermal preparation and performance of LiFePO_4_ by using Li_3_PO_4_ recovered from spent cathode scraps as Li source. Waste Manag..

[20] Yazami R., Touzain P.H. (1983). A reversible graphite-lithium electrochemical generators. J. Power Sources.

[21] Julien C., Mauger A., Vijh A., Zaghib K. (2016). Lithium Batteries.

[22] Doeff M.M., Brod R.J. (2013). Battery Cathodes. Encyclopedia of Sustainability Science and Technology.

[23] Zhou P., Meng H., Zhang Z., Chen C., Lu Y., Cao J., Cheng F., Chen J. (2017). Stable layered Ni-rich LiNi_0.9_Co_0.07_Al_0.03_O_2_ microspheres assembled with nanoparticles as high-performance cathode materials for lithium-ion batteries. J. Mater. Chem. A.

[24] Noh H.J., Youn S., Yoon C.S., Sun Y.K. (2013). Comparison of the structural and electrochemical properties of layered Li[Ni_x_Co_y_Mn_z_]O_2_ (x = 1/3, 0.5, 0.6, 0.7, 0.8 and 0.85) cathode material for lithium-ion batteries. J. Power Sources.

[25] Jiang Q., Wang X., Zhang H. (2016). One-Pot Hydrothermal Synthesis of LiMn_2_O_4_ Cathode Material with Excellent High-Rate and Cycling Properties. J. Electron. Mater..

[26] Tarascon J.-M., Armand M. (2001). Issues and challenges facing rechargeable lithium batteries. Nature.

[27] Silveira A.V.M., Santana M.P., Tanabe E.H., Bertuol D.A. (2017). Recovery of valuable materials from spent lithium ion batteries using electrostatic separation. Int. J. Miner. Process..

[28] Birkl C.R., Roberts M.R., Mcturk E., Bruce P.G., Howey D.A. (2017). Degradation diagnostics for lithium ion cells. J. Power Sources.

[29] Zhang W.-J. (2010). Structure and performance of LiFePO_4_ cathode materials: A review. J. Power Sources.

[30] Perea A., Paolella A., Dubé J., Champagne D., Mauger A., Zaghib K. (2018). State of charge influence on thermal reactions and abuse tests in commercial lithium-ion cells. J. Power Sources.

[31] Weise E. Cell Phones Thrown in the Trash are Exploding, Causing 5-alarm Fires in Garbage Trucks. https://www.usatoday.com/story/tech/talkingtech/2018/05/18/cell-phones-lithium-ion-batteries-exploding-causing-trash-fires/619897002/.

[32] Date W. Waste Industry Looks to Tackle Lithium-Ion Battery Fires. https://www.letsrecycle.com/news/latest-news/waste-industry-looks-to-tackle-lithium-ion-battery-fires/.

[33] Olapiriyakul S., Caudill R.J. (2008). A framework for risk management and end-of-life (EOL) analysis for nanotechnology products: A case study in lithium-ion batteries. IEEE Int. Symp. Electron. Environ..

[34] Yu A., Wei Y., Chen W., Peng N., Peng L. (2018). Life cycle environmental impacts and carbon emissions: A case study of electric and gasoline vehicles in China. Transp. Res. Part D Transp. Environ..

[35] Warburg N., Forell A., Guillon L., Teulon H., Canaguier B. (2013). Elaboration Selon les Principes des ACV des Bilans Energetiques, des Emissions de gaz a Effet de Serre et des Autres Impacts Environnementaux Induits par L’ensemble des Filieres de Vehicules Electriques et de Vehicules Thermiques, VP de Segment B.

[36] Majeau-Bettez G., Hawkins T.R., StrØmman A.H. (2011). Life cycle environmental assessment of lithium-ion and nickel metal hydride batteries for plug-in hybrid and battery electric vehicles. Environ. Sci. Technol..

[37] Oliveira L., Messagie M., Rangaraju S., Sanfelix J., Hernandez Rivas M., Van Mierlo J. (2015). Key issues of lithium-ion batteries - From resource depletion to environmental performance indicators. J. Clean. Prod..

[38] Dunn J.B., Gaines L., Kelly J.C., James C., Gallagher K.G. (2015). The significance of Li-ion batteries in electric vehicle life-cycle energy and emissions and recycling’s role in its reduction. Energy Environ. Sci..

[39] Xie J., Gao F., Gong X., Wang Z., Liu Y. (2018). Life Cycle Assessment of LFP Cathode Material Production for Power Lithium-Ion Batteries. Proceedings of the Advances in Energy and Environmental Materials.

[40] Xu J., Thomas H.R., Francis R.W., Lum K.R., Wang J., Liang B. (2008). A review of processes and technologies for the recycling of lithium-ion secondary batteries. J. Power Sources.

[41] Liang Y., Su J., Xi B., Yu Y., Ji D., Sun Y., Cui C., Zhu J. (2017). Life cycle assessment of lithium-ion batteries for greenhouse gas emissions. Resour. Conserv. Recycl..

[42] Zhang P., Yokoyama T., Itabashi O., Suzuki T.M., Inoue K. (1998). Hydrometallurgical process for recovery of metal values from spent lithium-ion secondary batteries. Hydrometallurgy.

[43] Zou H., Gratz E., Apelian D., Wang Y. (2013). A novel method to recycle mixed cathode materials for lithium ion batteries. Green Chem..

[44] Tedjar F. (2003). Recycling Used Electric Cells by Hydrometallurgical Treatment. World Patent.

[45] Tedjar F., Foudraz J.-C. (2005). Method for the Mixed Recycling of Lithium-based Aanode Batteries and Cells. World Patent.

[46] Tedjar F., Foudraz J.-C. (2007). Method for the Mixed Recycling of Lithium-Based Anode Batteries and Cells. US. Patent.

[47] Harper G., Sommerville R., Kendrick E., Driscoll L., Slater P., Stolkin R., Walton A., Christensen P., Heidrich O., Lambert S. (2019). Recycling lithium-ion batteries from electric vehicles. Nature.

[48] Gaines L., Sullivan J., Burnham A. (2011). Bel Life-Cycle Analysis for Lithium-Ion Battery Production and Recycling. Transp. Res. Board 90th Annu. Meet..

[49] Ziemann S., Müller D.B., Schebek L., Weil M. (2018). Modeling the potential impact of lithium recycling from EV batteries on lithium demand: A dynamic MFA approach. Resour. Conserv. Recycl..

[50] (SNAM) [Sale of Alloys]. http://www.snam.com/activites/ventes-alliage-suite.php.

[51] (Umicore) Our Recycling Process. https://csm.umicore.com/en/recycling/battery-recycling/our-recycling-process/.

[52] Zhang X., Li L., Fan E., Xue Q., Bian Y., Wu F., Chen R. (2018). Toward sustainable and systematic recycling of spent rechargeable batteries. Chem. Soc. Rev..

[53] Mayyas A., Steward D., Mann M. (2019). The case for recycling: Overview and challenges in the material supply chain for automotive li-ion batteries. Sustain. Mater. Technol..

[54] Pillot C. (2017). The Rechargeable Battery Market and Main Trends 2014–2025. Proceedings of the 22nd ICBR - International Congress on Battery Recycling.

[55] Houde S. (2019). Filière des batteries lithium-ion: Développer un secteur porteur d’avenir pour l’économie du Québec.

[56] Tedjar F., Foudraz J.-C. (2004). Procede de Recyclage en Melange de Piles et Batteries a Base d’anode en Lithium. French Patent.

[57] Recycling of Primary and Secondary Lithium Batteries (VALIBAT). https://cordis.europa.eu/project/rcn/51959/factsheet/en.

[58] Sloop S.E. (2016). Reintroduction of Lithium Into Recycled Battery Materials. U.S. Patent.

[59] Sloop S.E. (2009). Reintroduction of Lithium Into Recycled Battery Materials. U.S. Patent Application.

[60] Novis Smith W., Swoffer S. (2013). Recovery of Lithium Ion Batteries. U.S. Patent.

[61] Yinliang Z., Honghui T., Huan C., Qunying T., Du W. (2018). A Method of Recycling Valuable Metal from Waste and Old Lithium ion Battery. Chinese Patent.

[62] Morin D., Gagne-Bourque C., Nadeau E., Couture B. (2019). Lithium-ion Batteries Recycling Process. World Patent Application.

[63] Kim S., Yang D., Rhee K., Sohn J. (2014). Recycling process of spent battery modules in used hybrid electric vehicles using physical/chemical treatments. Res. Chem. Intermed..

[64] Hailey P., Kepler K. (2015). Direct Recycling Technology for Plug-in Electric Vehicle Lithium-Ion Battery Packs.

[65] Zhang T., He Y., Ge L., Fu R., Zhang X., Huang Y. (2013). Characteristics of wet and dry crushing methods in the recycling process of spent lithium-ion batteries. J. Power Sources.

[66] Li J., Wang G., Xu Z. (2015). Generation and detection of metal ions and volatile organic compounds (VOCs) emissions from the pretreatment processes for recycling spent lithium-ion batteries. Waste Manag..

[67] Zhang X., Xie Y., Lin X., Li H., Cao H. (2013). An overview on the processes and technologies for recycling cathodic active materials from spent lithium-ion batteries. J. Mater. Cycles Waste Manag..

[68] Dai Q. The economics of lithium-ion battery recycling. Proceedings of the NaatBatt Lithium-ion Battery Recycling Workshop.

[69] Kim H.S., Shin E.J. (2013). Re-synthesis and electrochemical characteristics of LiFePO_4_ cathode materials recycled from scrap electrodes. Bull. Korean Chem. Soc..

[70] Larouche F., Demopoulos G.P., Amouzegar K., Bouchard P., Zaghib K., Davis B. (2018). Recycling of Li-Ion and Li-Solid State Batteries: The Role of Hydrometallurgy. Extraction 2018.

[71] Wu C., Sun J., Zhu C., Ge Y., Zhao Y. Research on Overcharge and Overdischarge Effect on Lithium-Ion Batteries. Proceedings of the Vehicle Power and Propulsion (VPPC), IEEE Conference.

[72] Chen L., Tang X., Zhang Y., Li L., Zeng Z., Zhang Y. (2011). Process for the recovery of cobalt oxalate from spent lithium-ion batteries. Hydrometallurgy.

[73] Meshram P., Pandey B.D., Mankhand T.R. (2015). Recovery of valuable metals from cathodic active material of spent lithium ion batteries: Leaching and kinetic aspects. Waste Manag..

[74] Barik S.P., Prabaharan G., Kumar L. (2017). Leaching and separation of Co and Mn from electrode materials of spent lithium-ion batteries using hydrochloric acid: Laboratory and pilot scale study. J. Clean. Prod..

[75] Ebrahimzade H., Khayati G.R., Schaffie M. (2017). Preparation and kinetic modeling of β-Co(OH)_2_ nanoplates thermal decomposition obtained from spent Li-ion batteries. Adv. Powder Technol..

[76] Yang Y., Zheng X., Cao H., Zhao C., Lin X., Ning P., Zhang Y., Jin W., Sun Z. (2017). A Closed-Loop Process for Selective Metal Recovery from Spent Lithium Iron Phosphate Batteries through Mechanochemical Activation. ACS Sustain. Chem. Eng..

[77] Yang Y., Meng X., Cao H., Lin X., Liu C., Sun Y., Zhang Y., Sun Z. (2018). Selective recovery of lithium from spent lithium iron phosphate batteries: a sustainable process. Green Chem..

[78] Tanong K., Coudert L., Mercier G., Blais J.-F.F. (2016). Recovery of metals from a mixture of various spent batteries by a hydrometallurgical process. J. Environ. Manag..

[79] Xi G., Xu H., Yao L. (2015). Study on preparation of NiCo ferrite using spent lithium-ion and nickel-metal hydride batteries. Sep. Purif. Technol..

[80] Fricke J., Kiehne H.A. Collection and recycling of spent portable batteries-The actual situation in Europe. Proceedings of the TELESCON 2000-Third International Telecommunications Energy Special Conference.

[81] Coonen P., Allard G. Sorting, a profession in its own. Proceedings of the 22nd ICBR - International Congress on Battery Recycling.

[82] (Bebat) Sorting. https://www.bebat.be/en/sorting.

[83] Bernardes A.M., Espinosa D.C.R., Tenório J.A.S. (2004). Recycling of batteries: A review of current processes and technologies. J. Power Sources.

[84] Contestabile M., Panero S., Scrosati B. (1999). A laboratory-scale lithium battery recycling process. J. Power Sources.

[85] Gratz E., Sa Q., Apelian D., Wang Y. (2014). A closed loop process for recycling spent lithium ion batteries. J. Power Sources.

[86] Huang Y., Han G., Liu J., Chai W., Wang W., Yang S., Su S. (2016). A stepwise recovery of metals from hybrid cathodes of spent Li-ion batteries with leaching-flotation-precipitation process. J. Power Sources.

[87] Al-Thyabat S., Nakamura T., Shibata E., Iizuka A. (2013). Adaptation of minerals processing operations for lithium-ion (LiBs) and nickel metal hydride (NiMH) batteries recycling: Critical review. Miner. Eng..

[88] Chen Y., Liu N., Hu F., Ye L., Xi Y., Yang S. (2018). Thermal treatment and ammoniacal leaching for the recovery of valuable metals from spent lithium-ion batteries. Waste Manag..

[89] Wei J., Zhao S., Ji L., Zhou T., Miao Y., Scott K., Li D., Yang J., Wu X. (2018). Reuse of Ni-Co-Mn oxides from spent Li-ion batteries to prepare bifunctional air electrodes. Resour. Conserv. Recycl..

[90] Li L., Lu J., Ren Y., Zhang X.X., Chen R.J., Wu F., Amine K. (2012). Ascorbic-acid-assisted recovery of cobalt and lithium from spent Li-ion batteries. J. Power Sources.

[91] Yang Y., Huang G., Xu S., He Y., Liu X. (2016). Thermal treatment process for the recovery of valuable metals from spent lithium-ion batteries. Hydrometallurgy.

[92] Li W., Yang S., Liu N., Chen Y., Xi Y., Li S., Jie Y., Hu F., Gaustad G. (2019). Study on vacuum pyrolysis process of cathode sheets from spent lithium-ion batteries. Minerals, Metals and Materials Series.

[93] Zheng R., Zhao L., Wang W., Liu Y., Ma Q., Mu D., Li R., Dai C. (2016). Optimized Li and Fe recovery from spent lithium-ion batteries via a solution-precipitation method. RSC Adv..

[94] Jie Y., Yang S., Chen Y., Liu Z., Hu F., Gaustad G. (2019). Research on Thermogravimetric-Differential Scanning Calorimeter of Spent Lithium Iron Phosphate Batteries Cathode Plate. Proceedings of the REWAS 2019.

[95] Gaabour L.H. (2015). Thermal Spectroscopy and Kinetic Studies of PEO/PVDF Loaded by Carbon Nanotubes. J. Mater..

[96] Zucolotto V., Avlyanov J., Gregorio R., Mattoso L.H.C. (2004). Melt processing of composites of PVDF and carbon black modified with conducting polymers. J. Appl. Polym. Sci..

[97] Li X., Zhang J., Song D., Song J., Zhang L. (2017). Direct regeneration of recycled cathode material mixture from scrapped LiFePO_4_ batteries. J. Power Sources.

[98] Song X., Hu T., Liang C., Long H.L.L., Zhou L., Song W., You L., Wu Z.S., Liu J.W. (2017). Direct regeneration of cathode materials from spent lithium iron phosphate batteries using a solid phase sintering method. RSC Adv..

[99] Yang L., Xi G., Xi Y. (2015). Recovery of Co, Mn, Ni, and Li from spent lithium ion batteries for the preparation of LiNi_x_Co_y_Mn_z_O_2_ cathode materials. Ceram. Int..

[100] Nayaka G.P., Pai K.V., Manjanna J., Keny S.J. (2016). Use of mild organic acid reagents to recover the Co and Li from spent Li-ion batteries. Waste Manag..

[101] Zeng X., Li J. (2014). Innovative application of ionic liquid to separate Al and cathode materials from spent high-power lithium-ion batteries. J. Hazard. Mater..

[102] Zhou X., He W.Z., Li G.M., Zhang X.J., Huang J.W., Zhu S.G. Recycling of electrode materials from spent lithium-ion batteries. Proceedings of the 2010 4th International Conference on Bioinformatics and Biomedical Engineering: iCBBE 2010.

[103] Li J., Shi P., Wang Z., Chen Y., Chang C.-C. (2009). A combined recovery process of metals in spent lithium-ion batteries. Chemosp.

[104] Li L., Bian Y., Zhang X., Guan Y., Fan E., Wu F., Chen R. (2018). Process for recycling mixed-cathode materials from spent lithium-ion batteries and kinetics of leaching. Waste Manag..

[105] Nan J., Han D., Yang M., Cui M., Hou X. (2006). Recovery of metal values from a mixture of spent lithium-ion batteries and nickel-metal hydride batteries. Hydrometallurgy.

[106] Ren J., Li R., Liu Y., Cheng Y., Mu D., Zheng R., Liu J., Dai C. (2017). The impact of aluminum impurity on the regenerated lithium nickel cobalt manganese oxide cathode materials from spent LIBs. New J. Chem..

[107] Tsang F., Hailey P. (2016). Method for Removing Copper and Aluminum From an Electrode Mateiral, And Process for Recycling Electrode Material From Waste Lithium-Ion Batteries. U.S. Patent.

[108] Poe S.L., Paradise C.L., Muollo L.R., Pal R., Warmer J.C., Korzenski M.B. (2018). Method for the Recovery of Lithium Cobalt Oxide From Lithium Ion Batteries. U.S. Patent.

[109] Yu J., He Y., Ge Z., Li H., Xie W., Wang S. (2018). A promising physical method for recovery of LiCoO_2_ and graphite from spent lithium-ion batteries: Grinding flotation. Sep. Purif. Technol..

[110] He Y., Zhang T., Wang F., Zhang G., Zhang W., Wang J. (2017). Recovery of LiCoO_2_ and graphite from spent lithium-ion batteries by Fenton reagent-assisted flotation. J. Clean. Prod..

[111] Kepler K.D., Tsang F., Vermeulen R., Hailey P. (2016). Process for Recycling Electrode Materials from Lithium-ion Batteries. U.S. Patent.

[112] Fuerstenau M.C., Han K.N. (2003). Principles of Mineral Processing.

[113] Ordoñez J., Gago E.J., Girard A. (2016). Processes and technologies for the recycling and recovery of spent lithium-ion batteries. Renew. Sustain. Energy Rev..

[114] Meshram P., Pandey B.D., Mankhand T.R. (2014). Extraction of lithium from primary and secondary sources by pre-treatment, leaching and separation: A comprehensive review. Hydrometallurgy.

[115] Chagnes A., Pospiech B. (2013). A brief review on hydrometallurgical technologies for recycling spent lithium-ion batteries. J. Chem. Technol. Biotechnol..

[116] Wang W., Wu Y. (2017). An overview of recycling and treatment of spent LiFePO_4_ batteries in China. Resour. Conserv. Recycl..

[117] Espinosa D.C.R., Bernardes A.M., Tenório J.A.S. (2004). An overview on the current processes for the recycling of batteries. J. Power Sources.

[118] Friedrich B., Peters L. Status and Trends of industrialized Li-Ion battery recycling processes with qualitative comparison of economic and environmental impacts. Proceedings of the 22nd ICBR - International Congress on Battery Recycling.

[119] Gupta N., Prabaharan G. (2017). Process for Recovery of Pure Cobalt Oxide From Spent Lithium Ion Batteries With High Manganese Content. World Patent.

[120] Joulié M., Billy E., Laucournet R., Meyer D. (2017). Current collectors as reducing agent to dissolve active materials of positive electrodes from Li-ion battery wastes. Hydrometallurgy.

[121] Demopoulos G.P. (2009). Aqueous precipitation and crystallization for the production of particulate solids with desired properties. Hydrometallurgy.

[122] Wu Y., Pei F., Jia L., Tian X. (2014). Recovery of aluminum, iron and lithium from spent lithium iron phosphate batteries. Chinese J. Power Sources.

[123] Li H., Xing S., Liu Y., Li F., Guo H., Kuang G. (2017). Recovery of Lithium, Iron, and Phosphorus from Spent LiFePO_4_ Batteries Using Stoichiometric Sulfuric Acid Leaching System. ACS Sustain. Chem. Eng..

[124] Schurmans M., Thijs B. (2012). Process for the Recovery of Lithium and Iron from LFP Batteries. World Patent.

[125] Wohlgemuth D., Schneider M.A., Spielau R., Willems J., Steinbild M. (2014). Method for the Hydrometallurgical Recovery of Lithium from the Fraction of Used Galvanic Cells Containing Lithium, Iron and Phosphate. World Patent.

[126] Zheng Z., Chen M., Wang Q., Zhang Y., Ma X., Shen C., Xu D., Liu J., Liu Y., Gionet P. (2018). High Performance Cathode Recovery from Different Electric Vehicle Recycling Streams. ACS Sustain. Chem. Eng..

[127] Granata G., Moscardini E., Pagnanelli F., Trabucco F., Toro L. (2012). Product recovery from Li-ion battery wastes coming from an industrial pre-treatment plant: Lab scale tests and process simulations. J. Power Sources.

[128] Chen X., Guo C., Ma H., Li J., Zhou T., Cao L., Kang D. (2018). Organic reductants based leaching: A sustainable process for the recovery of valuable metals from spent lithium ion batteries. Waste Manag..

[129] Takacova Z., Havlik T., Kukurugya F., Orac D. (2016). Cobalt and lithium recovery from active mass of spent Li-ion batteries: Theoretical and experimental approach. Hydrometallurgy.

[130] Prabaharan G., Barik S.P., Kumar N., Kumar L. (2017). Electrochemical process for electrode material of spent lithium ion batteries. Waste Manag..

[131] Dutta D., Kumari A., Panda R., Jha S., Gupta D., Goel S. (2018). Separation and Puri fi cation Technology Close loop separation process for the recovery of Co, Cu, Mn, Fe and Li from spent lithium-ion batteries. Sep. Purif. Technol..

[132] Zhu S.G., He W.Z., Li G.M., Zhou X., Zhang X.J., Huang J.W. (2012). Recovery of Co and Li from spent lithium-ion batteries by combination method of acid leaching and chemical precipitation. Trans. Nonferrous Met. Soc. China (Engl. Ed.).

[133] Jo C.H., Myung S.T. (2019). Efficient recycling of valuable resources from discarded lithium-ion batteries. J. Power Sources.

[134] Higuchi A., Ankei N., Nishihama S., Yoshizuka K. (2016). Selective Recovery of Lithium from Cathode Materials of Spent Lithium Ion Battery. JOM.

[135] Aaltonen M., Peng C., Wilson B., Lundström M. (2017). Leaching of Metals from Spent Lithium-Ion Batteries. Recycling.

[136] Vieceli N., Nogueira C.A., Guimarães C., Pereira M.F.C., Durão F.O., Margarido F. (2018). Hydrometallurgical recycling of lithium-ion batteries by reductive leaching with sodium metabisulphite. Waste Manag..

[137] Chen H., Zhu X., Chang Y., Cai J., Zhao R. (2018). 3D flower-like CoS hierarchitectures recycled from spent LiCoO_2_ batteries and its application in electrochemical capacitor. Mater. Lett..

[138] Shin S.M., Kim N.H., Sohn J.S., Yang D.H., Kim Y.H. (2005). Development of a metal recovery process from Li-ion battery wastes. Hydrometallurgy.

[139] Meshram P., Abhilash, Pandey B.D., Mankhand T.R., Deveci H. (2016). Acid baking of spent lithium ion batteries for selective recovery of major metals: A two-step process. J. Ind. Eng. Chem..

[140] Sun L., Qiu K. (2011). Vacuum pyrolysis and hydrometallurgical process for the recovery of valuable metals from spent lithium-ion batteries. J. Hazard. Mater..

[141] Sohn J.S., Shin S.M., Yang D.H., Kim S.K., Lee C.K. (2006). Comparison of two acidic leaching processes for selecting the effective recycle process of spent lithium ion battery. Geosystem Eng..

[142] Kang J., Sohn J., Chang H., Senanayake G., Shin S.M. (2010). Preparation of cobalt oxide from concentrated cathode material of spent lithium ion batteries by hydrometallurgical method. Adv. Powder Technol..

[143] Zhao J., Qu X., Qu J., Zhang B., Ning Z., Xie H., Zhou X., Song Q., Xing P., Yin H. (2019). Extraction of Co and Li_2_CO_3_ from Cathode Materials of Spent Lithium-ion Batteries through a Combined Acid-leaching and Electro-deoxidation Approach. J. Hazard. Mater..

[144] Atia T.A., Elia G., Hahn R., Altimari P., Pagnanelli F. (2019). Closed-loop hydrometallurgical treatment of end-of-life lithium ion batteries: Towards zero-waste process and metal recycling in advanced batteries. J. Energy Chem..

[145] Wohlgemuth D., Schneider M.A., Spielau R., Willems J., Steinbild M. (2017). Method for the Hydrometallurgical Recovery of Lithium, Nickel and Cobalt From the Lithium Transition Metal Oxide-Containing Fraction of Used Galvanic Cells. World Patent.

[146] Chow N., Jung J.C.-Y., Nacu A.M., Warkentin D.D. (2018). Processing of Cobaltous Sulphate/Dithionate Liquors Derived From Cobalt Resource. World Patent.

[147] Hu J., Zhang J., Li H., Chen Y., Wang C. (2017). A promising approach for the recovery of high value-added metals from spent lithium-ion batteries. J. Power Sources.

[148] Dorella G., Mansur M.B. (2007). A study of the separation of cobalt from spent Li-ion battery residues. J. Power Sources.

[149] Weng Y., Xu S., Huang G., Jiang C. (2013). Synthesis and performance of Li[(Ni_1/3_Co_1/3_Mn_1/3_)_1-x_Mg_x_]O_2_ prepared from spent lithium ion batteries. J. Hazard. Mater..

[150] Yang Y., Xu S., He Y. (2017). Lithium recycling and cathode material regeneration from acid leach liquor of spent lithium-ion battery via facile co-extraction and co-precipitation processes. Waste Manag..

[151] Nan J., Han D., Zuo X. (2005). Recovery of metal values from spent lithium-ion batteries with chemical deposition and solvent extraction. J. Power Sources.

[152] Sohn J.S., Yang D.H., Shin S.M., Kang J.G. (2006). Recovery of cobalt in sulfuric acid leaching solution using oxalic acid. Geosystem Eng..

[153] Lupi C., Pasquali M., Dell’Era A. (2005). Nickel and cobalt recycling from lithium-ion batteries by electrochemical processes. Waste Manag..

[154] Mantuano D.P., Dorella G., Elias R.C.A., Mansur M.B. (2006). Analysis of a hydrometallurgical route to recover base metals from spent rechargeable batteries by liquid-liquid extraction with Cyanex 272. J. Power Sources.

[155] Nguyen V.T., Lee J.C., Jeong J., Kim B.S., Pandey B.D. (2014). Selective recovery of cobalt, nickel and lithium from sulfate leachate of cathode scrap of Li-ion batteries using liquid-liquid extraction. Met. Mater. Int..

[156] Nguyen T., Lee M. (2018). A Review on the Separation of Lithium Ion from Leach Liquors of Primary and Secondary Resources by Solvent Extraction with Commercial Extractants. Processes.

[157] Swain B., Jeong J., Yoo K., Lee J. (2010). chun Synergistic separation of Co(II)/Li(I) for the recycling of LIB industry wastes by supported liquid membrane using Cyanex 272 and DR-8R. Hydrometallurgy.

[158] Chung K.Y., Lee H.Y., Cho B.-W. (2014). Recovery and Synthesis Method for Metaloxidic Cathodic Active Material for Lithium ion Secondary Battery. U.S. Patent.

[159] He L.P., Sun S.Y., Yu J.G. (2018). Performance of LiNi_1/3_Co_1/3_Mn_1/3_O_2_ prepared from spent lithium-ion batteries by a carbonate co-precipitation method. Ceram. Int..

[160] Tedjar F., Foudraz J.-C. (2010). Method for the Mixed Recycling of Lithium-Based Anode Batteries and Cells. U.S. Patent.

[161] Shin S.M., Ju S.-H. (2017). Method for Simultaneously Recovering Cobalt and Manganese from Lithium Based Battery. U.S. Patent.

[162] Freitas M.B.J.G., Garcia E.M. (2007). Electrochemical recycling of cobalt from cathodes of spent lithium-ion batteries. J. Power Sources.

[163] Shuva M.A.H., Kurny A.S.W. (2013). Dissolution Kinetics of Cathode of Spent Lithium Ion Battery in Hydrochloric Acid Solutions. J. Inst. Eng. Ser. D Metall. Mater. Min. Eng..

[164] Laucournet R., Barthelemy S., Diaferia N. (2016). Method for recycling lithium batteries and/or electrodes of such batteries. U.S. Patent.

[165] Taniyama K., Haraguchi K., Shimoyama K. (2012). Lithium-Ion Battery Capacity Recovery Method. World Patent.

[166] Shin E.J., Kim S., Noh J.-K., Byun D., Chung K.Y., Kim H.-S., Cho B.-W. (2015). A green recycling process designed for LiFePO_4_ cathode materials for Li-ion batteries. J. Mater. Chem. A.

[167] Kim H.S., Cho B.-W., Lee H.Y., Shin E.J., Kim S., Chung K.Y. (2017). Recycling Method of Olivine-Based Cathode Material for Lithium Secondary Battery, Cathode Material Fabricated Therefrom, and Cathode and Lithium Secondary Battery Including the Same. U.S. Patent.

[168] Kim H.-S., Cho B.-W., Lee H.Y., Kim S.J., Shin E.J., Chung K.Y. (2015). Method of Fabricating LiFePO_4_ Cathode Electroactive Material by Recycling, and LiFePO_4_ Cathode electroactive Material, LiFePO_4_ Cathode, and Lithium Secondary Battery Fabricated Thereby. U.S. Patent.

[169] Contestabile M., Panero S., Scrosati B. (2001). Laboratory-scale lithium-ion battery recycling process. J. Power Sources.

[170] Boxall N.J., Adamek N., Cheng K.Y., Haque N., Bruckard W., Kaksonen A.H. (2018). Multistage leaching of metals from spent lithium ion battery waste using electrochemically generated acidic lixiviant. Waste Manag..

[171] Wang R.-C., Lin Y.-C., Wu S.-H. (2009). A novel recovery process of metal values from the cathode active materials of the lithium-ion secondary batteries. Hydrometallurgy.

[172] Porvali A., Aaltonen M., Ojanen S., Velazquez-Martinez O., Eronen E., Liu F., Wilson B.P., Serna-Guerrero R., Lundström M. (2019). Mechanical and hydrometallurgical processes in HCl media for the recycling of valuable metals from Li-ion battery waste. Resour. Conserv. Recycl..

[173] Castillo S., Ansart F., Laberty-Robert C., Portal J. (2002). Advances in the recovering of spent lithium battery compounds. J. Power Sources.

[174] Guan J., Li Y., Guo Y., Su R., Gao G., Song H., Yuan H., Liang B., Guo Z. (2017). Mechanochemical Process Enhanced Cobalt and Lithium Recycling from Wasted Lithium-Ion Batteries. ACS Sustain. Chem. Eng..

[175] Lee C.K., Rhee K.I. (2003). Reductive leaching of cathodic active materials from lithium ion battery wastes. Hydrometallurgy.

[176] Moura M.N., Barrada R.V., Almeida J.R., Moreira T.F.M., Schettino M.A., Freitas J.C.C., Ferreira S.A.D., Lelis M.F.F., Freitas M.B.J.G. (2017). Synthesis, characterization and photocatalytic properties of nanostructured CoFe_2_O_4_ recycled from spent Li-ion batteries. Chemosphere.

[177] Yang Y., Guo J.-Z., Gu Z.-Y., Sun Z.-H., Hou B.-H., Yang A.-B., Ning Q.-L., Li W.-H., Wu X.-L. (2019). Effective Recycling of the Whole Cathode in Spent Lithium Ion Batteries: From the Widely Used Oxides to High-Energy/Stable Phosphates. ACS Sustain. Chem. Eng..

[178] Li L., Zhai L., Zhang X., Lu J., Chen R., Wu F., Amine K. (2014). Recovery of valuable metals from spent lithium-ion batteries by ultrasonic-assisted leaching process. J. Power Sources.

[179] Myoung J., Jung Y., Lee J., Tak Y. (2002). Cobalt oxide preparation from waste LiCoO_2_ by electrochemical-hydrothermal method. J. Power Sources.

[180] Li L., Chen R., Sun F., Wu F., Liu J. (2011). Preparation of LiCoO_2_ films from spent lithium-ion batteries by a combined recycling process. Hydrometallurgy.

[181] Meng Q., Zhang Y., Dong P. (2017). Use of glucose as reductant to recover Co from spent lithium ions batteries. Waste Manag..

[182] Pinna E.G., Ruiz M.C., Ojeda M.W., Rodriguez M.H. (2017). Cathodes of spent Li-ion batteries: Dissolution with phosphoric acid and recovery of lithium and cobalt from leach liquors. Hydrometallurgy.

[183] Bian D., Sun Y., Li S., Tian Y., Yang Z., Fan X., Zhang W. (2016). A novel process to recycle spent LiFePO_4_ for synthesizing LiFePO_4_/C hierarchical microflowers. Electrochim. Acta.

[184] Suarez D.S., Pinna E.G., Rosales G.D., Rodriguez M.H. (2017). Synthesis of Lithium Fluoride from Spent Lithium Ion Batteries. Minerals.

[185] Amouzegar K., Bouchard P., Turcotte N., Zaghib K. (2017). Method for Recycling Electrode Materials of a Lithium Battery. World Patent.

[186] Chen X., Ma H., Luo C., Zhou T. (2017). Recovery of valuable metals from waste cathode materials of spent lithium-ion batteries using mild phosphoric acid. J. Hazard. Mater..

[187] Wu C., Li B., Yuan C., Ni S., Li L. (2019). Recycling valuable metals from spent lithium-ion batteries by ammonium sulfite-reduction ammonia leaching. Waste Manag..

[188] Zhang J., Hu J., Liu Y., Jing Q., Yang C., Chen Y., Wang C. (2019). Sustainable and Facile Method for the Selective Recovery of Lithium from Cathode Scrap of Spent LiFePO_4_ Batteries. ACS Sustain. Chem. Eng..

[189] Chen X., Luo C., Zhang J., Kong J., Zhou T. (2015). Sustainable Recovery of Metals from Spent Lithium-Ion Batteries: A Green Process. ACS Sustain. Chem. Eng..

[190] Golmohammadzadeh R., Rashchi F., Vahidi E. (2017). Recovery of lithium and cobalt from spent lithium-ion batteries using organic acids: Process optimization and kinetic aspects. Waste Manag..

[191] Zheng Y., Song W., Mo W., Zhou L., Liu J.-W. (2018). Lithium fluoride recovery from cathode material of spent lithium-ion battery. RSC Adv..

[192] Rumble J.R., Lide D.R., Bruno T.J.T.A.-T.T. (2018). CRC Handbook of Chemistry and Physics.

[193] Gao W., Zhang X., Zheng X., Lin X., Cao H., Zhang Y., Sun Z. (2017). Lithium Carbonate Recovery from Cathode Scrap of Spent Lithium-Ion Battery: A Closed-Loop Process. Environ. Sci. Technol..

[194] Poling B.E., Thomson G.H., Friend D.G., Rowley R.L., Engineering C., Wilding W.V., Perry R.H., Green D.W. (2008). Physical and Chemical Data *. Perry’s Chemical Engineers’ Handbook.

[195] Gao W., Song J., Cao H., Lin X., Zhang X., Zheng X., Zhang Y., Sun Z. (2018). Selective recovery of valuable metals from spent lithium-ion batteries – Process development and kinetics evaluation. J. Clean. Prod..

[196] Liu K., Zhang F.S. (2016). Innovative leaching of cobalt and lithium from spent lithium-ion batteries and simultaneous dechlorination of polyvinyl chloride in subcritical water. J. Hazard. Mater..

[197] Nayaka G.P., Pai K.V., Santhosh G., Manjanna J. (2016). Recovery of cobalt as cobalt oxalate from spent lithium ion batteries by using glycine as leaching agent. J. Environ. Chem. Eng..

[198] Li L., Ge J., Wu F., Chen R., Chen S., Wu B. (2010). Recovery of cobalt and lithium from spent lithium ion batteries using organic citric acid as leachant. J. Hazard. Mater..

[199] Li L., Zhang X., Chen R., Zhao T., Lu J., Wu F., Amine K. (2014). Synthesis and electrochemical performance of cathode material Li_1.2_Co_0.13_Ni_0.13_Mn_0.54_O_2_ from spent lithium-ion batteries. J. Power Sources.

[200] Pant D., Dolker T. (2017). Green and facile method for the recovery of spent Lithium Nickel Manganese Cobalt Oxide (NMC) based Lithium ion batteries. Waste Manag..

[201] Yao L., Feng Y., Xi G. (2015). A new method for the synthesis of LiNi_1/3_ Co_1/3_ Mn_1/3_ O_2_ from waste lithium ion batteries. RSC Adv..

[202] Wang M.M., Zhang C.C., Zhang F.S. (2016). An environmental benign process for cobalt and lithium recovery from spent lithium-ion batteries by mechanochemical approach. Waste Manag..

[203] Nayaka G.P., Manjanna J., Pai K.V., Vadavi R., Keny S.J., Tripathi V.S. (2015). Recovery of valuable metal ions from the spent lithium-ion battery using aqueous mixture of mild organic acids as alternative to mineral acids. Hydrometallurgy.

[204] Li L., Bian Y., Zhang X., Yao Y., Xue Q., Fan E., Wu F., Chen R. (2019). A green and effective room-temperature recycling process of LiFePO_4_ cathode materials for lithium-ion batteries. Waste Manag..

[205] Wang B., Lin X.-Y., Tang Y., Wang Q., Leung M.K.H., Lu X.-Y. (2019). Recycling LiCoO_2_ with methanesulfonic acid for regeneration of lithium-ion battery electrode materials. J. Power Sources.

[206] Zeng X., Li J., Shen B. (2015). Novel approach to recover cobalt and lithium from spent lithium-ion battery using oxalic acid. J. Hazard. Mater..

[207] Li L., Lu J., Zhai L., Zhang X., Curtiss L., Jin Y., Wu F., Chen R., Amine K. (2018). A facile recovery process for cathodes from spent lithium iron phosphate batteries by using oxalic acid. CSEE J. Power Energy Syst..

[208] He L.P., Sun S.Y., Mu Y.Y., Song X.F., Yu J.G. (2017). Recovery of Lithium, Nickel, Cobalt, and Manganese from Spent Lithium-Ion Batteries Using L-Tartaric Acid as a Leachant. ACS Sustain. Chem. Eng..

[209] Okayam S., Uchida S. (2018). Degrade Performance Recovery Method for Lithium Ion Secondary Battery. U.S. Patent.

[210] Petzl M., Danzer M.A. (2014). Nondestructive detection, characterization, and quantification of lithium plating in commercial lithium-ion batteries. J. Power Sources.

[211] He L., Zhao Z., Liu X., Chen A., Si X. (2012). Thermodynamics analysis of LiFePO_4_ precipitation from Li–Fe(II)–P–H_2_O system at 298 K. Trans. Nonferrous Met. Soc. China.

[212] Hamada K., Sugibuchi Y., Kashima H. (2017). Treatment Process for a Positive Electrode Active Material for Lithium-Ion Secondary Battery. U.S. Patent.

[213] Chen J., Li Q., Song J., Song D., Zhang L., Shi X. (2016). Environmentally friendly recycling and effective repairing of cathode powders from spent LiFePO_4_ batteries. Green Chem..

[214] Novis Smith W., Swoffer S. (2014). Process for Recovering and Regenerating Lithium cathode Material From Lithium-Ion Batteries. U.S. Patent.

[215] Sloop S.E. (2017). Relithiation in Oxidizing Conditions. U.S. Patent.

[216] Shi Y., Chen G., Chen Z. (2018). Effective regeneration of LiCoO_2_ from spent lithium-ion batteries: a direct approach towards high-performance active particles. Green Chem..

[217] Ganter M.J., Landi B.J., Babbitt C.W., Anctil A., Gaustad G. (2014). Cathode refunctionalization as a lithium ion battery recycling alternative. J. Power Sources.

[218] Sloop S.E., Trevey J., Gaines L., Lerner M.M., Xu W. (2018). Advances in Direct Recycling of Lithium-Ion Electrode Materials. ECS Trans..

[219] Ra D.-I., Han K.-S. (2006). Used lithium ion rechargeable battery recycling using Etoile-Rebatt technology. J. Power Sources.

[220] Zhu S., Qin Y., Zhang J. (2016). Renovation of lithium cobalt oxide from spent lithium ion batteries by an aqueous pulsed discharge plasma. Int. J. Electrochem. Sci..

[221] Kepler D.K., Tsang F., Vermeulen R., Hailey P. (2016). Process for Preparing and Recycling Cathode Active Materials for Lithium-ion Batteries. U.S. Patent.

[222] Yang Z., Zhang J., Wu Q., Zhi L., Zhang W. (2013). Electrochemical Regeneration of LiFePO_4_/C CathodeMaterials fromSpent Lithium Ion Batteries. J. Chinese Ceram. Soc..

[223] Wang Q., Liu Y., Yanhong C., Jinping W., Liangliang L., Yanhua L. (2011). Method for Treating Lithium Iron Phosphate Cathode Material of Waste and Old Power Lithium Battery of Automobile. Chinese Patent.

[224] Xie Y., Yu H., Ou Y., Li C. (2014). Recovery and preparation of LiFePO_4_ from used traction battery. Chinese J. Power Sources.

[225] Wang L., Li J., Zhou H., Huang Z., Tao S., Zhai B., Liu L., Hu L. (2018). Regeneration cathode material mixture from spent lithium iron phosphate batteries. J. Mater. Sci. Mater. Electron..

[226] Shi Y., Zhang M., Meng Y.S., Chen Z. (2019). Ambient-Pressure Relithiation of Degraded Li_x_Ni_0.5_Co_0.2_Mn_0.3_O_2_ (0 < x <1) via Eutectic Solutions for Direct Regeneration of Lithium-Ion Battery Cathodes. Adv. Energy Mater..

[227] Zhong X., Liu W., Han J., Jiao F., Qin W., Liu T., Zhao C. (2019). Pyrolysis and physical separation for the recovery of spent LiFePO_4_ batteries. Waste Manag..

[228] Bi H., Zhu H., Zu L., He S., Gao Y., Gao S. (2019). Pneumatic separation and recycling of anode and cathode materials from spent lithium iron phosphate batteries. Waste Manag. Res..

[229] Baumann M., Peters J.F., Weil M., Grunwald A. (2017). CO_2_ Footprint and Life-Cycle Costs of Electrochemical Energy Storage for Stationary Grid Applications. Energy Technol..

[230] Vandepaer L., Cloutier J., Bauer C., Amor B. (2018). Integrating Batteries in the Future Swiss Electricity Supply System: A Consequential Environmental Assessment. J. Ind. Ecol..

[231] Majeau-Bettez G., Hawkins T.R., Strømman A.H. (2011). Life Cycle Environmental Assessment of Li-ion and Nickel Metal Hydride Batteries for Plug-in Hybrid and Battery Electric Vehicles. Supporting Information. Zhurnal Eksp. i Teor. Fiz..

[232] Tedjar F. Sustainability of battery segment with recycling of strategic metals. Proceedings of the Circular Economy Batteries Conference.

[233] Tedjar F. Circular economy around EV Batteries. Proceedings of the E-Mobility & Circular Economy Conference.

[234] Kincaide J. Where Are Batteries Ending Up Now?. Proceedings of the NaatBatt Lithium-ion Battery Recycling Workshop.

