# Supplementary material for: Progress and Status of Hydrometallurgical and Direct Recycling of Li-Ion Batteries and Beyond
Source: Materials (Basel). 2020 Feb 10;13(3):801. doi: 10.3390/ma13030801 (PMC7040742; doi:10.3390/ma13030801)
Supplement: Supplementary file 1 [file materials-13-00801-s001.pdf]

materials

Supplementary Materials

Progress and Status of Hydrometallurgical and Direct Recycling of Li-Ion Batteries and Beyond

François Larouche [1,2], Farouk Tedjar [3], Kamyab Amouzegar [1], Georges Houlachi [4], Patrick Bouchard [1], George P. Demopoulos [2,*] and Karim Zaghib [1,*]

1 Center of Excellence in Transportation Electrification and Energy Storage (CETEES), Hydro-Québec, 1806, Lionel-Boulet Blvd., Varennes, QC J3X 1S1, Canada; larouche.francois3@hydro.qc.ca (F.L.); amouzegar.kamyab@hydro.qc.ca (K.A.); bouchard.patrick@ireq.ca (P.B.)
2 Mining and Materials Engineering, McGill University, 3610 University Street, Montréal, QC H3A 0C5, Canada
3 Energy Research Institute, NTU, 1 Cleantech loop, Singapore 634672; farouk.tedjar@ntu.edu.sg
4 Centre de Recherche d'Hydro-Québec (CRHQ), 600, avenue de la Montagne, Shawinigan, QC G9N 7N5, Canada; houlachi.georges@ireq.ca
* Correspondence: george.demopoulos@mcgill.ca (G.P.D.); zaghib.karim@hydro.qc.ca (K.Z.)

Received: 21 December 2019; Accepted: 4 February 2020; Published: date

This supporting document provides master summary tables (Tables S1–S11 in Appendix) presenting information about processes discussed in the paper. The data provided for each publication reflect optimal process conditions and the process adopted by the authors. The data are grouped by the type of leaching agent, with a specific table dedicated for direct recycling. For each leaching agent, one table presents information about pretreatment and the leaching step and another table refers to subsequent purification steps. All studies may not be found in both tables because some may have focused mainly on optimizing the leaching step while others may have focused on optimizing the post-leaching process. In such a case, only the most relevant information provided with enough detail on test work methodologies is summarized in the tables.

Below are some table specific guidelines to read them.

Leaching conditions summary table

Column 1: Reference

The numbers listed in this column correspond to reference numbers in the Bibliography section of the paper.

Column 2: Pretreatment

This column describes the pretreatment steps applied to spent batteries to prepare the cathodic active material prior to leaching. The process for treating the anodic active material when it is distinct from the recovery method of cathodic material is not considered in the table. The steps are chronological, starting from spent cells down to the leach feed material. Any separation step involves generation of waste or by-product, for which the post-treatment is not described therein. For instance, for "manual separation," it is understood that the components of the battery are sorted out while leaving only the cathode for further steps. In several cases, the cathode has been submitted to alkaline leaching during which the aluminum foil is dissolved in caustic solution.

Column 3: Treated battery types

This column lists the chemistry of the LIB being treated by the process.

Column 4: Leach feed

This column lists the nature of the leach feed. It is the valuable product resulting from pretreatment. The term 'active material' refers only to the lithium-ion intercalation compounds, such as $LiFePO_4$. The term 'black mass' refers to the cathodic active layer coated on the current collector foil. It includes the lithium-ion intercalation compounds, conductive compound, and binder (most frequently PVDF). When both anode and cathode are leached together, it is specified in the cell. Otherwise, the leaching process described therein focuses only on cathodic valuable material. The terms 'cathode' and 'anode' denote the whole electrode, including the current collector. During pretreatment, the electrode might have been calcined at a high temperature, resulting in oxidized active material liberated from carbon and PVDF binder. Finally, some authors preformed leaching tests using only fresh active material, which is specified in this column.

Column 5: Reducing or oxidizing agent

This column lists the reducing (R) or oxidizing (O) agent used in the leaching step. When no such agent is used, the term "None" is written.

Column 6: Special leaching conditions

This lists any special leaching condition(s) other than temperature, chemical concentration, leaching time, or solid-to-liquid ratio. The condition may be related to auxiliary equipment that accelerates the leaching reaction such as ultrasonic or electrochemically supported leaching. It can also be a detail regarding the leaching or reagent used.

Column 7: Acid concentration

This column lists the optimal concentration of acid. For consistency and ease of comparison, calculations have been reworked in some cases to express the units in mole per liter (M) for all studies.

Column 8: Reducer/oxidizer concentration

This column lists the optimal concentration of reducing or oxidizing agent. For consistency and ease of comparison, calculations have been reworked in some cases to express the units in mole per liter (M) for all studies.

Column 9: Temperature

This column lists the optimal reaction temperature in degree Celsius (°C).

Column 10: Leaching time

This column lists the reaction time in minutes (min). The reaction time is not necessarily the optimal one, but it corresponds to the time stated in the publication to reach the maximum recovery rate under the conditions specified herein.

Colum 11: Solid concentration

This column lists the optimal solid concentration. The solid corresponds to the leach feed listed in Column 4. When the authors used different units, the solid concentration was back calculated from data provided in the publication. For consistency and ease of comparison, calculations have been reworked in some cases to express the units in gram per liter (g/L) of solute for all studies.

Column 12: Leaching rate (%)

This column lists the leaching yield for major elements. The values have been either taken directly from the studies or calculated based on the data therein.

Purification processes summary table

Column 1: Reference

The numbers listed in this column correspond to reference numbers in the Bibliography section of the paper.

Column 2: Purification process (Reagents)

This column lists the sequence of purification steps. The reagent used is enclosed within brackets.

Column 3: Final product(s)

This column lists each product and tails, unless gaseous emission is coming from combustion.

Column 4: Recovery rate (%)

This column lists the recovery of major components wherever available. It refers to the elemental recovery through the entire purification process toward valuable products. The values have been either taken directly from the studies or calculated based on the data therein.

Column 5: Comments

This column lists additional details on the process described. More specifically, when the LIB active material is one of the end products of the process, a summary of electrochemical capacity tests is given. This information has been either culled out from Results and Discussion sections of the publication or derived from data therein.

Summary tables for direct recycling processes

Column 1: Reference

The numbers listed in this column correspond to reference numbers in the Bibliography section of the paper.

Column 2: Treated battery type(s)

This column lists the chemistry/chemistries of LIB(s) treated by the process.

Column 3: Process steps

This column lists the process steps in a chronological order. For each step, the reagent used is given in brackets.

Column 4: Temperature (°C)/other parameter

This column lists the main process parameter for each process step given in Column 3. The parameter is vertically aligned with the corresponding process step specified in Column 3.

Column 5: Final products

In this column, a list of products and their states, including waste, is given. The gaseous waste from combustion is excluded from the list.

Column 6: Re-evaluated capacity (mAh/g)

Direct recycling aims to transform waste lithium-ion waste active material into new one. In this regard, most studies provide a comparative evaluation of electrochemical properties of the final product. The results of this analysis are shown in Column 6. To allow comparison between each process, results from standard electrochemical test conditions were given priority over others. These conditions are the following: Initial specific capacity at C/10, initial specific capacity at 1 C, and

capacity retention at 1 C. However, given the heterogeneity of electrochemical characterization methods among all studies considered herein, this principle could not be respected all the time.

Appendix

Table S1. Summary of leaching processes with sulfuric acid; here, LCO, NCO, NCA, NMC, LMO, and LFP-C are lithium-cobalt oxide, lithium-nickel-cobalt oxide, lithium-nickel-aluminum oxide, lithium-nickel-manganese-cobalt, lithium-manganese-oxide, and lithium-iron-phosphate–carbon, respectively; N.A. stands for “Not Applicable”; N.S. stands for “Not Specified”.

Reference(s)	Pretreatment	Treated Battery Type(s)	Leach feed	Reducing [R]/ Oxidizing [O] Agent	Special Leaching Condition(s)	Acid Concentration (M)	Reducer/Oxidizer Concentration (M)	*T* (°C)	Leaching Time (min)	Solid Concentration (g/L)	Recovery Rate (%)	Pros	Cons
[160]	Crushing/Sieving Magnetic Separation Densimetric Separation Alkaline leaching	LCO-LMO-NMC	Anodic and Cathodic Black Mass	Iron [R]		1	Ratio Steel/Black Mass = 0.15	80	N.S.	N.S.	N.S.	Proven technology	
[145]	N.S.	NMC	Black Mass	H_2O_2 [R]		2	30% Molar Excess	55	150	30–230	Li = 100% Co = 100% Ni = 100% Al = 100%	High efficiencies	Long reaction time
[132]	Manual Separation	LCO	Black Mass	H_2O_2 [R]	50 W Ultrasonic	2	0.85	60	120	33	Li = 88% Co = 96%	Low H_2O_2 concentration	Low solid to liquid ratio
[136]	Cryogenic Shredding Magnetic Separation Sieving Density Separation Grinding	NMC	Anode and Cathode	$Na_2S_2O_5$ [R]		1.25	0.1	60	90	1.5	Li = 95% Co = 100% Ni = 100% Mn = 85%	Low cost reducing agent	Low solid to liquid ratio

[128]	Dismantling Manual Separation Peeling Black Mass Calcining	LCO	Calcined Active Material	Sucrose or Glucose [R]		3	0.86	95	120	25	Li = 96% Co = 98%	Low cost reducing agent	Low solid to liquid ratio
[127]	Industrial Scale Mechanical Method (Shredding, Sieving & Physical Separation)	LCO	Cathode + Anode	Glucose [R]		2	50% Excess	90	180	100	Li = 97% Co = 98%	Treatment of cathode and anode	Only applicable to LCO<br>Long reaction time
[137]	Binder Dissolution (NMP) Calcining (400 °C for 2 h)	LCO	Active Material	H_2O_2 [R]		1.2	0.59	80	90	50	N.A.	-	-
[72]	Dismantling Manual Separation Shredding Drying (200 °C) Alkaline Leaching (5% NaOH) Thermal (700 °C for 4 h)	Blend of LIBs	Calcined Active Material	H_2O_2 [R]		4	4.26	85	120	100	Li = 96% Co = 95%	High solid to liquid ratio	Long reaction time
[130]	Water Shredding Density Separation Sieving (30 mesh) Drying Calcining (500 °C for 1 h)	NMC-LCO-LMO	Active Material	Electrolysis	400 A/m²	2	N.A.	N.S.	180	75	Co = 99% Mn = 99% Cu = 99%	Process at pilot stage	Long reaction time<br>Low current density leading to expensive electrolysis infrastructure

[138]	Crushing Sieving Magnetic Separation Crushing/ Sieving	LCO	Black Mass	H_2O_2 [R]	2	6.40	75	30	50	Co = 100% Li = 100%	High efficiencies	Low solid to liquid ratio
[158]	N.S.	NMC NCA	Cathode	$SO_{2(g)}$ [R]	0.5–2	Saturated	25	60	10–50	Mass Recovery = 93%	Low cost reducing agent	Low recovery rate
[161]	Thermal (500 °C) Shredding Sieving (12 mesh)	Li-ion and Li metal	Active Material	H_2O_2 [R]	0.5–2	2.13 – 4.26	50–80	N.A.	500	N.A.	-	-
[119]	Wet Shredding Sieving (30 mesh)	LCO with high Mn	Black Mass	No	2.8	N.A.	70–80	240	280	Li = 100% Co = 100% Mn = 100% Al = 61% Cu = 23%	No reducing agent	Long reaction time
[146]	Shredding	LCO NMC NCA	Active Material	$SO_{2(g)}$ [R] or $Na_2S_2O_5$ [R]	2	0.67 M (Na2S2O5) or Maintaining ORP<400 mV	70 (exothermic)	120	10	Li = 92% Co = 100% Mn = 100% Ni = 100% Al = 100%	Low cost reducing agent	Long reaction time Low solid to liquid ratio
[120]	Fresh Material	NMC	Active Material	Cu^0 [R]	1	1:1.2 Cu:Active Material Ratio	30	180	40	(Li, Mn, Ni, Co) > 95% Cu = 35%	Low cost reducing agent	Long reaction time Low solid to liquid ratio

[147]	Dismantling Crushing Sieving Alkaline leaching Reducing roasting Water-CO_2 Leaching	NMC LCO	Calcined Active Material	Lignite (in roasting) [R]	3.5	N.S.	85	180	200	Li = 85% Co = 99% Mn = 99% Ni = 99%	Low cost reducing agent	Complex pretreatment Long reaction time
[150]	Discharging Dismantling Calcining (600 °C)	NMC	Calcined Active Material	H_2O_2 [R]	4	2	90	120	25	Co = 99% Ni = 99% Mn = 97%	High leaching rates	Long reaction time Low solid to liquid ratio
[79]	Dismantling	NMC +NiMH	Anode and Cathode	H_2O_2 [R]	3	0.74	N.S.	240	N.S.	N.S.	-	-
[151]	Discharging Dismantling Alkaline Leaching (NaOH)	LCO	Anode and Cathodic Black Mass	Cu foil [R]	3	1.25g Cu/g Co	70	360	200	Co = 98% Li = 98% Cu = 9%	Low cost reducing agent	Long reaction time
[154]	Discharging Cryogenic Dismantling	LCO	Cathode and Anode	No	1.5	N.A.	80	60	20	Co = 37% Li = 55%	No reducing agent	Low leaching rates
[148]	Dismantling Manual separation Electrodes Shredding	LCO	Cathode	H_2O_2 [R]	0.4–1.5	0.43	20–80	60	100–200	Al = 55% Co = 80% Li = 95%	High solid to liquid ratio	Low leaching rates
[73]	Dismantling Thermal (600 °C)	LCO LMO LCM	Black Mass	No	1	N.A.	95	240	50	Co = 66% Li = 93% Ni = 96% Mn = 50%	No reducing agent	Low leaching rates
[135]	Crushing Magnetic	LCO NMC NCA	Cathode & Anode	$C_6H_8O_6$ [R]	2	0.03	80	300	50	Co = 100% Li = 95%	Low reducing	Long reaction time

	Separation Sieving (1 mm)										Ni = 105%	agent concentration	
[139]	Discharge Dismantling Manual separation Acid roasting (300 °C)	LCO NMC LMO	Black Mass	No	Acid Roasting	2 L/kg Solid	N.A.	300	30		Co = 80% Li = 79%		High temperature acid roasting
					Water Leaching	N.A.	N.A.	75	60	250			
				Glucose [R]	2nd Leaching	1 M H_2SO_4 + 0.5 M HNO_3	0.11	50	45	100	Cumul. Co = 90% Li = 93% Ni = 83% Mn = 78%	Low cost reducing agent	High temperature acid roasting
[149]	Thermal Treatment Crushing Sieving Alkaline Leaching (NaOH)	NMC	Black Mass	H_2O_2 [R]		5	4× Stoichiometric	90	120	125	N.S.	-	-
[140]	Discharging Dismantling Manual Separation Pyrolysis	LCO	Calcined Active Material	H_2O_2 [R]		2	2.13	80	60	50	Co = 99% Li = 99%	High efficiencies	High concentration of H_2O_2
[129]	Crushing/Sieving Drying	LCO, LNO & Polymer LIBs	Black Mass	No		2	N.A.	60	90	20	Co = 60% Li = 100%	No reducing agent Treatment of polymer LIBs	Low leaching rates
[134]	N.A.	NMC LCO LMO	Fresh Active Material	$Na_2S_2O_8$ [R]	Only $Na_2S_2O_8$	N.A.	0.33:1 R:NMC Ratio	90	1440	N.S.	Li = 60% Ni, Mn ,Co < 1%	Low cost reducing agent	Low leaching rates

[141]	Crushing Magnetic Separation Sieving	LCO	Cathode	H_2O_2 [R]		2	4.26	75	75	50	Co = 99% Li = 99%	High leaching rates	Applicable only to LCO
[46]	Inert Crushing Physical Separation Sieving	LFP	Anodic and Cathodic Black Mass	None	Fe/cathode Mass Ratio 0.15	1	N.A.	80	N.S.	N.S.	N.S.	-	-
[123]	Discharge Manual Separation Alkaline Leaching	LFP	Cathodic Black Mass	H_2O_2 [O]		0.3	H_2O_2/Li Molar Ratio 2.07	60	120	95	Li = 97% Fe = 0.03% P = 2%	High selectivity	Low value Fe product
[43]	N.A.	LFP NMC LCO	Active Material	H_2O_2 [O] & [R]		4	8.82	80	120–180	N.S.	Ni, Co, Mn = ~100% Li = 80%	Blend of LIBs	High concentration of H_2O_2
[85,126]	Discharge (SS Chips) Shredding Rare Earth Magnetic Alkaline Leaching Drying Screening Density Separation	LCO LMO NMC LFP	Spent Active Material and Plastic Waste	H_2O_2 [O] & [R]		4	6.40	70	120	285	Ni, Mn = ~90% Co = 80%	Blend of LIBs	High concentration of H_2O_2
[223]	Discharge Manual Separation Calcining (600 °C)	LFP	Cathode	None	pH = 2–4	N.S.	N.A.	60–95	120–240	12.5 – 25	N.S.	-	-
[122]	Dismantling Manual Separation	LFP	Cathode	H_2O_2 [O]		2	30% (N.S. if %w/w or %v/v)	60	120	100	Li = 82.1%	High solid to liquid ratio	Low value Fe product

	Alkaline<br>Leaching										Fe =<br>97.2%		
[93]	Discharge<br>Dismantling<br>Electrolyte<br>recovery<br>(Supercritical<br>CO_2)<br>Physical<br>Separation<br>Crushing<br>Calcining	LFP	Cathode	None		2.5	-	60	240	100	Li = 97%<br>Fe = 98%	No oxidizing<br>agent	Low value Fe<br>product
[159]	Discharge<br>Dismantling<br>Manual<br>Separation<br>Ultrasonic<br>Delamination	NMC	Black Mass	H_2O_2 [R]		1	0.43	N.S.	N.S.	N.S.	N.S.	-	-
[142]	Discharging<br>Drying<br>Crushing<br>Magnetic<br>Separation<br>Sieving (16 mesh)	NMC	Black Mass	H_2O_2 [R]	Na_2S as Cu<br>Precipitator	2	2.56	60	60	100	N.S.	-	-
[63]	Dismantling<br>Discharging<br>Drying<br>Manual<br>Separation<br>Crushing<br>Al Separation	NMC	Black Mass<br>with Al<br>Contamination	H_2O_2 [R]		2	2.13	60	120	100	Li, Ni,<br>Co,Mn =<br>~98%<br>Al = 89%	High<br>leaching rates	Long reaction<br>time

[143]	Discharging NaCl Manual Separation Vacuum Pyrolysis (200 °C) Alkaline leaching Calcination (500 °C) Grinding	LCO	Calcined Active Material	H_2O_2 [R]		2	3.41	70	60	20–50	Co = 99% Li = 99%	High leaching rates	Low solid to liquid ratio
[144]	Crushing-Sieving Magnetic Separation Eddy Current Separation	Mixed Oxides	Black Mass from Pretreatment Pilot Plant	H_2O_2 [R]								-	-
[133]	Discharging Manual Separation Drying	LCO	Cathode	H_2O_2 [R]	Sonication	1	2.13	60	60	20–50	Li, Co = ~100%	High leaching rates	Low solid to liquid ratio
[131]	Crushing Wet Scrubbing Drying	Mixed LIBs	Cathodic Black Mass	H_2O_2 [R]		2	4.26	30	180	75	Li = 99% Co = 97% Mn = 99% Cu = 97%	Mixed LIBs High leaching rates	Long reaction time

Table S2. Summary of purification and extraction processes in sulfate medium; here, EMD, D2EHPA, LCO, NMC, and LFP are electrolytic manganese dioxide, di-(2-ethylhexyl)phosphoric acid, lithium-cobalt oxide, lithium-nickel-manganese-cobalt oxide, and lithium-iron phosphate, respectively; N.A. stands for "Not Applicable"; N.S. stands for "Not Specified".

Reference(s)	Purification Process (Reagents)	Final Product(s)	Recovery Rate (%)	Comments
[160]	Cu Cementation (Steel Shots)<br>Fe Precipitation (NaOH)<br>Co Precipitation (NaClO)<br>Co Electrowining<br>Li Precipitation (H_3PO_4)	Cu(s)<br>$Fe(OH)_3$(s)<br>$Co(OH)_3$ or Co(s)<br>Li_3PO_4(s);	N.S.	
[145]	Metal Precipitation (NaOH)<br>Electrodialysis	(Mn, Ni, Co)$(OH)_2$(s)<br>LiOH(aq)	N.S.	
[132]	Alkalinization (NaOH)<br>Co Precipitation ($(NH_4)_2C_2O_4$)<br>Li Precipitation (Na_2CO_3)	CoC_2O_4(s)<br>Li_2CO_3(s)<br>Waste Na_2SO_4(aq)	Co = 94.7%<br>Li = 71%	
[127]	Fe, Al, Cu Precipitation (NaOH)<br>Co Solvent Extraction (Cyanex 272)<br>Co Precipitation (Na_2CO_3)<br>Ni,Mn Precipitation (Na_2CO_3)<br>Li Crystallization (Evaporation)	(Fe, Al)OH_3; $Cu(OH)_2$(s)<br>$Co(CO_3)$(s)<br>(Ni, Mn)CO_3(s)<br>Li_2CO_3(s)<br>Waste Na_2SO_4(aq)	Co = 98%<br>Li = 80%	
[137]	Co Solvent Extraction (D2EHPA)<br>Alkalinization (NaOH)<br>Co Hydrothermal Precipitation (CH_4N_2S)<br>Li Precipitation (Na_2CO_3)	CoS(s)<br>Li_2CO_3(s)<br>Waste Na_2SO_4(aq)	N.S.	

[72]	Fe Precipitation (Na_2SO_4) (pH = 2; 95 °C; 2 h)<br>Mn Precipitation ($(NH_4)_2(S_2O_8)$<br>Cu Precipitation (NaOH)<br>Co Solvent Extraction (P_5O_7)<br>Co Precipitation $(NH_4)_2(C_2O_4)$	$Na_2Fe_6(SO_4)_4$.12(OH)(s)<br>MnO_2(s)<br>$Cu(OH)_2$(s)<br>CoC_2O_4(s)<br>Waste(Li, Na, Ni)<br>Sulfate(aq)	Fe = 99%<br>Mn = 99%<br>Cu = 98,5%<br>Co = 98%	
[130]	Co Electrowinning<br>Mn Electrowinning	Co(s)<br>EMD (Electrolytic<br>Manganese Dioxide)<br>Waste Na_2SO_4(aq)	Co = 96%<br>Mn = 99%<br>Cu = 97%	pH 2–2.5 is a critical parameter for Co-metal and EMD.<br>*T* = 90 °C 200 A/m^2
[158]	Coprecipitation (NaOH + NH_4(OH))<br>NMC Synthesis-Sintering (Li_2CO_3(s))	NMC(s)<br>Waste Na_2SO_4(aq)	N.S.	
[161]	Co Solvent Extraction (PC88A)<br>Co Electrowinning	Co(s)<br>EMD (Electrolytic<br>Manganese Dioxide)<br>Waste Na_2SO_4(aq)	N.S.	Electrowinning at pH = 2 for, 50 mA/cm^2, and 50–60 °C
[119]	Mn Precipitation (NaClO)<br>Al Precipitation (NaOH)<br>Cu Precipitation (NaOH)<br>Co Precipitation (Na_2CO_3)<br>Li Precipitation (Na_2CO_3)	MnO_2(s)<br>$Al(OH)_3$(s)<br>$Cu(OH)_2$(s)<br>$CoCO_3$(s)<br>Li_2CO_3(s)	N.S.	
[146]	Metal Precipitation (NaOH)<br>Li Precipitation (Na_2CO_3)<br>Na Crystallization (Thermal)	(Ni, Mn, Co, Al)<br>Hydroxides<br>Li_2CO_3(s)<br>Na_2SO_4(s)	N.S.	

[153]	Co Solvent Extraction (Cyanex 272)<br>Co Electrowinning<br>Ni Electrowinning	Co(s)<br>Ni(s)<br>Li_2SO_4 Solution	Co = 100%	
[159]	Ni, Co, Mn Precipitation (Na_2CO_3+NH_3H_2O)<br>(Ni, Co, Mn)CO_3 Calcination<br>NMC Resynthesis (Li_2CO_3(s))	NMC(s)<br>Li_2SO_4 Solution	Co = 97%<br>Mn = 97%<br>Ni = 96%	163 mAh/g at 0.1 C; 94% retention after 50 cycles; 138.3 at 1 C
[147]	Li Solution Evaporation<br>Fe Precipitation (N.A.)<br>Ca and Mg Precipitation (N.A.)<br>Mn Solvent Extraction (D2EHPA)<br>Co Solvent Extraction (PC88A)	Li_2CO_3(s)<br>$MnSO_4$(s)<br>$CoSO_4$(s)<br>$NiSO_4$(s)	Li = 85%<br>Co = 98%<br>Mn = 99%<br>Ni = 97%	
[150]	Al, Cu Fe, Ca Precipitation (NaOH)<br>Ni, Co, Mn Solvent Extraction (D2EHPA)<br>Ni, Co, Mn Precipitation (NaOH+NH_3.H_2O)<br>Li Precipitation (Na_2CO_3)	Mixed Hydroxides<br>NMC(s)<br>Waste Li_2SO_4	Co = 99%<br>Mn = 100%<br>Ni = 85%	150 at 0.5 C; 145 at 1 C; retention 96% at 1 C after 50 cycles
[79]	Co Ni Solvent Extraction (D2EHPA)<br>Ni Co Ferrite Precipitation ($FeSO_4$+Citric Acid)	NiCo(ferrite)(s)<br>Waste Sulfate Solution	N.S.	
[151]	Co Precipitation ($C_2H_2O_4$)<br>Cu Solvent Extraction (Aldoxime based)<br>Co Solvent Extraction (Cyanex 272)<br>Li Precipitation (Na_2CO_3)<br>LiCo Precursor Precipitation (Sintering)	LCO(s)<br>$Al(OH)_3$(s)<br>Waste Sulfate Solution	Cu = 98%<br>Co = 97%<br>Li = 80%	136 mAh/g at 0.2 C

[154]	Co Solvent Extraction (Cyanex 272)<br>Co Electrowinning<br>Ni Electrowinning	$CoSO_4$ Solution<br>Spent Li_2SO_4 Solution	Co = 44%	
[148]	Al Precipitation (NH_4OH)<br>Co Solvent Extraction (Cyanex 272)	$CoSO_4$(s)<br>$Al(OH)_3$(s)<br>Li-rich Solution	Co = 85%	
[149]	Cu Precipitation (Na_2S)<br>Al and Fe Precipitation<br>Impurities Extraction (D2EHPA)<br>Mg Extraction (NaF)<br>(Co, Ni, Mn) Precipitation (Na_2CO_3 + NH_3H_2O)<br>(Co, Ni, Mn) $CO_{3(s)}$ Calcination<br>NMC Synthesis (Li_2CO_3(s) Sintering)	CuS(s)<br>NMC(s)<br>Sulfate Waste Solution<br>Precipitate (Hydroxides)	N.S.	152 mAh/g at 0.2 C; 94% retention at 1 C after 50th cycle
[84]	MnO_2 Electrolysis	MnO_2(s)<br>Sulfate Waste Solution	N.S.	
[155]	Co Solvent Extraction (PC-88A)<br>Ni Precipitation (NaOH)<br>Li Precipitation (Li_2CO_3(s))	$CoSO_4$(s)<br>$NiSO_4$(s)<br>Li_2CO_3(s)<br>Waste Na_2SO_4(s)	N.S.	
[134]	Li Precipitation (Na_2CO_3)	Li_2CO_3(s)<br>Wate Sulfate Solution<br>Co, Mn, Ni Oxides	N.S.	
[152]	Co Precipitation ($C_2H_2O_4$)	Li-rich Spent Solution<br>CoC_2O_4(s)	Co = 89%	

[46]	Oxidation (Fe Filing)<br>Neutralization (H_3PO_4)<br>Precipitation (Alkali)	Li_3PO_4(s)<br>Goethite (FeO(OH))<br>Waste SO_4^{2-} Brine	N.S.	
[123]	Alkalinization (NaOH)<br>Precipitation ($Na_2PO_4.12H_2O$)<br>$FePO_4$-C Calcining	Li_3PO_4(s)<br>Na_2SO_4(aq) Waste Brine<br>$FePO_4$(s)	Li = 95.6%<br>Fe = 99.98%<br>P = 98.0%	
[43]	Fe Precipitation (NaOH)<br>Li Precipitation (Na_2CO_3)<br>NMC Milling<br>NMC Sintering<br>LFP-C Calcining	$Fe(OH)_3$(s)<br>$LiNi_{1/3}Mn_{1/3}Co_{1/3}O_2$(s)<br>LFP(s)<br>$Na_2SO_4$(aq) Waste Brine	Li = 80%<br>Co, Ni, Mn = 100%	NMC = 130 mAh/g at C/3.4–82% retention after 50 cycles<br>173 mAh/g at C/13.7
[223]	Leaching (NaOH)<br>Li Precipitation (Na_3PO_4)	Li_3PO_4(s)<br>Fe_2O_3(s)<br>Na_2SO_4(aq) Waste Brine<br>Cu, Al and Fe-rich Solution	N.S.	
[122]	Fe Precipitation (NaOH)<br>Li Precipitation (Na_2CO_3)	Li_2CO_3(s)<br>Na_2SO_4(aq)<br>$Fe(OH)_3$(s)	Li = 82.1%	
[93]	Fe Precipitation (NH_3)<br>$FePO_4$ Carbo-Thermal Treatment<br>Li Solution Evaporation<br>Li Precipitation (Na_2CO_3)<br>Fe and Li Milling (Sucrose + Ethanol)<br>LFP Annealing (Ar + H_2)	LFP/C(s)<br>Na_2SO_4(aq) Waste Brine	N.S.	152 mAh/g at 0.2 C; 138 mAh/g at 1 C

[63]	Al Precipitation (NaOH)<br>$NMC(OH)_2$ Precipitation ($NaOH+NH_4OH$)<br>Li Precipitation (Na_2CO_3)	$Li_2CO_3(s)$<br>$Ni_{1/3}Mn_{1/3}Co_{1/3}(OH)_2(s)$<br>$Al(OH)_3(s)$<br>$Na_2SO_4(aq)$ Waste Brine	Li = 80%<br>Co, Ni, Mn = 100%	
[143]	Co Precipitation (NaOH)<br>$Co(OH)_2$ Calcination<br>Co Electrolysis (molten salt)<br>pH Adjustment (NaOH)<br>Li Precipitation (Na_2CO_3)	Co(s)<br>$Li_2CO_3(s)$<br>Spent $Na_2SO_4(aq)$	N.S.	
[144]	Fe Precipitation (NaOH)<br>Mn Al Cu Solvent Extraction (D2EHPA)<br>MnCo Ferrite Precipitation (NaOH Microwave)<br>Co Solvent Extraction (Cyanex 272)<br>Co Precipitation ($Na_2C_2O_4$ NaOH)<br>Evapo-crystallization<br>Li Precipitation (Na_2CO_3)	$MnCoFe_2O_4(s)$<br>$Co_3O_4(s)$<br>$Ni(OH)_2(s)$<br>$Na_2SO_4(s)$<br>$Li_2CO_3(s)$<br>Spent $Na_2SO_4(aq)$	Co = 100%<br>Li = 90%<br>Ni = 91%<br>Mn = 96%	Respective capacity for MnCo ferrite and NiO as anode materials reached 700 mAh/g and 400 mAh/g
[133]	Co Precipitation (NaOH)<br>Li Precipitation (CO_2)<br>Li Brine Evaporation<br>Li Saltwater Wash	$Co(OH)_2(s)$<br>$Li_2CO_3(s)$<br>$Na_2SO_4(aq)$	N.S.	
[85,126]	Fe Al Cu Precipitation (NaOH)<br>Co Ni Mn Concentration Adjustment (SO_4 Salt)<br>Co Ni Mn Precipitation (NaOH + NH_4OH)<br>$NMC(OH)_2$ Drying	NMC(s)<br>Fe, Al, Cu (OH)(s)<br>Spent $Na_2SO_4(aq)$	Ni, Mn, and Co > 90%	Electrochemical properties for NMC<br>152 mAh/g at 0.2C<br>137 mAh/g at 1C<br>No stability data

	Mixing Milling (Li_2CO_3)<br>NMC sintering<br>NMC Milling			
[131]	Fe and Mn precipitation ($(NH_4)_2S_2O_8$<br>Cu Solvent extraction (LIX 84IC)<br>$CuSO_4$ evaporation-crystallization<br>Co solvent extraction (Cyanex 272)<br>$CoSO_4$ evaporation-crystallization<br>Co electrowinning	MnO_2 + $Fe_2(SO_4)_3$(s)<br>$CuSO_4$(s)<br>$CoSO_4$(s)<br>Co(s)<br>Li_2SO_4 solution (aq)	Co = 98%<br>Cu = 100%	Co electrowinning:<br>Ti cathode + Pb-Sb anode at 200 A/m^2

Table S3. Summary of leaching processes with hydrochloric acid; here, LCO, LTO, NMC, LMO, LFP-C, and NMP are lithium-cobalt oxide, lithium-titanate-oxide, lithium-nickel-manganese-cobalt oxide, lithium-manganese-oxide, lithium-iron-phosphate, and n-methyl-2-pyrrolidone, respectively; N.A. stands for "Not Applicable"; N.S. stands for "Not Specified".

**Reference**	**Pretreatment**	**Treated Battery Type(s)**	**Leach Feed**	**Reducing [R] Oxidizing [O] Agent**	**Special Leaching Condition(s)**	**Acid Concentration (M)**	**Reducer/ Oxidizer Concentration (M)**	***T* (°C)**	**Leaching Time (min)**	**Solid Concentration (g/L)**	**Leaching Rate (%)**	**Pros**	**Cons**
[129]	Crushing/Sieving<br>Drying	LCO	Black Mass	No		2	N.A.	60	90	20	Co = 100%<br>Li = 100%	No reducing agent	Low solid to liquid ratio
[74]	Wet Shredding<br>Density Separation<br>Sieving (35 mesh)<br>Calcining (500 °C for 1 h)	Blend of LIBs	Active Material	No		1.75	N.A.	50	90	200	Co =99%<br>Mn = 99%	High solid to liquid ratio<br>No reducing agent	Complex pretreatment
[170]	Dismantling<br>Shredding<br>Sieving<br>Milling	NMC<br>LCO<br>LMO	Cathode and Anode	No	Electrochemical Acid Regeneration	4	N.A.	22	4 h	100	Co = 20%<br>Li = 38%<br>Mn = 31%<br>Ni = 17%	Electrochemical acid generation	Low leaching rate<br>Long reaction time

[169]	Crushing/Sieving<br>Binder Dissolution (NMP)<br>Foil Separation	LCO	Active Material	No		4	N.A.	80	60	N.S.	N.S.	-	-
[162]	Dismantling<br>Manual Separation<br>Electrodes Drying<br>Water Wash (Cathode Delamination)<br>Filtration/Water Wash<br>Drying	LCO	Black Mass	H_2O_2 [R]		3	0.75	80	120	18	N.S.	-	-
[42]	Dismantling<br>Manual Separation<br>Black Mass Scraping	LCO	Black Mass	No		4	N.A.	80	60	100	Co = 99%<br>Li = 99%	No reducing agent	Only applicable to LCO
[163]	Dismantling<br>Manual Separation<br>Binder Dissolution (NMP)<br>Foil Separation	LCO	Active Material	H_2O_2 [R]		3	3.5% (N.S. if %w/w or %v/v)	80	60	50	Co = 83%<br>Li = 83%		Low leaching rate
[171]	N.A.	LCO<br>LMO<br>NMC	Fresh Active Material	No		4	N.A.	80	60	20	Co = 99.5%<br>Li = 99.9%<br>Ni = 99.8%<br>Mn = 99.8%	No reducing agent	Not tested on spent batteries

[164]	Dismantling<br>Manual Separation<br>Binder Dissolution (NMP)<br>Carbon Flotation	LFP<br>LTO	Cathodic Black Mass	No		pH = 2	N.A.	80	480	N.S.	Fe = 100%<br>Ti = 0%<br>Li = 100%	Treatment of LTO & LFP	Long reaction time
[86]	N.S.	LFP<br>LMO	Cathode	H_2O_2 [O]		6.5	6.4	60	120	200	Li = 92%<br>Fe = 92%<br>Mn = 90%	High solid to liquid ratio	High acid and reducer concentration
[167]	Crushing<br>Thermal Treatment (300 °C)<br>Sieving<br>Calcining (700 °C)	LFP	Calcined Black Mass	None		N.A.	-	N.S.	N.S.	N.S.	N.S.	-	-
[166]	Dismantling<br>Manual Separation<br>Calcining (700 °C)	LFP	Calcined Black Mass	None		6	-	120	360	50	N.S.	-	-
[172]	Crushing/ Sieving (1 mm)<br>Magnetic Separation	Mixed Oxides	Fines from Crushing	O_2 [O]		4	0.133 L/s	50–80	120	50–100	N.S.	Simple pretreatment	Long reaction time
[19]	Spent Cathode<br>Al Delamination (NaOH)<br>Black Mass Milling (2 h)<br>Calcining (600 °C)	LFP	Calcined Black Mass	None		4	N.A.	N.S.	N.S.	N.S.	N.S.	-	-

Table 4. Summary of purification and extraction processes in chloride medium; here, LFP is lithium-iron-phosphate; N.A. stands for "Not Applicable"; N.S. stands for "Not Specified".

Reference	Purification Process (Reagents)	Final Product(s)	Recovery Rate (%)	Comments
[74]	Mn Precipitation (NaClO)<br>Al and Cu Precipitation (NaOH)<br>Co Precipitation (Na_2CO_3)<br>Li Precipitation (Na_2CO_3)<br>NaCl Crystallization (HCl)	Mn oxides(s)<br>$Cu(OH)_2$(s)<br>$Al(OH)_3$(s)<br>$CoCO_3$(s)<br>Li_2CO_3(s)<br>NaCl(s)	Co = 90%<br>Mn = 95%	
[169]	Co Precipitation (NaOH)	$Co(OH)_2$(s)<br>Li and Na solution	N.S.	
[162]	$Co(OH)_2$ Electrodeposition<br>Co Electrowinning (H_2SO_4)	Co(s)<br>Waste Chloride Solution<br>Waste Sulfate Solution	N.S.	
[42]	Co Solvent Extraction (PC-88A + H_2SO_4)<br>Li Precipitation (Na_2CO_3)	99.99% $CoSO_4$(s)<br>Li_2CO_3(s)<br>Waste Sulfate Solution<br>Waste Chloride Solution	Co = 99.99%<br>Li = 80%	Scrubbing coextracted Li in organic phase with $CoCl_2$ and HCl
[171]	Mn Precipitation ($KMnO_4$)<br>Ni Precipitation ($C_4H_8N_2O_2$ + NH_3)<br>$C_4H_8N_2O_2$ Regeneration (HCl)<br>Ni Re-precipitation (NaOH)<br>Co Precipitation (NaOH)<br>Li Precipitation (Na_2CO_3)	MnO_2(s)<br>$Ni(OH)_2$(s)<br>$Co(OH)_2$(s)<br>Li_2CO_3(s)<br>Waste Chloride Solution	Co = 97%<br>Li = 97%<br>Ni = 97%<br>Mn = 98%	

[164]	Fe Oxidation (H_2O_2)<br>Fe Precipitation (NH_3)<br>Purification (Ion Exchange)<br>Stripping (HCl)<br>Li Precipitation (Na_2CO_3)	Li_2CO_3(s)<br>$FePO_4.xH_2O$(s)<br>TiO_2(s)<br>Spent NaCl Brine	Li = 80%<br>Fe = 95.8%	
[86]	Fe Ionic Flotation ([Hbet][Tf_2N])<br>Mn Precipitation ($KMnO_4$)<br>Li Precipitation (Na_3PO_4)	$FeCl_3$<br>MnO_2(s) + Mn_2O_3(s)<br>Li_3PO_4(s)<br>Spent NaCl Brine	Li = 81%<br>Fe = 85%<br>Mn = 81%	
[167]	Fe Precipitation (NH_4)<br>$FePO_4$ Crystallization (H_3PO_4)<br>LFP Milling-Sintering (Li, C, N_2-H_2)<br>Li solution Evaporation<br>LiCl Heat Treatment (350–700 °C)	LFP(s)<br>LiCl(s)	N.S.	
[166]	Fe Precipitation (NH_4OH)<br>Crystallization (H_3PO_4)<br>Synthesis (LiOH + Sucrose)<br>Annealing (Ar+ H_2)	LFP/C(s)<br>Waste Brine<br>Spent LiCl Solution	N.S.	
[172]	Fe Al Cu Precipitation (NaOH)<br>Ni, Co, Mn Precipitation (Na_2CO_3)<br>Li Precipitation (Na_2CO_3)	Al, Fe, Cu $(OH)_2$(s)<br>(Ni, Mn, Co)CO_3(s)<br>Spent NaCl(aq)<br>Li_2CO_3(s)	Co = 95%<br>Mn = 92%<br>Ni = 95%	
[19]	$FePO_4.2H_2O$ Precipitation (NH_4OH + Na_3PO_4)<br>$LiFePO_4$ Synthesis (Calcination Li_2CO_3 + Glucose)	Al<br>$LiFePO_4$ (1)	Fe = 93%<br>Li = 82%	LFP (1) shows low capacities:<br>141 mAh/g at0.2 C<br>134 mAh/g at 1 C

	Li_3PO_4 Precipitation (NH_4OH)<br>Li_3PO_4 Solubilization (H_3PO_4)<br>Impurity Removal (NH_4OH)<br>$LiFePO_4$ Hydrothermal Synthesis ($FeSO_4$ + Ascorbic Acid)<br>Carbonation (Glucose + H_2 at 600 °C)	$LiFePO_4$ (2)<br>Glucose		LFP (2) shows good capacities:<br>157 mAh/g at0.2 C<br>146 mAh/g at 1 C<br>97% retention after 100 cycles

Table 5. Summary of leaching processes with nitric acid; here, LCO, NMC, LFP, and NMP are lithium-cobalt oxide, lithium-nickel-manganese-cobalt oxide, lithium-iron-phosphate, and n-methyl-2-pyrrolidone; N.A. stands for "Not Applicable"; N.S. stands for "Not Specified".

Reference	Pretreatment	Treated Battery Type(s)	Leach Feed	Reducing [R] or Oxidizing [O] Agent	Special Leaching Condition(s)	Acid Concentration (M)	Reducer Concentration	*T* (°C)	Leaching Time (min)	Solid Concentration (g/L or other)	Recovery Rate (%)	Pros	Cons
[75]	Discharging<br>Dismantling<br>Manual Separation<br>Binder Dissolution (NMP)<br>Calcination (700 °C for 5 h)<br>Alkaline Leaching	Blend of LIBs	Active Material	H_2O_2 [R]		1	0.85	70	120	N.S.	N.S.	-	-
[174]	Discharging<br>Dismantling<br>Manual Separation<br>Calcination (600 °C for 5 h)	Blend of LIBs	Calcined Active Material	Fe (grinding) [R]		1	1:1 Ratio, 30 min Milling	25	120	0.15	Co = 91%<br>Li = 77%<br>Mn = 100%<br>Ni = 100%	Low cost reducing agent	Low solid to liquid ratio
[176]	Discharging<br>Dismantling<br>Manual Separation	LCO	Black Mass	H_2O_2 [R]		3	4.3	N.S.	N.S.	N.S.	N.S.	-	-
[99]	Discharging<br>Dismantling<br>Manual Separation<br>Binder Dissolution	NMC	Calcined Active Material	H_2O_2 [R]		N.S.	1.2	120	120	21	N.S.	-	-

	(NMP)<br>Calcination (750 °C for 4 h)												
[173]	Crushing	Blend of LIBs	Whole Battery	No		2	N.A.	80	120	N.S.	Li = 100%<br>Co = low<br>Ni = low<br>Mn = 95%	Sample pretreatment	Low leaching rate
[175]	1st Thermal Treatment (150 °C for 1 h)<br>Shredding/Sieving<br>2nd Thermal Treatment<br>Sieving<br>Calcination (900 °C for 1 h)	LCO	Calcined Active Material	H_2O_2 [R]		1	0.725	75	30	20	Li = 99%<br>Co = 99%	High efficicencies	Complex pretreatment
[180]	Discharging<br>Manual Separation<br>Binder dissolution (NMP)<br>Calcination (800 °C for 2 h)	LCO	Calcined Active Material	H_2O_2 [R]		1	0.42	80	60	20	N.S.	-	-
[122]	Dismantling<br>Manual Separation<br>Alkaline Leaching	LFP	Cathodic Black Mass	H_2O_2 [O]		3	30%<br>(N.S. if %s or %v)	50	1440	1	Fe = 99%	-	Long reaction time<br>Low solid to liquid ratio

Table 6. Summary of purification and extraction processes in nitrate medium; here, LCO and NMC are lithium-cobalt oxide and lithium-nickel-manganese-cobalt oxide, respectively; N.A. stands for “Not Applicable”; N.S. stands for “Not Specified”.

Reference	Purification Process (Reagents)	Final Product(s)	Recovery Rate (%)	Comments

[75]	Impurities Precipitation (NaOH)<br>Ni Precipitation (Dimethylglyoxime)<br>Co Precipitation (NaOH)<br>Co Oxide Sintering	Co_3O_4(s) Nanoplates<br>Waste Solution	N.S.	
[176]	$CoFe_2O_4$ Precipitation ($FeCl_3.6H_2O$)<br>$Fe(OH)_3$ Precipitation ($NH_4.OH$)<br>Washing ($C_2H_7NO_2$)<br>Calcination	$CoFe_2O_4$(s)<br>Waste Lithium Solution	N.S.	$CoFe_2O_4$ used to oxidize methylene blue in textile industry effluent
[173]	Fe and Ni Precipitation (NaOH)<br>Mn Precipitation (NaOH)	Ni, Co and Fe Solid Residue<br>$Mn(OH)_2$(s)<br>Li and Na Spent Solution	N.S.	
[180]	Co Electrodeposition (LiOH)	LCO(s)<br>Waste Li Solution	N.S.	Electrodeposition parameters: 1 mA/cm^2; Ni anode; Pt cathode; T = 100; time 20 h<br>Refurbished material capacity: 130 mAh/g at 0.1 C 96% retention after 30 cycles at 0.1 C
[179]	Co Electrodeposition (LiOH)<br>$Co(OH)_2$ Calcining	Co_3O_4(s)<br>Waste Li Solution	N.S.	
[122]	Li Precipitation (Na_2CO_3)	Li_2CO_3(s)<br>$NaNO_3$(aq) Solution<br>$FePO_4$(s)	Li = 87%<br>Fe = 99%	
[177]	Concentration and pH Adjustment (Ni, Co, Na_2CO_3)<br>Li(NMC)CO_3 Precipitation (NH_4OH)	$NMC_{(621)}$(s)<br>Li_2CO_3(s)<br>Spent $NaNO_3$ Solution	N.S.	Discharge capacity:<br>1st cycle: 239 mAh/g at 0.1 C

	Li(NMC)CO_3 Milling (LiOH)<br>NMC Calcination<br>Li Mother Liquor Purification<br>Li_2CO_3 Precipitation (Na_2CO_3)			100th cycle: 194 mAh/g at 0.1 C<br>81.2% capacity retention after 100 cycle

Table S7. Summary of leaching processes with other mineral acids/alkaline; here, LCO, LFP, NMP, and EDTA are lithium-cobalt oxide, lithium-iron-phosphate, n-methyl-2-pyrrolidone, and ethylenediaminetetraacetic acid, respectively; N.A. stands for "Not Applicable"; N.S. stands for "Not Specified".

Reference	Pretreatment	Treated Battery Type(s)	Leach Feed	Reducing [R]/ Oxidizing [O] Agent	Leaching Agent	Special Leaching condition(s)	Acid Concentration (M)	Reducer/ Oxidizer Concentration (M)	T (°C)	Leaching Time (min)	Solid Concentration (g/L)	Leaching Rate (%)	Pros	Cons
[181]	Discharging<br>Dismantling<br>Manual Separation<br>Alkaline Leaching<br>Calcination (650 °C for 5 h)	LCO	Calcined Active Material	Glucose [R]	H_3PO_4		1.5	0.85	80	120	2	Co = 98%<br>Li = 100%	Low cost reducing agent	Low solid to liquid ratio
[182]	Discharging<br>Dismantling<br>Manual Separation<br>Calcination (300 °C)	LCO	Active Material	H_2O_2 [R]	H_3PO_4		0.38	0.85	90	60	8	Co = 99%<br>Li = 99%	Low reducing agent concentration	Low solid to liquid ratio
[184]	Dismantling<br>Manual Separation	LCO	Black Mass	No	HF		8.6	N.A.	75	120	20	Co = 98%<br>Li = 80%	No reducing agent	Dangerous Acid
[186]	Discharging<br>Dismantling<br>Manual Separation<br>Binder Dissolution (Cathode and Anode in NMP)<br>Drying	LCO	Black Mass	H_2O_2 [R]	H_3PO_4		0.7	1.7	40	60	50	Co = 99%<br>Li = 99%	Treatment of anode and cathode	Applicable only to LCO
[183]	Discharge<br>Dismantling<br>Manual Separation	LFP	Cathodic Black Mass	N.S.	H_3PO_4		0.5	N.A.	RT	40	25	Li and Fe = 95%	Room temperature reaction	Low value Fe product
[185]	Dismantling<br>Manual Separation	LFP	Cathodic Black Mass	H_2O_2 [O]	-	30 psi CO_2	N.A.	0.15	25	30	100	Li = 90%<br>Fe = 0.5%<br>P = 2.5%	Low cost reagent<br>High efficiency	Pressurized reactor

													Added-Value product	
[76]	Discharge<br>Dismantling<br>Manual Separation<br>Sonication in Water<br>Ball Milling (with EDTA)	LFP	Cathodic Black Mass	O_2 [O]	H_3PO_4	EDTA to LFP Ratio = 3:1 in Mechanical Activation	0.6	N.A.	RT	20	50	Li = 94%<br>Fe = 98%	High Efficiency	Low value Fe product
[187]	Crushing<br>Sieving (0.18 mm)<br>Gravity Separation	Mixed Ni and Co LIBs	Fines from Crushed Batteries	Ammonium Sulfite [R]	NH_3	Alkaline Leaching 1 M Ammonium Bicarbonate	1.5	1	60	180	20	Li = 60.5%<br>Ni = 99%<br>Cu = 99%<br>Co = 81%	Low cost reagent	Low efficiecies
[188]	Discharge<br>Manual Dismantling<br>Shredding	LFP	Sliced Cathode	$Na_2S_2O_8$ [O]	-			1.05 Excess	RT	20	300	Li = 99%<br>Fe = 0.05%<br>P = 0.59%<br>Al = 0.58%	Low cost reagent<br>High efficiency<br>Added-Value product	Highly corrosive oxidizing agent

Table S8. Summary of purification and extraction processes in other mineral acid/alkaline medium; here, LFP is lithium-iron-phosphate; N.A. stands for "Not Applicable"; N.S. stands for "Not Specified".

Reference	Purification Process (Reagents)	Final Products	Recovery Rate (%)	Comments
[182]	Co Precipitation (Oxalic Acid)<br>Li Precipitation (NaOH + Ethanol)	CoC_2O_4(s)<br>Spent Na Solution<br>Li_3PO_4(s)	Li = 88%<br>Co = 99%	
[183]	Reflux Evaporation<br>Washing (Ethanol)<br>Carbo-thermal Reduction (Li_2CO_3)<br>Milling<br>Sintering	LFP/C(s)<br>LiH_2PO_4(s)	Li = 75%	159 mAh/g at 0.1 C<br>89.33 mAh/g at 20 C<br>95% retention after 500 cycles at 5 C

[76]	Fe Precipitation/Reflux Evaporation<br>Li Precipitation (NaOH)	$FePO_4.2H_2O$(s)<br>Li_3PO_4(s)<br>Na_3PO_4(aq)	Li = 83%<br>Fe = 93%	
[188]	Leaching Residue Sieving<br>Filtrate Evaporation<br>Li Precipitation (Na_2CO_3)<br>Crystallization	$FePO_4$(s)<br>Al<br>Li_2CO_3(s)<br>$Na_2SO_4.10H_2O$(s)	N.S.	

Table S9. Summary of leaching processes with organic acids; here, LCO, NMC, LMO, NMP, and PVC are lithium-cobalt oxide, lithium-nickel-manganese-cobalt oxide, lithium- manganese oxide, lithium-iron-phosphate, n-methyl-2-pyrrolidone, and polyvinyl chloride, respectively; N.A. stands for "Not Applicable"; N.S. stands for "Not Specified".

Reference(s)	Pretreatment	Treated Battery Type(s)	Leach Feed	Reducing [R]/ Oxidizing [O] Agent	Acid	Acid Concentration (M)	Reducer Concentration (M)	*T* (°C)	Leaching Time (min)	Solid Concentration (g/L)	Recovery Rate (%)	Pros	Cons
[196]	Discharge<br>Manual Dismantling<br>Cathode Separation<br>Binder Dissolution (NMP)<br>Drying (100 °C for 1 h)	LCO	Active Material	PVC [R]	PVC (HCl)	3:1 PVC:LCO	3:1 PVC:LCO	350	30	16	Co = 95%<br>Li = 98%	Recycling of another plastic waste	High temperature<br>Low solid to liquid ratio
[206]	Discharge<br>Crushing<br>Sieving	LCO	Cathode and Anode	No	Oxalic	1	N.A.	95	150	15	Co = 97%<br>Li = 98%	No reducing agent	Expensive acid<br>Low solid to liquid ratio
[191]	Discharge<br>Dismantling<br>Cathode Separation<br>Black Mass Stripping<br>Grinding<br>Calcining (700 °C for 2 h)	LCO	Calcined Active Material	H_2O_2 [R]	Formic	01:10	2.56	70	20	20	Co = 100%<br>Li = 100%	High efficiencies	High concentration of H_2O_2

[197]	Discharge<br>Dismantling<br>Cathode Separation<br>Binder Dissolution (NMP)<br>Al Separation<br>Calcining (700 °C for 2 h)	LCO	Calcined Active Material	Ascorbic Acid [R]	Glycine	0.5	0.02	80	360	2	Co = 95%	Low acid and reductant concentration	Long process time<br>Very Low solid to liquid ratio
[208]	Discharge<br>Dismantling<br>Cathode Separation<br>Binder Dissolution (NMP)<br>Al Separation	NMC<br>LCO	Active Material	H_2O_2 [R]	Tartaric Acid	2	1.71	70	30	17	Co = 99%<br>Li = 99%<br>Ni = 99%<br>Mn = 99%	High efficiencies	Low solid to liquid ratio
[104,178,198]	Discharge (Na_2SO_4 saturated)<br>Dismantling<br>Cathode Separation<br>Alkaline Leaching (2 M NaOH 2 h)<br>Drying Calcining (700 °C for 5 h)<br>Grinding	NMC<br>LCO<br>LMO	Calcined Active Material	H_2O_2 [R]	Citric Acid	0.5	0.64	90	60	20	Co = 100%<br>Li = 99%<br>Ni = 99%<br>Mn = 95%	Efficient for various LIBs type	Low solid to liquid ratio
[90,199]	Discharge<br>Dismantling<br>Cathode Separation<br>Binder Dissolution (NMP)<br>Drying Calcining (45 °C for 1 h)<br>Grinding	LCO	Calcined Active Material	Ascorbic Acid		1.25		70	20	25	Co = 95%<br>Li = 98.5%	High efficiencies<br>Two-in-one chemical	Expensive acid<br>Low solid to liquid ratio
[100]	Discharge<br>Dismantling<br>Cathode Separation<br>Binder Dissolution (NMP)<br>Grinding<br>Calcining (700 °C for 2 h)	LCO	Calcined Active Material	Ascorbic Acid [R]	Maleic Acid	0.1	0.02	80	360	2	Co = 91%<br>Li = 99%	Low acid and reductant concentration	Low solid to liquid ratio<br>Long reaction time

[200]	Discharge<br>Dismantling<br>Plastic & Casing Removal	NMC	Anode and Cathode	Lemon Juice [R]		Pure Lemon Juice		90	20	50	Co = 94%<br>Li = 100%<br>Ni = 98%<br>Mn = 99%		
[201]	Discharge<br>Dismantling<br>Binder Dissolution (NMP)<br>Calcining (600 °C for 4 h)	NMC	Calcined Active Material	H_2O_2 [R]	Citric acid	1	5.12	60	40	80	Weight Leaching = 99%	Relatively High solid to liquid ratio	High concentration of H_2O_2
[189]	Discharge (Na_2SO_4 sat)<br>Dismantling<br>Cathode Separation<br>Binder Dissolution (NMP)<br>Drying Calcining (700 °C for 2 h)<br>Grinding	LCO	Calcined Active Material	H_2O_2 [R]	Citric acid	2	0.88	70	80	50	Co = 98%<br>Li = 99%	Low concentration of H_2O_2	Limited to LCO
[189]	Discharge (Na_2SO_4 sat)<br>Dismantling<br>Cathode Separation<br>Binder Dissolution (NMP)<br>Drying Calcining (700 °C for 2 h)<br>Grinding	LCO	Calcined Active Material	Green Tea Waste [R]	Citric Acid	1.5	0.59	90	120	30	Co = 96%<br>Li = 98%	Reuse of a waste	Availability of tea waste limited
[190]	Discharge<br>Dismantling<br>Cathode Separation<br>Black Mass Stripping<br>Thermal (700 °C for 6 h)	LCO	Calcined Active Material	H_2O_2 [R]	Citric Acid	2	0.53	60	120	30	Co = 81%<br>Li = 92%	Low concentration of H_2O_2	Low efficiencies
[195]	Discharge<br>Dismantling<br>Cathode Separation	NMC	Black Mass	H_2O_2 [R]	Acetic Acid	3.5	1.71	60	60	40	Co = 94%<br>Li = 100%<br>Ni = 96%<br>Mn = 93%	Inexpensive acid	Relatively low efficiencies

[193]	Discharge<br>Dismantling<br>Cathode Separation	NMC	Cathode	H_2O_2 [R]	Formic Acid	2	6	60	60	50	Co = 37%<br>Li = 100%<br>Ni = 50%<br>Mn = 62%<br>Al = 4.5%	Simple pretreatment	Low recovery rate
[202]	Discharge (5% NaCl)<br>Dismantling<br>Cathode Separation<br>Binder Dissolution (NMP)	LCO	Active Material	Fe-PVC [R]	PVC (HCl)	1:1 LCO:PVC	Ratio LCO:Fe = 1:2	N.S.	720	Dry grinding then water washing	Co = 8.1%<br>Li = 100%	Recycling of another plastic waste	Long reaction time
[203]	Discharge<br>Dismantling<br>Cathode Separation<br>Black Mass Stripping<br>Thermal (700 °C for 2 h)	LCO	Calcined Active Material	Ascorbic Acid [R]	Citric Acid	0.1	0.02	80	360	2	Co = 80%<br>Li = 100%	Low acid and reductant concentration	Long reaction time<br>Low solid to liquid ratio
[77]	Discharge<br>Dismantling	LFP	Cathode	H_2O_2 [O]	Acetic Acid	0.8	2.56	50	30	120	Li = 95%<br>Fe = 6%	High solid to liquid ratio	Higher concentration of H_2O_2
[207]	Discharge<br>Dismantling<br>Binder dissolution (NMP)<br>Calcining (400 °C)<br>Grinding	LFP	Calcined Active Material	None	Oxalic Acid	0.3	N.A.	80	60	60	Li = 98%<br>Fe = 92%	No oxidizing agent	Low value Fe product
[141]	Crushing<br>Magnetic Separation<br>Sieving	LCO	Fine, Non-mag Material	None	Oxalic Acid	3	N.A.	80	120	50	Co = 0.25%<br>Li = 100%	Simple pretreatment	Low recovery rate
[204]	Manual Dismantling/Separation	LFP	Cathode	None	Citric Acid; Mechano-Chemical Process	20 g Citric Acid/g LFP	N.A.	N.S.	480	50	Li = 97.8%<br>Fe = 95.6%	No oxidizing agent	Long reaction time

Table S10. Summary of purification and extraction processes in organic acid solutions; here, LCO, NMC, and DADMAC are lithium-cobalt oxide, lithium-nickel-manganese-cobalt oxide, and diallyldimethylammonium chloride; N.A. stands for "Not Applicable"; N.S. stands for "Not Specified".

Reference	Purification Process (Reagents)	Final Product(s)	Recovery Rate (%)	Comments
[191]	Co Precipitation (NaOH)<br>Li Precipitation (NaF)	LiF(aq)<br>$Co(OH)_2$	Co = 100%<br>Li = 100%	
[104]	Sol-gel NMC Precipitation	NMC(s)	N.S.	150 mAh/g at 0.2 C; 94% retention after 160 cycles 0.2 C
[200]	Co Precipitation ($H_2C_2O_4$)<br>Mn Precipitation (Na_2CO_3)<br>Ni Precipitation (Na_2CO_3)<br>Li Precipitation (Na_2CO_3)	CoC_2O_4(s)<br>$MnCO_3$(s)<br>$NiCO_3$(s)<br>Li_2CO_3(s)	N.S.	
[201]	NMC Sol-gel	NMC(s)	N.S.	147 mAh/g at 0.1 C; 93% retention after 50 cycles
[189]	Co Precipitation ($H_2C_2O_4$)<br>Li Precipitation ($N_{a2}CO_3$)	CoC_2O_4(s)<br>Li_2CO_3(s)	Co = 99%<br>Li = 93%	
[202]	Co/Fe Calcination	$CoFe_4O_6$(s)	Co = 92%<br>Li = 100%	
[77]	Al Foil Recovery<br>Li Solution Evaporation<br>Fe Impurities Precipitation (NaOH)<br>Li Precipitation (Na_2CO_3)<br>Carbothermal Reduction (Glucose Ethanol)	Al(s)<br>$Fe(OH)_3$(s)<br>Spent CH_3COONa(aq)<br>LFP-C(s)	Al = 99.5%<br>Fe = 99.1%<br>Li = 84.8%	
[207]	FeC_2O_4(s) Calcining<br>Li Extraction (Ionic Sieve)<br>$Ca_3(PO_4)_2$ Precipitation ($Ca(OH)_2$)	FeO; Fe_2O_3(s)<br>Li(aq)<br>Spent Water<br>$Ca_3(PO_4)_2$(s)	N.S.	
[205]	Hydrothermal Precipitation (NH_4OH, Urea, DADMAC)<br>Co Calcination<br>Sintering (Li_2CO_3)	LCO<br>Spent Na Solution	100%	140 mAh/g at 0.1 C<br>123 mAh/g at 1 C

[204]	Evaporation<br>Fe precipitation (NaOH)<br>Li Precipitation (Na_2CO_3)	$Fe(OH)_3$(s)<br>Li_2CO_3(s)<br>Spent Solution Na Citrate/Phosphate	Li = 89.95%	

Table S11. Summary of direct recycling processes; here, LCO, NMC, NCO, LFP, NMP, DMAC, DMC, EC, MIBC, and CMC are for lithium-cobalt oxide, lithium-nickel-manganese-cobalt oxide, lithium-nickel-cobalt oxide, lithium-iron-phosphate, n-methyl-2-pyrrolidone, dimethylacetamide, dimethylene carbonate, ethylene carbonate, methylisobutylcarbinol, and carboxymethyl cellulose; N.A. stands for "Not Applicable"; N.S. stands for "Not Specified".

Reference	Treated Battery Type(s)	Process Schemes	*T* (°C)/Other parameters	Final Product(s)	Re-evaluated Capacity (mAh/g)
[89]	NMC	Discharging<br>Dismantling<br>Alkali leaching (NaOH)<br>Heating	600 °C (5 h)	NMC Zinc O_2 Battery	N.A.
[209]	Any LIBs	Ultrasound Treatment	[900; 2000] kHz (0.12–0.5 h)	Active Material	N.S.
[212]	NCA	Dismantling<br>Cathode Separation<br>Cleaning (0.5–10 g/L NH_3 + Alcohol)<br>Calcining	30 °C (0.3 – 1 h)<br>600–700 °C (2–7 h)	NCA	99% of pristine material capacity;<br>191 mAh/g at 0.15 C

[58]	Any LIBs	Dismantling<br>Cathode Separation<br>Solvent Washing<br>Milling<br>Sieving<br>Autoclave (LiOH)<br>Sintering<br>Cooling (CO_2)	250 – 900 °C (7–12 h)	Active Material	N.S.
[108]	Any LIBs	Dismantling<br>Cathode Separation<br>Binder Dissolution (NMP)<br>Flotation (Limonene + Torpineol)<br>Calcining (Li_2CO_3)	850 °C (12 h)	LCO	95% retention after 50 cycles
[219]	LCO	Discharging<br>Dismantling<br>Cathode Separation<br>Al Foil Recovery<br>Electrochemical Dissolution/Deposition (LiOH + KOH)	40–100 °C; 1 mA/cm^2	LCO	Reactions proposed are wrong. Not oxidative but reductive<br>135 mAh/g at 0.2 C; 96% retention after 50 cycles
[220]	LCO	Discharging<br>Dismantling<br>Cathode Separation		LCO	126.7 mAh/g at 0.2 C; 97.2 % retention after 50 cycles

		Al Foil Recovery<br>Electrochemical Pulsating ($LiOH$)	50 kV SS<br>Anode (0.5 h)		
[60,214]	Lithium Oxide	Crushing<br>Sieving<br>C Flotation<br>Calcining ($LiOH$)	500–800 °C (1 h)	LCO<br>NCO<br>NMC	135 mAh/g at 0.1 C (LCO)<br>170 mAh/g at0.1 C (NCO)<br>175 mAh/g at0.1 C (NMC)
[216]	LCO	Dismantling<br>Cathode Separation<br>Washing (DMC)<br>Binder Dissolution (NMP)<br>Hydrothermal ($LiOH$ + Li_2SO_4)<br>Annealing	50 min<br>220 °C (4 h)<br>800 °C (4 h)	LCO	150 mAh/g at 0.1 C<br>93% retention after 100 cycles
[107]	LCO<br>or other LIBs	Dismantling<br>Harvesting Active Material<br>Al and Cu Leaching (NH_4OH + $LiOH$+O_2)<br>Drying	5 M NH_4OH – 1 M $LiOH$ (12 h)	Active Material	N.S.
[224]	LFP	Discharge<br>Dismantling<br>Components Separation	Manual<br>Manual	LFP-C	148 mAh/g at 0.1 C<br>126 mAh/g at 1 C

		Cathode Heat Treatment (N_2)<br>Al Foil Removal (H_2O)<br>Milling ($FeC_2O_4.2H_2O$, Li_2CO_3, $(NH_4)HPO_4$<br>Thermal Treatment (N_2)	550 °C<br><br>Ultrasound<br>Vacuum<br>700 °C		99% retention at 1 C after 50 cycles
[116]	LFP	Discharge<br>Dismantling<br>Components Separation<br>Alkaline Leaching (NaOH)<br>Binder Dissolution (NMP)<br>Relithiation (LiOH)<br>Annealing (N_2)	Manual<br>Manual<br>5 M NaOH<br><br>180 °C/0.5 M LiOH<br>350 °C + 650 °C	LFP-C	130 mAh/g (charge rate not available)<br>99.6% retention after 100 cycles
[98]	LFP	Discharge<br>Dismantling<br>Components Separation<br>Binder Dissolution (DMAC)<br>Doped Annealing (N_2 + Fresh LFP)	Manual<br>Manual<br>30 °C/S:L = 1:10<br>700 °C/Doping Ratio (1:9)	LFP-C	Approximately 144 mAh/g at 0.1 C<br>130 mAh/g at1 C
[217]	LFP	Discharge<br>Dismantling<br>Components Separation<br>Black Mass Scraping	0.5 V<br>Manual<br>Manual<br>Manual	LFP-C	Approximately 150 mAh/g (charge rate not specified)

		Chemical Lithiation (LiI in Acetonitrile)<br>Drying/Grinding	1 M LiI		
[58]	LFP	Dismantling<br>Component Separation<br>Black Mass Harvesting<br>Chemical Lithiation (LiI in EC:DEC))<br>Annealing	1 M LiI<br>N/S	LFP-C	N.S.
[97,213]	LFP	Discharge<br>Dismantling<br>Alkaline Wash (NaOH)<br>Components Separation<br>Cathode Alkaline Wash (NaOH)<br>Grinding Sieving<br>Annealing (H_2 + Ar + Li_2CO_3)	2 V<br>pH = 10–11<br>Mechanical<br>650 °C	LFP-C	147 mAh/g at 0.2 C<br>132 at 1 C<br>95.3% retention after 100 cycles at 0.2 C
[69]	LFP	Dismantling<br>Components Separation<br>Thermal Treatment (N_2)<br>Al Separation<br>Grinding/Sieving	Manual<br>Manual<br>500 °C	LFP-C	136 mAh/g at 0.1 C
[225]	LFP	Discharge<br>Dismantling	0.5 V<br>Manual	LFP-C	150 mAh/g at 0.1 C<br>145 mAh/g at 1 C

		Binder Dissolution (DMC)<br>Drying<br>Components Separation<br>Crushing/Sieving<br>Annealing (N2)	95 °C<br><br><br><br>200 °C + 700 °C		147.3 mAh/g at C/3<br>95% retention after 100 cycles at C/3
[226]	NMC	Opening<br>Electrolyte Washing (DMC)<br>Binder Dissolution (NMP)<br>Manual Sorting<br>Relithiation (LiNO3 + LiOH)<br>NMC Sintering (O2)	Sonication<br><br>Molten Salt<br>300 °C; 2–4 h<br>850 °C for 4 h	NMC (523)	150 mAh/g at 0.1 C<br>149.3 mAh/g at 1 C<br>136 mAh/g after 100 cycles at 1 C<br>90% retention after 100 cycles at 1 C
[227]	LFP	Discharge (NaCl 5%)<br>Crushing<br>Drying<br>Pyrolysis (N_2)<br>Crushing<br>2 Stage Screening<br>Fines C Flotation (Kerosene, MIBC, CMC)	120 °C<br>550 °C (2 h)<br><br>1 mm; 0.25 mm<br>1 Rougher; 2 Cleaner; 3 Scavenger	LFP<br>Carbon<br>Cu-Al<br>Electrolyte	128 mAh/g at 0.2 C<br>126 mAh/g at 0.2 C after 100 cycles<br>98.6% retention

[228]	LFP	Discharge-Perforation<br>Automatic Dismantling<br>Thermal Treatment<br>Crushing<br>Sieving<br>Eddy Current Separator<br>Pneumatic Separator	300 °C (2 h)<br><br>4 mm; 0.2 mm<br>Repulsed = Al and Cu<br>O/F = Al; U/F = Cu	LFP + Carbon<br>Al<br>Cu	N.A.

© 2020 by the authors. Submitted for possible open access publication under the terms and conditions of the Creative Commons Attribution (CC BY) license (http://creativecommons.org/licenses/by/4.0/).
